# Supplementary material for: Tracking excited state decay mechanisms of pyrimidine nucleosides in real time
Source: Nat Commun. 2021 Dec 14;12:7285. doi: 10.1038/s41467-021-27535-7 (PMC8671501; doi:10.1038/s41467-021-27535-7)
Supplement: Supplementary file 1 — Supplementary Information [file 41467_2021_27535_MOESM1_ESM.pdf]

Supplementary Information

"Tracking excited state decay mechanisms of pyrimidine nucleosides in real time"

Rocío Borrego-Varillas[1,†], Artur Nenov[2,†], Piotr Kabacinski[3], Irene Conti[2], Lucia Ganzer[3], Aurelio Oriana[3], Vishal Jaiswal Kumar[2], Ines Delfino[4], Oliver Weingart[5], Cristian Manzoni[1], Ivan Rivalta[2], Marco Garavelli[2,*] and Giulio Cerullo[1,3,*]

[1] IFN-CNR, Piazza Leonardo da Vinci 32, I-20133 Milano, Italy

[2] Dipartimento di Chimica Industriale, Università degli Studi di Bologna, Viale del Risorgimento 4, I-40136 Bologna, Italy

[3] Dipartimento di Fisica, Politecnico di Milano, Piazza Leonardo da Vinci 32, I-20133 Milano, Italy

[4] Dipartimento di Scienze Ecologiche e Biologiche, Università della Tuscia, Largo dell'Università snc, I-01100 Viterbo, Italy

[5] Heinrich-Heine-Universität Dusseldorf, Institut für Theoretische Chemie und Computerchemie, Düsseldorf, Germany

Contents

1 Bibliographic survey on the decay times and the proposed photoinduced decay mechanisms in pyrimidines 1

1.1 Direct internal conversion (IC) to the ground state ($\pi\pi^\star \rightarrow S_0$) 3

1.2 IC to an intermediate dark state ($\pi\pi^\star \rightarrow n\pi^\star$) 3

1.2.1 Direct IC to the ground state ($n\pi^\star \rightarrow S_0$) 4

1.2.2 Decay to a triplet state ($n\pi^\star \rightarrow T_1 \rightarrow S_0$) 4

1.2.3 Re-population of the $\pi\pi^\star$ state ($n\pi^\star \rightarrow \pi\pi^\star \rightarrow S_0$) . . . 4

1.3 ISC from the bright $\pi\pi^\star$ to an intermediate triplet $^3n\pi^\star$ state ($\pi\pi^\star \rightarrow {^3n\pi^\star}$) 4

2 Theoretical background and protocols for acquisition of simulation parameters 5
2.1 Classical molecular dynamics 5
2.2 QM/MM geometry refinement 5
2.3 Wigner sampling 6
2.4 Linear absorption spectroscopy 7
2.5 Non-adiabatic mixed quantum-classical dynamics simulations . 8
2.5.1 Technical details on the SS-CASPT2 production dynamics 8
2.5.2 Technical details on the MS- and XMS-CASPT2 benchmarking dynamics on Uridine 11
2.6 How to estimate the $n\pi^*$ involvement 14
2.7 Simulation of transient absorption spectra 17

3 Simulation results 19
3.1 Wigner sampling analysis 19
3.1.1 Uridine (Urd) 19
3.1.2 5-methyl-uridine (5mUrd) 21
3.1.3 Comparison 23
3.2 Linear absorption spectra 25
3.2.1 Uridine (Urd) 25
3.2.2 5-methyl-uridine (5mUrd) 25
3.2.3 Comparison 29
3.3 SS-CASPT2/SA-2-CASSCF(10,8) trajectory plots 30
3.3.1 Uridine (Urd) 30
3.3.2 5-methyl-uridine (5mUrd) 33
3.4 Comparison of SS-, MS- and XMS-CASPT2 dynamics in Uridine 36
3.5 MS-CASPT2/SA-6-CASSCF(14,10) plots along trajectories: adiabatic vs. diabatic picture 42
3.5.1 Uridine (Urd) 43
3.5.2 5-methyl-uridine (5mUrd) 46
3.6 Conical intersections 49
3.6.1 Uridine (Urd) 49
3.6.2 5-methyl-uridine (5mUrd) 49
3.7 Analysis of vibrational coherences 51

4 Materials and Steady-State Spectroscopy 58

5 Transient absorption spectroscopy 59
5.1 Transient absorption spectra 59
5.2 Measurements on the pure solvent 60

5.3 Measurements at magic angle and orthogonal polarizations . . 61
5.4 Components of the simulated transient spectra 65
5.5 Measurements in the picosecond time scale 67

6 Data analysis 69
6.1 Fits at selected wavelengths 69
6.2 Global analysis 71
6.2.1 Uridine (Urd) 71
6.2.2 5-Methyluridine (5mUrd) 72
6.3 Excited-state vibrational modes 75

7 Analysis of the potential energy surfaces 76
7.1 Uridine (Urd) 76
7.2 5-methyl-uridine (5mUrd) 77

8 References 80

Supplementary Note 1:

Bibliographic survey on the decay times and the proposed photoinduced decay mechanisms in pyrimidines

	τ_1(fs)	τ_2 (fs)	τ_{long} (ps)	Technique, IRF (fs)	Solvent	Ref.
Thy	195	633	–	FU, 330 fs	H_2O	[Gus06]
	<150	580	–	FU, 150 fs	H_2O	[Gus02]
	–	2800	30	TAS, 200 fs	PBS	[Har07]
	70	410	–	TRPES, 220 fs	H_2O	[Buc15]
	–	360	–	TRPES, 160 fs	H_2O	[Eri19]
	–	540	–	TAS, 210 fs	H_2O	[Pec01]
	–	–	300	TAS, TRIS 1.5 ps	D_2O	[Pil18]
	68	130-2210	–	TAS, 10 fs	H_2O	[Xue16]
	630	1740	54	TAS, 10 fs	H_2O	[Pro16]
	1200	1300	–	TAS, 180 fs	H_2O	[Reu00]
Thd	120	390	–	TRPES, 220 fs	H_2O	[Buc15]
	–	390	–	TRPES, 160 fs	H_2O	[Eri19]
	150	720	–	FU, 450 fs	H_2O	[Oni02]
	–	700	–	FU, 360 fs	PBS	[Peo01]
	150	760	4000	TAS 200 fs, TRF 300 fs	H_2O	[Kwo08]
	<150	690	–	FU, 150 fs	H_2O	[Guv02]
	540	–	–	TAS, 200 fs	H_2O	[Pec01]
TMP	200	1100	–	FU, 150 fs	H_2O	[Gus02]
	210	1070	–	FU, 450 fs	H_2O	[Oni02]

Table 1: Decay time constants found in literature for thymine, thymidine and thymidine monophosphate

Over the past years many experimental studies have been performed to shed light in the fate of photoexcitations in DNA/RNA [Mid09, Koh10, Cre04, Sai07, Che14], which has relevant implications in the preservation of genetic code. Pioneering transient absorption (TA) spectroscopy studies by Kohler's group [Pec00, Pec01] revealed sub-picosecond decay times for nucleosides in water. These results were further confirmed by femtosecond fluorescence up-conversion (FU) measurements [Peo01, Gus11]. The decay channels of pyrimidines and the involvement of $n\pi^\star$ excited states are still

	τ_1(fs)	τ_2 (fs)	τ_{long} (ps)	Technique, IRF	Solvent	Ref.
Ura	96	–	–	FU, 330 fs	H_2O	[Gus06]
	550	1900	24	TAS, 200 fs	PBS	[Har07]
	54	110-1640	–	TAS, 10 fs	H_2O	[Xue16]
	900	1000	–	TAS, 180 fs	H_2O	[Reu00]
Urd	210	–	–	TAS, 200 fs	PBS	[Coh04]
UMP	200	430	365	TAS, 300 fs	PBS	[Bri19]

Table 2: Decay time constants found in literature for uracil, uridine and uridine monophosphate

the matter of a heated debate and several hypotheses exist [Mid09, Cre04, Imp16].

While most theoretical studies in gas phase assign the lowest excited state (ES) to a $n\pi^\star$ transition in uracil (Ura), 5-methyl-uracil (5mUra), also known as thymine (Thy) and their respective nucleosides uridine (Urd) and 5-methyl-uridine (5mUrd), there is a general agreement that the solvent modulates the interplay between the $\pi\pi^\star$ and $n\pi^\star$ states, destabilizing the $n\pi^\star$ state with respect to the bright $\pi\pi^\star$. Due to the dark nature of the $n\pi^\star$ state, it is not easy to obtain a direct experimental proof of its energy shift in order to assess its involvement in the ES deactivation [Imp16, He04].

Among the five solvated nucleosides, pyrimidines present longer lifetimes than purines [Oni02, Har07, Imp16, Gus02] Usually, longer time constants have been reported for 5mUra and 5mUrd, compared to Ura and Urd [Pec01, Peo01, Gus02, Har07, Gus06, Pol14, Kwo08, Pil18, Xue16, Kob12, Pro16]: this fact is in itself intriguing, given the close molecular structure and similar potential energy surfaces of both systems[Pep17]. Experimental TA and FU spectroscopy measurements agree on describing their excited state decays with one or two sub-picosecond components and a further component of several picoseconds [Pec01, Peo01, Gus11, Gus02, Oni02, Har07, Gus06, Pol14, Xue16, Kob12, Pro16, Coh04, Buc15]. Additionally, significantly longer lifetimes have been reported for pyrimidine nucleosides with respect to free bases, suggesting that in pyrimidine systems sugar substitution could alter significantly the lifetime of an excited nucleobase [Con18, Har07].

In this complex context, three hypotheses have been proposed for the assignment of the experimental time constants.

1.1 Direct internal conversion (IC) to the ground state ($\pi\pi^\star \rightarrow \mathbf{S}_0$)

Early works assigned a unique time constant of ~600-700 fs to internal conversion [Peo01, Pec01]. Improvements in the temporal resolution have meanwhile revealed an additional time constant as short as ~100 fs in all pyrimidines [Buc15, Can05, Bri19, Che13, Pep17, Gus06, Har07, Oni02] suggesting that ICs may occur much faster than originally anticipated. In support of the $\pi\pi^\star \rightarrow S_0$ direct IC hypothesis, theoretical studies have disclosed a conical intersection (CI) seam characterized by ring-puckered structures with hydrogen (Ura, Cyd), methyl (5mUra), amino (Cyd) and/or carbonyl out-of-plane bent groups [Buc15, Nak13, Har08, Pep18].

A further $\pi\pi^\star \rightarrow S_0$ decay channel has been hypothesized in the case of Ura and Thy [Pep17, Ric14, Nac11, Bar10, Gha18, Bar13]: a ring opening mechanism characterized by the N_1-C_2 bond breaking, which could potentially lead to photoproducts formation.

Recent molecular dynamics simulation of 5mUrd/5mUra in water support the hypothesis that the entire population follows the $\pi\pi^\star \rightarrow S_0$ decay path (including both ring puckering and carbonyl bending mechanisms) in the sub-picosecond time range [Buc15, Nak13, Har08], assigning the few ps lifetime to the dissipation of the excess of energy of the electronic ground state to the solvent, by vibrational cooling. The $n\pi^\star$ state is found to be strongly destabilized and conversely does not play any role in the decay path, in contrast to what happens in gas phase. The hypothesis is further supported by recent liquid jet time-resolved photoelectron spectroscopy in combination with XMS-CASPT2//CASSCF/AMBER calculations, providing a time constant for the $\pi\pi^\star$ decay of ca. 400 fs [Eri19].

1.2 IC to an intermediate dark state ($\pi\pi^\star \rightarrow n\pi^\star$)

Although the involvement of a dark state of $n\pi^\star$ nature is very probable for pyrimidines in gas phase, as confirmed both in experimental [He03, He04, Yu16, Wol19] and theoretical [Imp16, Sto16, Per06, Mai17, Wol19, Par19] studies, the role of dark states in condensed phase is still controversial. According to the hypothesis of the involvement of the $n\pi^\star$ state in solution, the shortest time constant is to be assigned to the CI mediated decay to the intermediate dark state, with lifetimes ranging from few hundred femtoseconds to few hundred picoseconds [Kob12, Pro16, Xue16, Coh04, Bri19, Cer18, Liu18].

Regarding the deactivation of the $^1n\pi^\star$ state, currently three mechanisms are hypothesized:

1.2.1 Direct IC to the ground state ($n\pi^\star \rightarrow \mathbf{S}_0$)

Occurs on sub-ps to few ps time scale through an energetically higher lying $^1n\pi^\star/\mathrm{S}_0$ CI seam [Bri19,Lis18].

1.2.2 Decay to a triplet state ($n\pi^\star \rightarrow \mathbf{T}_1 \rightarrow \mathbf{S}_0$)

Occurs on a sub-ps to ps time scale, facilitated by the energetic proximity of states of different multiplicity in the vicinity of the $^1n\pi^\star$ minimum and the large spin orbit couplings (mainly with the $^3\pi\pi^\star$), in agreement with El-Sayed's rule [Kwo08, Wol19, McN97]. The triplet itself is proposed to decay to the ground state on a ps to ns time scale [Pil18]. Support for the formation of the triplet state has been provided by dynamics calculations for Ura. [Ser07, Ric14, Gon10]

1.2.3 Re-population of the $\pi\pi^\star$ state ($n\pi^\star \rightarrow \pi\pi^\star \rightarrow \mathbf{S}_0$)

Occurs on a tens to hundreds of ps time scale. Support for this mechanism is provided by a recent computational work [Con18] on stacked thymidines which shows that the $\pi\pi^\star \leftrightarrow n\pi^\star$ exchange could involve a molecular distortion very sensitive to the sugar substitution, thus potentially rationalizing the longer lifetimes observed in nucleosides, compared to the nucleobases.

1.3 ISC from the bright $\pi\pi^\star$ to an intermediate triplet $^3n\pi^\star$ state ($\pi\pi^\star \rightarrow {}^3n\pi^\star$)

This decay process has been documented recently for the solvated uridine monophosphate [Bri19] on the basis of femtosecond broadband TA spectroscopy experiments. A trifurcation process from the bright $\pi\pi^\star$ state is proposed, where the system decays within 200 fs simultaneously to the ground state, to the singlet $n\pi^\star$ and to the triplet $^3n\pi^\star$ state which subsequently funnels the population to the energetically lower lying $^3\pi\pi^\star$ triplet state, which survives on the ns time scale.

Supplementary Note 2: Theoretical background and protocols for acquisition of simulation parameters

2.1 Classical molecular dynamics

Classical molecular dynamics simulations for Urd and 5mUrd dissolved in water were performed with the Amber 12 suite of programs [Fer13,Cas12] utilizing the Parm99 force field and the all_nucleic02.lib libraries for RNAs. Periodic boundary conditions were applied to a periodic box of water molecules described by the TIP3P force field [Jor83]. The hydrogen-containing bonds were restrained by the SHAKE algorithm [Ryc77] while the water geometry was rigidized by the SETTLE scheme [Miy92]. Non-bonding and electrostatic interactions were evaluated with a cutoff of 9.0 Å making use of the particle mesh Ewald method for quantification of the long-range electrostatics. The whole system, including the nucleoside moiety, was equilibrated from 0 to 300°K for 1 ns while maintaining a constant volume and pressure (1 atm). A production run was then performed for 100 ns recording snapshots every 200 fs. Analysis of the geometrical changes along the trajectories is presented in [Pep17].

It is well known that in mono-nucleosides two predominant conformers, syn and anti, can be populated. A cluster analysis performed along the trajectories gives as most probable geometry a syn conformation that is stabilized by an intramolecular hydrogen bond between the CH_2OH group on the sugar and the C=O group in the nucleobase. To select the initial geometry for subsequent QM/MM refinement and generation of initial conditions we performed a cluster analysis based on a Root Mean Square (RMS) coordinate deviation analysis on the nucleoside moieties over all snapshots recorded along the dynamics run within a 1.5 Å difference using the MMTSB toolbox of Amber 12 [Fei01]. We obtained two clusters, denoted A and B, with syn/anti populations relating to 73/27 and 62/38 for Urd and 5mUrd, respectively. The snapshot with the geometrical parameters closest to the centroid (i.e., the mean position) of the structures obtained in cluster A, was selected for further QM/MM refinement.

2.2 QM/MM geometry refinement

QM/MM calculations were executed with the COBRAMM suite, developed in our group interfacing various QM codes with the MM software AMBER

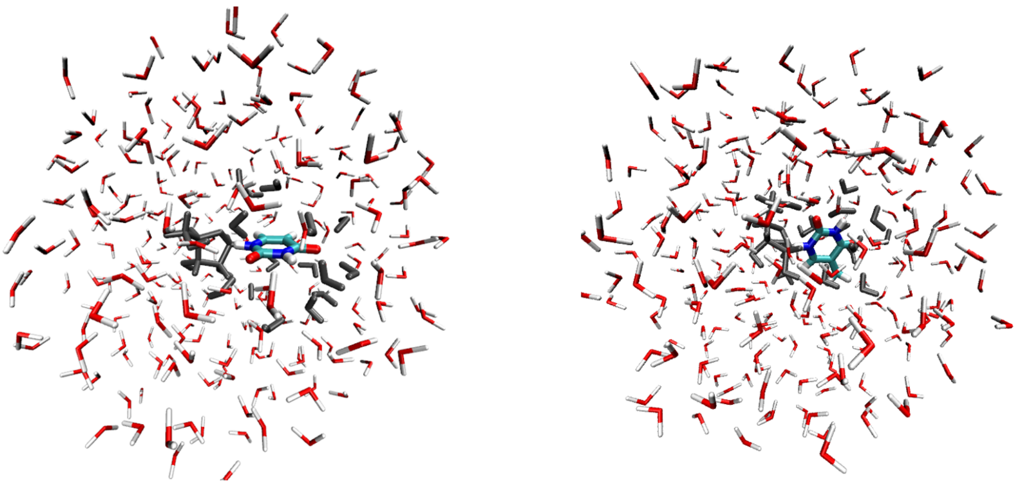

Supplementary Figure 1: High/Medium/Low layer partitioning of Urd (left) and 5mUrd (right) in the QM/MM refinement protocol. The High layer comprises the 5mUra, the Medium layer comprises the sugar and all waters in 5 Å distance from the center of mass of the nucleobase (gray color). The rest of waters are kept frozen in the Low layer.

[Alt07,Wei18]. A High Layer (HL) / Medium Layer (ML) / Low Layer (LL) partitioning was applied to a spherical droplet centered at the nucleoside with a radius of 12 Å (containing 260 waters), obtained from the cubic box (Figure 1). The HL (QM region) comprises the nucleobase. The water molecules in 5 Å distance from the center of mass of HL were included in the movable ML. The remaining water molecules were kept fixed in the LL. The ground state minimum was obtained at the Møller-Plesset second order perturbation theory (MP2) level as implemented in the Molcas 8 package [Aqu16] through its interface with COBRAMM. The ANO-L basis set was used [Wid90], adopting a valence double-ζ contraction (i.e. 3s2p1d for non-hydrogen and 2s1p for hydrogen atoms).

2.3 Wigner sampling

For each system 500 initial conditions were generated using the Wigner sampling technique based on a frequency calculation at the MP2 optimized structure through an interface with a stand-alone script, part of the quantum molecular dynamics program JADE [Du15], which considers temperature effects and mode-specific excitation. Consequently, sampling was performed at room temperature (300 K). High frequency modes, belonging to C-H and N-H stretchings, were excluded from the sampling. In the case of 5mUrd two

normal modes with low frequencies (< 100 cm^{-1}) involving methyl group twisting along the CH_3-C_5 σ-bond were excluded from the production sampling as it was ascertained through preliminary sampling that displacement along such modes breaks the harmonic approximation and distorts excessively the geometry. Each of the sampled geometries was subjected to a 10 ps long MM equilibration run in which the QM region was kept fixed through harmonic constraints. This strategy allows the mobile MM region to adapt to each sampled geometry, thereby removing the bias of the water arrangement of the initial snapshot. The water coordinates and velocities of the last snapshot of the MM equilibration run were then combined with the coordinates and velocities of the sampled nucleoside geometry to form a snapshot used to initiate the mixed quantum classical dynamics simulations.

2.4 Linear absorption spectroscopy

The electronic spectrum of two systems was computed at the CASSCF/CASPT2 level with the Molcas 8 software package through its interface with COBRAMM. For the active space size dependence tests the full valence-π active space comprising 10 electrons in 8 orbitals (i.e. CAS(10,8)) was extended by four and eight virtual π-orbitals, respectively. The restricted active space modification of the CASSCF protocol (i.e. RASSCF) allowing for up to four simultaneous excitations was used. In the following we will refer to these active spaces as $\mathrm{RAS}(4,5|0,0|4,7)$ and $\mathrm{RAS}(4,5|0,0|4,11)$[1]. The excited state computations were averaged over two and four states, respectively. Additionally, the CAS(10,8) and $\mathrm{RAS}(4,5|0,0|4,7)$ were augmented by the two lone pairs of the oxygens to disentangle the effect of the $n\pi^*$ transitions on the linear absorption (LA) spectra, thus giving CAS(14,10) and $\mathrm{RAS}(4,7|0,0|4,7)$. In these cases the excited state computations were averaged over six states. The CASPT2 computations were performed both in the single-state and multi-state flavor. All calculations were performed with the generally contracted basis set ANO-L adopting valence double-ζ contractions.

For each system the spectra were computed utilizing the transition energies and dipole moments computed at 500 snapshots generated via Wigner sampling according to the formula

[1]In the RASSCF method the molecular orbitals and electrons are divided into three active spaces: RAS1|RAS2|RAS3. We adopt the following notation: $(i,j|k,l|n,m)$ with i and j the maximal number of holes and occupied orbitals in RAS1, k and l the number of electrons and orbitals in RAS2, and n and m the number of simultaneous excitations and virtual orbitals in RAS3.

$$I(\omega) \propto \frac{1}{N_{traj}} \sum_{k}^{N_{traj}} \sum_{e} \mu_{\mathbf{eg}}^{\mathbf{2}}(\mathbf{R}_k) g(\Delta E_{eg}(\mathbf{R}_k) - \hbar\omega) \tag{1}$$

where k iterates over the number of realizations, $\mathbf{R}_k$ denotes the geometrical coordinates and f denotes a state from the manifold of excited states. The intensity is convoluted with a normalized line shape function $g(\Delta E_{eg}(\mathbf{R}_k) - \hbar\omega)$ peaked at the transition energies and broadened by a phenomenological constant σ:

$$g(\Delta E_{eg}(\mathbf{R}_k) - \hbar\omega) = \exp\left(-\frac{E^{(e)}(\mathbf{R}_k) - E^{(g)}(\mathbf{R}_k) - \hbar\omega)^2}{2\sigma^2}\right) \tag{2}$$

For σ we chose a value of 0.1 eV to match the experimental LA spectra. State-specific spectra were generated by grouping transitions according to the leading configuration of the ES wave function (i.e. n→L for $n\pi^*$ and H→L for $\pi\pi^*$). For the purpose of the discussion we use the terms configuration and state interchangeably.

2.5 Non-adiabatic mixed quantum-classical dynamics simulations

For each system, out of the 500 snapshots generated via Wigner sampling, 57 geometry realizations were selected for running mixed quantum classical molecular dynamics simulations with the condition that the transition energy of the lowest $\pi\pi^*$ state computed at the SS-CASPT2/SA-4-RASSCF$(4, 5|0, 0|4, 11)$ level falls under the envelope of the pump pulse, as this level is shown to give excellent agreement with experiment (see Figure 10e).

2.5.1 Technical details on the SS-CASPT2 production dynamics

Molecular dynamics simulations following Newton's equations of motion for the nuclei and utilizing hybrid QM/MM gradients were performed at the full-π SS-2-CASPT2/SA-2-CASSCF(10,8) level of theory for 500 fs in the case of Urd and for 1000 fs in the case of 5mUrd with a time step of 1.0 fs applying the Tully's fewest switches surface hopping algorithm [Tul90] with the Tully-Hammes-Schiffer (THS) scheme [Ham94] using the COBRAMM's parallel environment for computing numerical gradients. The state averaging covered the ground and the lowest $\pi\pi^*$ state (S_1). The movable (MM) water layer was extended (now comprising all water molecules within 5 Å from the

HL) to accommodate distortions of the aromatic ring such as puckering, as predicted by static calculations [Pep17].

Gradients were computed numerically through a two-point finite differentiation formula using the COBRAMM's parallel environment. To speed up the numerical computations of the SS-RASPT2 gradients we made use of the fact that the perturbative correction is applied independently on each root. Thus, while at the reference point all states are corrected at SS-RASPT2 level, during the computation of the gradient only the state that drives the nuclear dynamics (i.e. the photoactive state) was taken into account. Care was taken for possible state swapping at each displaced geometry in case of near-degeneracy at the RASSCF level by following the nature of the wave function. This was facilitated by computing the wave function overlap between reference and displaced geometries within the RASSI utility[Mal86] of Molcas. The stability of the simulations was monitored by COBRAMM. If

a) an element the numerical gradient exceeds a threshold (default 0.5)

b) the ratio of the lengths of the gradient vectors at two consecutive steps (i.e. $||g(t)||/||g(t-dt)||$) and, at the same time, the deviation of the total energy from its value at the previous step (i.e. $E_{tot}(t)-E_{tot}(t-dt)$) exceed certain thresholds (defaults 1.3 and 0.5 kcal/mol, respectively)

c) a single point computation fails to converge

the gradient is discarded and the propagation of the nuclei is repeated at the last stable geometry, first with a half step (0.5 fs) and, in case the gradient is discarded again, with a double step (2.0 fs).

Following the THS scheme the expression for the time-derivative coupling (TDC) between states i and j

$$\sigma_{ij} = \frac{dR}{dt} d_{ij} = \frac{dR}{dt} \left\langle \Psi_i(r,t) \middle| \frac{d}{dR} \Psi_j(r,t) \right\rangle = \underbrace{\left\langle \Psi_i(r,t) \middle| \frac{d}{dt} \Psi_j(r,t) \right\rangle}_{TDC} \quad (3)$$

is approximated to finite differences and the change in the electronic wave functions is resolved by computing overlap integrals between the adiabatic wave functions at different time steps. We generalized the TDC formulation

of Barbatti (Eq. 9 in ref. [Pit09]) to arbitrary time steps Δt:

$$\begin{aligned}\sigma_{ij} = \frac{1}{2\Delta\tau}\left(1 - \frac{0.5\Delta\tau + \Delta t}{0.5\Delta\tau + 0.5\Delta t}\right) & (\langle\Psi_i(t-\Delta t)|\Psi_j(t-\Delta t-\Delta\tau)\rangle - \\ & \langle\Psi_j(t-\Delta t)|\Psi_i(t-\Delta t-\Delta\tau)\rangle) + \\ \frac{1}{2\Delta t}\left(\frac{0.5\Delta\tau + \Delta t}{0.5\Delta\tau + 0.5\Delta t}\right) & (\langle\Psi_i(t)|\Psi_j(t-\Delta t)\rangle - \\ & \langle\Psi_j(t)|\Psi_i(t-\Delta t)\rangle)\end{aligned} \tag{4}$$

where Δt and $\Delta\tau$ are the time steps used to propagate the time-dependent Schrödinger equation between step $k-1$ and k, and between step $k-2$ and $k-1$, respectively. Note that eq. 4 simplifies to eq. 9 in ref. [Pit09] when $\Delta\tau = \Delta t$. The individual terms are obtained from wave function overlaps, which are computed at the RASSCF level (i.e. using the RASSCF wavefunctions) through the RASSI utility [Mal86] of Molcas and further scaled by the ratio of the SS-RASPT2 and RASSCF energy gaps $(E_j^{CASSCF} - E_i^{CASSCF})/(E_j^{SSPT2} - E_i^{SSPT2})$), which follows from the relation:

$$\begin{aligned}\sigma_{ij} = & \langle\Psi_i(t)^{SSPT2}|\frac{d}{dt}\Psi_j(t)^{SSPT2}\rangle = \frac{dR}{dt}\langle\Psi_i(t)^{SSPT2}|\frac{d}{dR}\Psi_j(t)^{SSPT2}\rangle = \\ & v \cdot d_{ij}^{SSPT2} = v \cdot \frac{\langle\Psi_i(t)^{SSPT2}|\nabla_R\hat{H}|\Psi_j(t)^{SSPT2}\rangle}{E_j^{SSPT2} - E_i^{SSPT2}} \approx \\ & v \cdot \frac{\langle\Psi_i(t)^{CASSCF}|\nabla_R\hat{H}|\Psi_j(t)^{CASSCF}\rangle}{E_j^{CASSCF} - E_i^{CASSCF}} \cdot \frac{E_j^{CASSCF} - E_i^{CASSCF}}{E_j^{SSPT2} - E_i^{SSPT2}} = \\ & v \cdot d_{ij}^{CASSCF} \cdot \frac{E_j^{CASSCF} - E_i^{CASSCF}}{E_j^{SSPT2} - E_i^{SSPT2}}\end{aligned} \tag{5}$$

In the above derivation following equality is assumed:

$$\langle\Psi_i(t)^{SSPT2}|\nabla_R\hat{H}|\Psi_j(t)^{SSPT2}\rangle \approx \langle\Psi_i(t)^{CASSCF}|\nabla_R\hat{H}|\Psi_j(t)^{CASSCF}\rangle \tag{6}$$

Effectively, when the SS-RASPT2 correction to the SA-CASSCF energies decreases the energy gap between a pair of states, the value of the time-derivative coupling is increased uniformly and linearly. In addition, a wave function following algorithm allows to identify state swapping at the SS-CASPT2 level. The THS scheme allows to work with relatively large time steps (such as 1.0 fs) and performs better in the region of $\pi\pi^*/n\pi^*$ near-degeneracy compared to the original Tully scheme based on non-adiabatic coupling vectors.

Tully's fewest switches surface hopping algorithm is known to suffer from

artificial coherence effects. A decoherence correction, originally proposed by Truhlar and co-workers [Zhu05] and realized by Persico et al. [Gra07] is used in the present case as a countermeasure. Kinetic energy scaling (for total energy conservation) after a hopping event is performed along the velocity vector.

A crucial step in the execution of the simulations is thus to make sure that the oxygen lone pairs do not enter the active space. This was realized in the following way. At the first step of each trajectory it was assured that the active space contains only π-orbitals. At every following step t a reference CASSCF calculation was run, re-optimizing the molecular orbitals of the previous step $t - \Delta t$ without any restrictions. Subsequently, the nature of each state i was tested by computing the norm of its overlap with the states at the previous time step following the formula $\left|\left|i\right|\right| = \sum_j \left\langle i(t) \middle| j(t - \Delta t) \right\rangle$ with elements obtained from the overlap matrix $S(t - \Delta t \rightarrow t)$. If the value for the overlap is above a certain threshold (75 %), meaning that the character of the state is essentially conserved, we proceed with the calculation of the CASPT2 energies and gradients. Instead, if the value for the overlap is below the threshold, we assume that the active space was contaminated with a lone pair. This generally happens when the $n\pi^*$ state is considerably stabilized at the CASSCF level (as in the case of elongation of the carbonyl bonds) so that rotating a lone pair at the place of the completely bonding π-orbital would lower the total state-averaged energy of the system. In such case the CASSCF calculation is repeated once again (using the molecular orbitals of the previous step), this time preventing orbital rotations between the lone pairs and the remaining orbitals. CASPT2 energies and gradients at this time step are then all computed in the same manner. In the subsequent time step $t + \Delta t$ all restrictions are released and the overlap norms $\left|\left|i\right|\right|$ are computed again. Benchmarking showed that "freezing" the lone pairs for a few steps has practically no effect on the outcome of the dynamics. Moreover, it was found that simulations with continuously "frozen" lone pairs (i.e. forced in a sup-symmetry block) begin to deviate from simulations with "free" lone pairs after about 50 fs.

2.5.2 Technical details on the MS- and XMS-CASPT2 benchmarking dynamics on Uridine

The computational level selected for the production runs has one major shortcomings, it neglects the involvement of other electronic states, e.g. the close lying dark state of $n\pi^*$ nature believed to be involved to some extent in the internal conversion. In an attempt to address this shortcoming we per-

formed benchmark studies in which we included the two oxygen lone pairs in the active space and/or higher roots of $\pi\pi^*$ and $n\pi^*$ nature in the state-averaging. The tests showed that pronounced $n\pi^*/\pi\pi^*$ wavefunction mixing at the CASSCF level makes dynamics simulations unstable both at single state (SS) and at the multi state (MS) levels, the cause lying in the numerical calculation of the energy gradients, exhibiting non-physically large energy changes (and, hence, forces) for infinitesimal geometry displacements in regions of strong wavefunction mixing at the CASSCF level. Despite being able to substantially improve the stability of the simulations with the aforementioned sanity checks, e.g. through doubling the time-step in order to over-step the regions of strong mixing (see Supplementary Note 2.5) it was impossible to maintain energy conservation over the period of several hundred femtoseconds as often strong wavefunction mixing was encountered in extended regions of the potential energy surface, that cannot be overcome even by tripling the size of the time step. Still, we were able to run stable MS-CASPT2 dynamics with a π-only active space and three $\pi\pi^*$ roots in the state-averaging giving us the possibility to assess the involvement of higher lying $\pi\pi^*$ states in the internal conversion.

The recently implemented extended multi state (XMS) flavor of CASPT2[Gra11,Shi11], i.e. a multi-state variant which, unlike MS-CASPT2, is invariant under unitary transformations of the reference wave functions, provides smooth potential energy surfaces even in the presence of strongly mixing reference wave functions and is thus able to maintain the energy conservation even with $n\pi^*$ states explicitly considered. Thus, the XMS-CASPT2 dynamics can be used to validate how adequate is the omission of the $n\pi^*$ states in the production run.

Both multi-state flavors of CASPT2 resolve one further shortcoming of SS-CASPT2, namely that it does not describe correctly the topology of crossing seam predicting a (N-1)-dimensionality instead of the correct (N-2) [[**?**]], a flaw with a potential impact on the excited state lifetime. Thus, comparison between the single- and multi-state flavors of CASPT2 would allow to assess the reliability of the lifetimes obtained from the simulations.

In a nutshell, we performed benchmarking dynamics on Uridine at:

- the full-π MS-4-CASPT2/SA-4-CASSCF(10,8) level with a state averaging covering the ground state and the lowest three $\pi\pi^*$ roots on a subset of 50 trajectories from the production run

- full-π+2n(O) XMS-9-CASPT2/SA-9-CASSCF(14,10) level with a state averaging covering the lowest nine roots - the ground state, four $\pi\pi^*$ and four $n\pi^*$ states - on a subset of 25 trajectories from the production run.

The simulations were run with the same computational protocol used for the SS-CASPT2 production run for up to 500 fs. Contrary to the SS-CASPT2 protocol, it was not necessary to rescale the time-derivative couplings as perturbatively modified wave functions obtained after diagonalizing the MS- and XMS-Hamiltonians were used to compute the wave function overlaps that enter in eq. 4. The comparison of the three computational levels is discussed in Supplementary Note 3.4

We note that both MS- and XMS-CASPT2 simulations are considerably more expensive compared to SS-CASPT2 due to the requirement of computing the entire Hamiltonian (whose number of elements grows quadratically with the number of states) at each displaced geometry during evaluation of the numerical gradients. Thus, we could not collect a sufficient number of trajectories to analyze the statistics of the $n\pi^*$ state involvement in the deactivation mechanism. For this reason its role was quantified through an ad-hoc algorithm outlined in Supplementary Note 2.6.

2.6 How to estimate the $n\pi^*$ involvement

For the reasons outlined in the previous section we could not obtain energy conservation at SS-CASPT2 level used for the production dynamics during the molecular dynamics simulation when the oxygen lone pairs were included in the active space. Therefore, these simulations cannot directly shed light on the involvement of the $n\pi^*$ state in the ultrafast (sub-ps) non-adiabatic dynamics. However, it is well known that the lowest $\pi\pi^*$ and $n\pi^*$ states are energetically close and, as elucidated in the main text, there is still a controversy in the literature regarding the importance of the $n\pi^*$ state for trapping excited state population. In order to estimate the percentage of trajectories likely to undergo non-adiabatic population transfer to another excited state before decaying to the ground state we:

- analyzed the extent of $\pi\pi^*/n\pi^*$ mixing in the FC region relying on the MS-6-RASPT2/SA-6-RASSCF$(4,5|0,0|4,7)$ calculations, the level which agrees best with the experimental LA spectrum (Figure 10h); to this aim, we computed the overlap of the electronic states at the equilibrium and at each of the realizations used to initiate the molecular dynamics;

- computed the energies of further four excited states above the lowest $\pi\pi^*$ state at the MS-6-RASPT2/SA-6-RASSCF$(4,5|0,0|4,7)$ level of theory (two $n\pi^*$ and two $\pi\pi^*$ excited states) along the trajectories run at the SS-2-CASPT2/SA-2-CASSCF(10,8) level and analyzed the evolution of the energy gap between the lowest $\pi\pi^*$ state and the higher lying excited states in a diabatic representation;

- performed ad-hoc non-adiabatic Tully fewest switches surface hopping dynamics simulation (10 repetitions for 50 trajectories for Urd and 5mUrd each) in a diabatic basis to resolve the percentage of trajectories prone to a non-adiabatic population transfer to another excited state;

Below we outline the details of the diabatization procedure and of the non-adiabatic Tully fewest switches surface hopping dynamics in the dibatic picture. In a closed system diabatic ϕ^d and adiabatic ϕ^a states are connected via a transformation

$$|\phi^d\rangle = |\phi^a\rangle\mathbb{U} \tag{7}$$

diabatic states are defined as the combination of the adiabatic states that resemble as much as possible the reference states. At the beginning of the simulation ($t = 0$), it is assumed that the diabatic and adiabatic states coin-

cide[2]. At every time step of the simulation the (local) transformation matrix $\mathbb{U}(t-\Delta t\to t)$ is constructed via Löwdin orthogonalization of the overlap matrix $\mathbb{S}(t-\Delta t\to t)=\big\langle \phi^a(t-\Delta t)\big|\phi^a(t)\big\rangle$. In particular, we calculate the inverse of the overlap matrix $\mathbb{S}^{-1}(t-\Delta t\to t)$. The relation

$$\begin{aligned}\mathbf{1}=\mathbb{S}(t-\Delta t\to t)\mathbb{S}^{-1}(t-\Delta t\to t)=\big\langle \phi^a(t-\Delta t)\big|\phi^a(t)\big\rangle\mathbb{S}^{-1}(t-\Delta t\to t)=\\=\big\langle \phi^a(t-\Delta t)\big|\phi^d(t)\big\rangle\end{aligned} \tag{8}$$

demonstrates that the inverse of the overlap matrix rotates the adiabatic basis at time step t to ensure maximal overlap with the wavefunctions at the previous time step $t-\Delta t$. Thus, following the definition of the adiabatic-to-diabatic transformation, $\mathbb{S}^{-1}(t-\Delta t\to t)$ equals the local transformation matrix $\mathbb{U}(t-\Delta t\to t)$. The transformation matrix $\mathbb{U}(0\to t)$ connecting the adiabatic states at time step t to the references ones at the beginning of the simulation is computed by propagating the local transformation matrices.

$$\mathbb{U}(0\to t)=\mathbb{U}(0\to\Delta t)\mathbb{U}(\Delta t\to 2\Delta t)\mathbb{U}(2\Delta t\to 3\Delta t)\ldots \tag{9}$$

Thereby, in order to accurately perform the propagation one must keep track of relative signs of orbitals. In practice this is done by monitoring the sign of the diagonal elements of the local overlap matrices $\mathbb{S}(t-\Delta t\to t)$. Finally, the adiabatic-to-diabatic transformation matrix $\mathbb{U}(0\to t)$ is applied to the (diagonal) adiabatic Hamiltonian to transform it into a diabatic basis

$$\begin{aligned}\mathbb{H}^d(t)=\mathbb{U}^T(0\to t)\mathbb{H}^a(t)\mathbb{U}(0\to t)=\mathbb{U}^T(0\to t)\big\langle \phi^a(t)\big|\hat{H}\big|\phi^a(t)\big\rangle\mathbb{U}(0\to t)=\\=\big\langle \phi^d(t)\big|\hat{H}\big|\phi^d(t)\big\rangle\end{aligned} \tag{10}$$

The diabatic-to-adiabatic transformation holds for a closed electronic system, i.e. the states in the vector ϕ are separated from any higher lying states along the trajectory. In practice this condition is normally not fulfilled for any sub-space of states due to the high density and near degeneracy of excited states which leads to discontinuities due the occasional appearance of intruder states (i.e. not defined at the point of reference) and incomplete mapping to the states of reference. The incompleteness affects the local transformation matrix $\mathbb{U}(t-\Delta t\to t)$, an error which is then propagated in the computation of $\mathbb{U}(0\to t)$. To circumvent this obstacle we make use of the notion that

[2]This is not always the case. In fact, for uracil we observed in about 10% of the realizations a pronounced $\pi\pi^*-n\pi^*$ wavefunction mixing already in the FC point. These trajectories are excluded from the diabatization procedure

states which are energetically well separated are expected to mix negligibly[3], hence we introduce a threshold (10 kcal/mol) for the energy difference between adiabatic states i and j above which the two states are assumed to not mix. Thus, the matrix elements $U_{ij}(t-\Delta t \rightarrow t)$ and $U_{ji}(t-\Delta t \rightarrow t)$ of the local transformation matrix are set to 0.0. The same rationale is applied to the global transformation matrix $\mathbb{U}(0 \rightarrow t)$. Unlike the local transformation matrix, the global transformation matrix retains memory of the state order with respect to the FC point (point of reference). Thus, if at a time step t all energy gaps would be above the threshold, $\mathbb{U}(t-\Delta t \rightarrow t)$ would equal the identity matrix, whereas $\mathbb{U}(0 \rightarrow t)$ could contain 0 on the diagonal and ± 1 on the off-diagonal thus indicating state swapping[4]. Furthermore, we apply the diabatization to a sub-space of the lowest three roots (being ground state, $\pi\pi^*$ and $n\pi^*$ in the FC point), thereby assuring that the photoactive $\pi\pi^*$ state is described continuously and nearly completely in this sub-space[5]. In practice, along a given trajectory the third root could change nature (e.g. the $n\pi^*$ state occasionally gets energetically destabilized in favor of the second $\pi\pi^*$ state). We observe that this happens when the energy gap to the lowest $\pi\pi^*$ state is far above the selected threshold. Thus this state swapping has no effect on the population in the photoactive $\pi\pi^*$ state. Instead, we obtain a new set of reference wavefunctions (ground state, lowest $\pi\pi^*$ and second $\pi\pi^*$ states). Thus, the diabatization is not performed on a closed system of states along an entire trajectory. Instead, we obtain information about how often and how strongly the photoactive $\pi\pi^*$ state mixes with the closest lying excited state whose nature may vary. In the case of Urd the third root is almost exclusively a $n\pi^*$ state, however in 5mUrd we observe frequent swapping between $n\pi^*$ and the second $\pi\pi^*$ state.

Having the diabatic Hamiltonian at each time step we are in a position to perform ad-hoc non-adiabatic dynamics simulations in the diabatic basis. Thereby, we realize that the simulations performed at the SS-2-CASPT2/SA-2-CASSCF(10,8) level of theory (i.e. in the absence of lone pairs in the active space and thus of $n\pi^*$ states in the averaging procedure) can be regarded as performed in the diabatic picture, i.e. following the $\pi\pi^*$ state, thereby disregarding its mixing with any $n\pi^*$ or higher lying $\pi\pi^*$ states. We can thus

[3]Similar approximation is frequently adopted in non-adiabatic dynamics in the adiabatic representation in order to reduce the effort in computing non-adiabatic coupling elements.

[4]$\mathbb{U}(0 \rightarrow t)$ is related to the identity matrix by 2 x 2 rotations (with an angle $\phi = 90$ °) on on each pair of swapped states.

[5]We have assured that the photoactive $\pi\pi^*$ state never becomes higher than the third root in the calculations. Furthermore, we observe that the photoactive $\pi\pi^*$ state always mixes with only one other state at a time.

re-run the trajectories, re-using at each time step the velocities for the nuclei computed at the SS-2-CASPT2/SA-2-CASSCF(10,8) level, thereby propagating the electronic coefficients c_i using the diabatic Hamiltonian obtained with aforementioned adiabatic-to-diabatic transformation

$$i\hbar\frac{\partial c_i}{\partial t} = \sum_j H_{ij}^d(t)c_j. \tag{11}$$

Thereby, we adopt the approximation that the occasional mixing of the $\pi\pi^*$ state with close-by electronic states does not affect the dynamics. This approximation is supported by the empirical observation on both systems under investigation that state mixing is a rare (in time) a highly localized (in space) event. At every time step the hopping probability between diabatic states is calculated according to the formula

$$P_{i\to j} = \max\left[0, \frac{2\Delta t}{c_i^* c_i}\Im(c_j^* c_i)H_{ji}^d\right] \tag{12}$$

and a transition from surface i to surface j (i.e. a hopping event) is realized through the usual stochastic procedure. An estimate of the percentage of trajectories that depart from the $\pi\pi^*$ state before its decay to the ground state is finally obtained by re-running the set of trajectories multiple times and averaging over the number of hopping events. Thereby, it is assumed that the system does not return on the $\pi\pi^*$ surface once it hopped to another surface. Thus, the obtained estimate could be regarded as an upper limit for the population transfer to other excited states.

2.7 Simulation of transient absorption spectra

On the basis of the simulations transient absorption spectra are generated after computing the excited state electronic structure (energies E and transition dipole moments $\mu_{\mathbf{ij}}$) of Urd and 5mUrd at the SS-15-CASPT2/SA-15-CASSCF(10,8) level of theory at every time step. In practice, at each delay time t_d between pump and probe, the transient spectrum is calculated at the coordinates as a superposition of stimulated emission to the ground state and photoinduced absorption to higher electronic states assuming simple vertical transitions.

$$\begin{aligned} I(\omega, t_d) \propto \frac{1}{N_{traj}} \sum_k^{N_{traj}} \Bigg[&\sum_f \mu^{\mathbf{2}}_{\mathbf{fe}\,f\leftarrow e}(\mathbf{R}_k(t_d))\; g(\Delta E_{fe}(\mathbf{R}_k(t_d)) - \hbar\omega) \\ &- \mu^{\mathbf{2}}_{\mathbf{eg}\,e\leftarrow g}(\mathbf{R}_k(t_d))\; g(\Delta E_{eg}(\mathbf{R}_k(t_d)) - \hbar\omega) \Bigg] \end{aligned} \tag{13}$$

where k iterates over the number of trajectories, $\mathbf{R}_k(t_d)$ denotes the geometrical coordinates at time t_d, e denotes the state which drives the semi-classical trajectory (i.e. either S_1 or the ground state) and f denotes a state from the manifold of excited states accessible with the probe pulse. The intensity is convoluted with a normalized line shape function $g(\Delta E_{ij}(\mathbf{R}_k(t_d)) - \hbar\omega)$ peaked at the transition energies and broadened by a phenomenological constant σ:

$$g(\Delta E_{ij}(\mathbf{R}_k(t_d)) - \hbar\omega) = \exp\left(-\frac{E^{(i)}(\mathbf{R}_k(t_d)) - E^{(j)}(\mathbf{R}_k(t_d)) - \hbar\omega)^2}{2\sigma^2}\right) \quad (14)$$

For σ we chose a value of 0.15 eV to remove statistical noise. The final spectrum has been uniformly blue-shifted by 0.3 eV resulting in a better match with the experiment. This correction is justified by the analogous red-shift observed in the LA spectra computed with CAS(10,8) with respect to the experiment (see Supplementary Note 3.2 for details).

Polarization control was incorporated by introducing a weighting factor $c(\mu_\alpha, \mu_\beta, \mathbf{E}_a, \mathbf{E}_b)$ to each contribution in the inner sum in eq. 13, functions of the angles between the incident electric fields $\mathbf{E}_a$ (pump) and $\mathbf{E}_b$ (probe) and between the coupled transition dipole moments μ_α and μ_β of the system.

$$\begin{aligned} c(\mu_\alpha, \mu_\beta, \mathbf{E}_a, \mathbf{E}_b) &= \left\langle (\mu_\alpha \cdot \mathbf{E}_a)^2 (\mu_\beta \cdot \mathbf{E}_b)^2 \right\rangle = \\ &= \frac{1}{15}\left[2 - \cos^2\Theta_{\mathbf{E}_a,\mathbf{E}_b} + \cos^2\Theta_{\mu_\alpha,\mu_\beta}(3\cos^2\Theta_{\mathbf{E}_a,\mathbf{E}_b} - 1)\right] \end{aligned} \quad (15)$$

Eq. 15 results from the general equation derived by Hochstrasser for four pulses and four interacting dipoles[Hoch01]. Thereby, $\mu_\alpha = \mu_{eg}(\mathbf{R}_k(0))$ is the transition dipole moment of the S_1 (bright state) at time $t_d = 0$ fs which interacts with the pump pulse, whereas $\mu_\beta = \mu_{fe}(\mathbf{R}_k(t_d))$ or $\mu_\beta = \mu_{eg}(\mathbf{R}_k(t_d))$ denotes the transition dipole moments of the states coupled to the photoactive states via the probe pulse at time t_d. For parallel arrangement of the pump and probe pulse ($\Theta_{\mathbf{E}_a,\mathbf{E}_b} = 0°$) eq. 15 simplifies to:

$$c(\mu_\alpha, \mu_\beta) = \frac{1}{15}\left[1 + 2\cos^2\Theta_{\mu_\alpha,\mu_\beta}\right] \quad (16)$$

whereas for orthogonal arrangement ($\Theta_{\mathbf{E}_a,\mathbf{E}_b} = 90°$) eq. 15 reads

$$c(\mu_\alpha, \mu_\beta) = \frac{1}{15}\left[2 - \cos^2\Theta_{\mu_\alpha,\mu_\beta}\right] \quad (17)$$

At the so called "magic angle" set up (pump and probe have an angle of 54.7°) the signal intensity becomes independent of the angle between the

dipole moments (the third term in eq. 15 vanishes as $3\cos^2(54.7°) - 1 = 0$). Thus, the intensity depends only on the magnitude of the dipole moments.

Finite pulse duration is accounted for ad-hoc by convolution of the transient absorption signal $I(\omega, t_d)$ with a Gaussian function in the time domain

$$I(\omega, t_d) = \int_{-\infty}^{+\infty} d\tau \ I(\omega, \tau) \ \exp\left(-\frac{(\tau - t_d)^2}{2\sigma^2}\right) \tag{18}$$

A standard deviation $\sigma = 11 \ fs$ was used (matching the experimental full-width-half-maximum of 25 fs).

Supplementary Note 3: Simulation results

3.1 Wigner sampling analysis

Below we present an analysis of the Wigner sampling regarding potential and kinetic energy redistribution, number of movable water molecules, temperature effects and several relevant coordinates.

3.1.1 Uridine (Urd)

Figure 2,a shows a representative snapshot of the Urd sampling with corresponding High/Medium/Low layer partitioning used for the mixed quantum-classical molecular dynamics simulations with waters within 5 Å distance to atoms of the uracil nucleobase (High Layer) being added to the movable Medium layer. On average 45 water molecules are included in the Medium layer.

The kinetic energy in the vibrational ground state of Urd is 3.12 eV at 0 K when all modes are considered in the sampling, whereas the kinetic energy reduces to 1.91 eV when only the lowest 69 modes are considered. The sampling at 0 K correctly reproduces this value (see Figure 3a) with the kinetic energy computed from the sampled velocities according to the formula:

$$E_{\text{kin}} = \frac{mv^2}{2}. \tag{19}$$

Thereby, 0.75 eV of the kinetic energy is accumulated by the uracil nucleobase (see Figure 3b). Performing the same sampling at room temperature (300 K) increases the average kinetic and potential energies by ca. 0.18 eV for Urd and 0.07 for uracil (see Figure 3a,b).

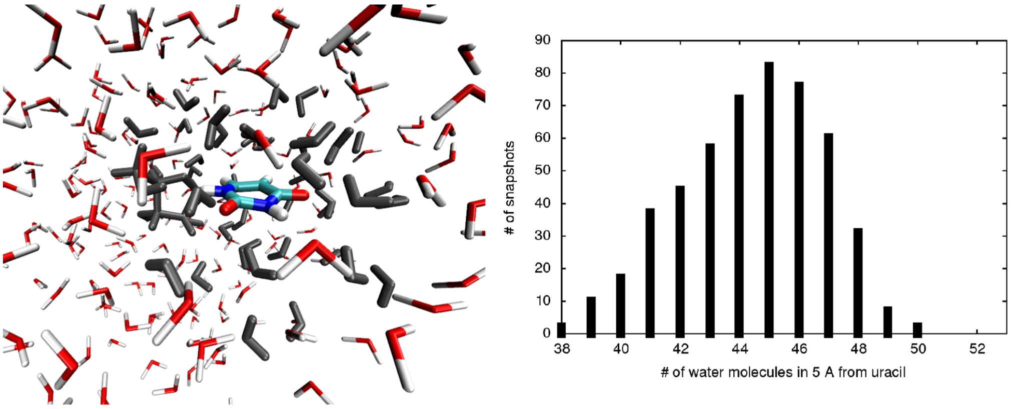

Supplementary Figure 2: Left: High/Medium/Low layer partitioning used for MD simulations; waters in 5 Å distance to Uracil (High Layer) added to the movable layer; Right: distribution of water molecules in all 500 snapshots generated in the Wigner sampling

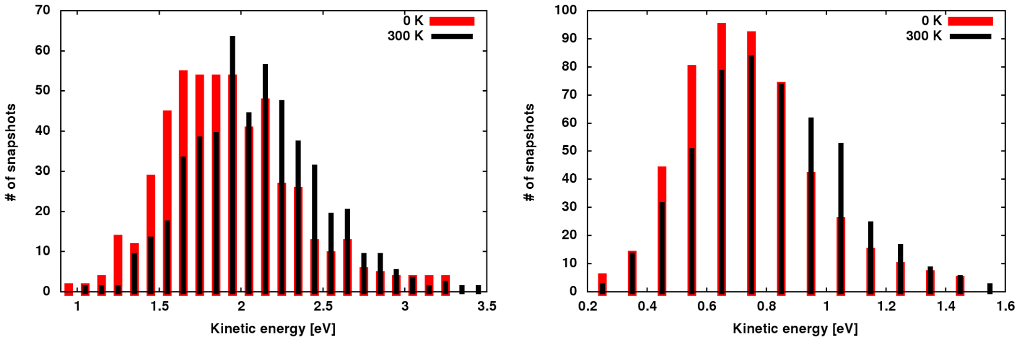

Supplementary Figure 3: Distribution of kinetic energies in Urd (left) and Uracil (right) for 0K and 300K

At 300 K the average N_1-C_2 bond length is 1.38 Å (see Figure 4,a) with few snapshots exhibiting elongation of up to 1.50 Å. The room temperature sampling does not lead to a more pronounced bond elongation compared to the sampling at 0 K. The distribution of dihedrals is Gaussian around the average values of 180 °(H_{10}-C_6-C_5-C_4 and H_9-C_5-C_6-N_1) / 0 °(H_{10}-C_6-C_5-H_9) (see Figure 4,b solid lines). The H_{10}-C_6-C_5-C_4 and H_9-C_5-C_6-N_1 dihedrals show fluctuations of ±20 °around the equilibrium value. The H_{10}-C_6-C_5-H_9 dihedral is more flexible showing fluctuations of ±30 °around the equilibrium value. The snapshots selected for the MD simulations which fall within the envelope of the pump pulse (277-266 nm) do not show any preferential geometrical distortion compared to the complete set of 500 snapshots (see Figure 4,b dotted lines).

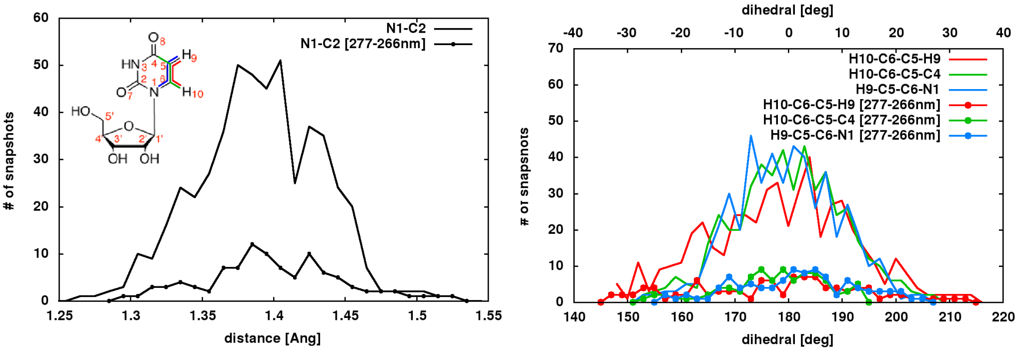

Supplementary Figure 4: Distribution of several characteristic coordinates in uracil in all 500 snapshots (solid lines) and in the window of pumping [277-266 nm] (dotted lines). Left: N1-C2 bond length; Right: H10-C6-C5-H9, H10-C6-C5-C4 and H9-C5-C6-N1 dihedrals

3.1.2 5-methyl-uridine (5mUrd)

Figure 5,a shows a representative snapshot of the 5mUrd sampling with corresponding High/Medium/Low layer partitioning used for the mixed quantum-classical molecular dynamics simulations with waters within 5 Å distance to atoms of the 5mUra nucleobase (High Layer) being added to the movable Medium layer. On average 46 water molecules are included in the Medium layer.

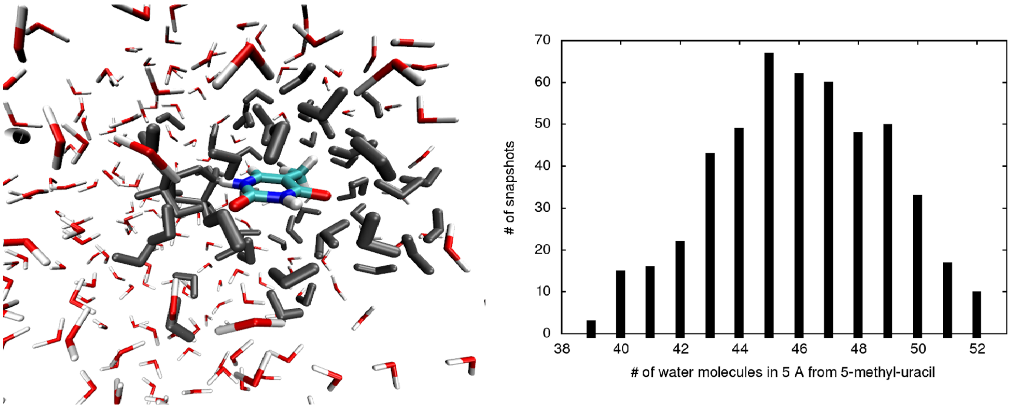

Supplementary Figure 5: Left: High/Medium/Low layer partitioning used for MD simulations; waters in 5 Å distance to Uracil (High Layer) added to the movable layer; Right: distribution of water molecules in all 500 snapshots generated in the Wigner sampling

The kinetic energy in the vibrational ground state of 5mUrd is 3.50 eV

at 0 K when all modes are considered in the sampling, whereas the kinetic energy reduces to 2.10 eV when only the lowest 74 modes are considered. The sampling at 0 K correctly reproduces this value (see Figure 6a). Thereby, 0.95 eV of the kinetic energy is accumulated by the 5mUra nucleobase (see Figure 6b). Performing the same sampling at room temperature (300 K) increases the average kinetic and potential energies by ca. 0.17 eV for 5mUrd and 0.07 for 5mUra (see Figure 6a,b).

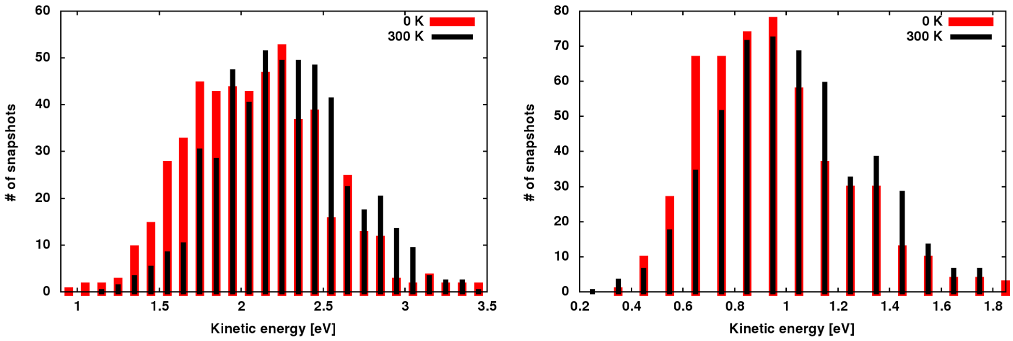

Supplementary Figure 6: Distribution of kinetic energies in 5mUrd (left) and 5mUra (right) for 0K and 300K

At 300 K the average N_1-C_2 bond length is 1.38 Å (see Figure 7,a) with few snapshots exhibiting elongation of up to 1.50 Å. The room temperature sampling does not lead to a more pronounced bond elongation compared to the sampling at 0 K. The distribution of dihedrals is Gaussian around the average values of 180 °(H_{10}-C_6-C_5-C_4 and CH_3-C_5-C_6-N_1) / 0 °(H_{10}-C_6-C_5-CH_3) (see Figure 7,b solid lines). The H_{10}-C_6-C_5-C_4 and CH_3-C_5-C_6-N_1 dihedrals show fluctuations of ±20 °and ±10 °around the equilibrium value, respectively. The H_{10}-C_6-C_5-CH_3 dihedral shows fluctuations of ±30 °around the equilibrium value. The snapshots selected for the MD simulations which fall within the envelope of the pump pulse (276-266 nm) do not show any preferential geometrical distortion compared to the complete set of 500 snapshots (see Figure 7b, dotted lines).

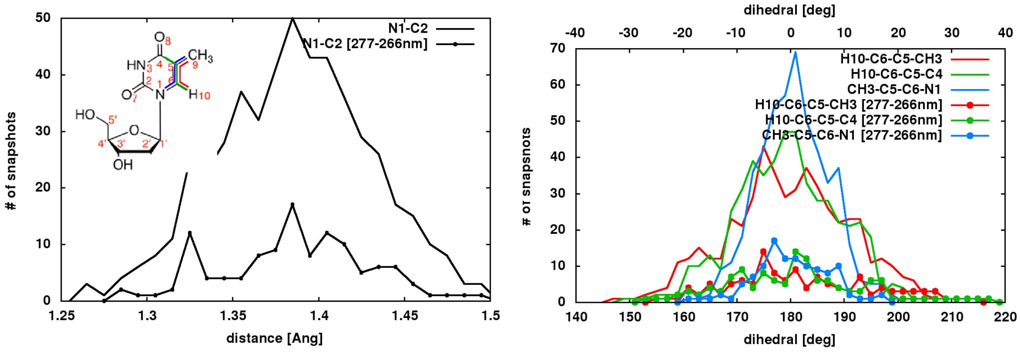

Supplementary Figure 7: Distribution of several characteristic coordinates in 5mUra in all 500 snapshots (solid lines) and in the window of pumping [277-266 nm] (dotted lines); Left: N1-C2 bond length; Right: H10-C6-C5-CH3, H10-C6-C5-C4 and H9-C5-C6-N1 dihedrals

3.1.3 Comparison

At room temperature 5mUrd has on average 0.18 eV more kinetic energy at disposal compared to Urd (2.27 vs. 2.09 eV) as seen by the shift of the distribution of kinetic energies (Figure 8,a). The 5mUra nucleobase has on average 0.20 eV more kinetic energetic at disposal compared to uracil (1.02 vs. 0.82 eV) (Figure 8,b).

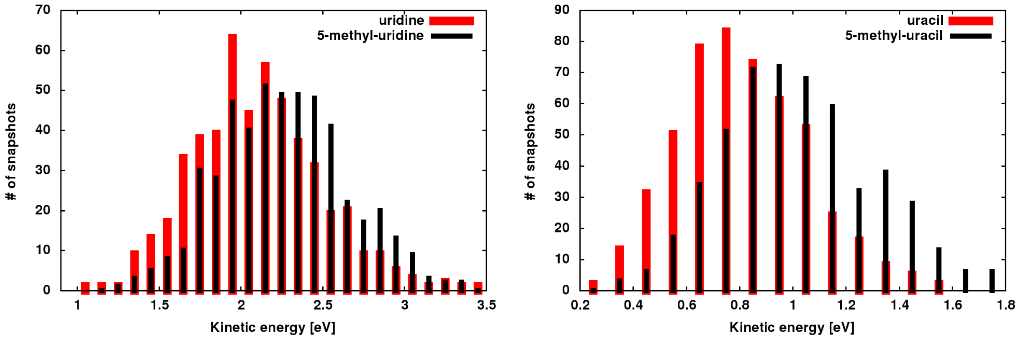

Supplementary Figure 8: Comparison between kinetic energies of Urd and 5mUrd (left) as well as between uracil and 5mUra (right)

The N_1-C_2 bond length exhibits an averaged value of 1.38 Å in both systems (see Figure 9,a). The major difference between uracil and 5mUra is the more pronounced flexibility of the H_9-C_5-C_6-N_1 dihedral compared to the CH_3-C_5-C_6-N_1 dihedral (see Figure 9,b blue solid vs. dotted line).

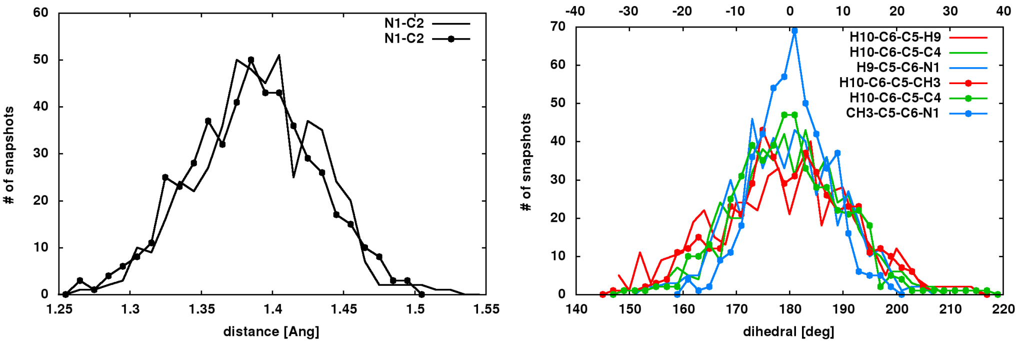

Supplementary Figure 9: Comparison of the distribution of several characteristic coordinates in Urd (solid lines) and 5mUrd (dotted lines); Left: N1-C2 bond length; Right: H10-C6-C5-H9/CH3, H10-C6-C5-C4 and H9-C5-C6-N1 dihedrals

3.2 Linear absorption spectra

In the following we present the LA spectra of Urd and 5mUrd computed at various levels of theory. In particular, we benchmarked active space, number of the states in the state-averaging procedure and RASPT2 flavor (single state vs. multi-state).

3.2.1 Uridine (Urd)

Figure 10a,b compares the LA spectrum of Urd computed at the SS-4-CASPT2/SA-4-CASSCF(10,8) level with active space comprising all π-orbitals of the system (black solid line) to the experimental spectrum (purple solid line). It is seen that both methods tend to red-shift the absorption energy of the $\pi\pi^*$ bands by ca. 0.30 eV ($\sim$ 20 nm at this wavelength). State-assignment (relying on the coefficients of the leading configuration state functions, CSFs) demonstrates that the lowest absorption band (peaking at 260 nm (280 nm) in the experiment (simulation)) is dominated by the H$\rightarrow$L transition (red solid line). Including the lone pairs of the oxygens in the active space, thus using CASSCF(14,10), allows to compute the contribution of the lowest $n\pi^*$ states (blue solid line in Figure 10c,d). As expected the GS$\rightarrow$ $n\pi^*$ transition is essentially dark and it does not affect significantly the LA spectrum. Increasing the active space with additional virtual orbitals leads to a much improved agreement with the experiment (Figure 10e-h). In the case of the π-only active space we could increase the active space in a RASSCF fashion by eight additional orbitals thus using a RASSCF$(4, 5|0, 0|4, 11)$ (Figure 10e-h), instead in the case of the active space augmented with lone pairs we could afford to add four virtual orbitals thus giving rise to a RASSCF$(4, 7|0, 0|4, 7)$. In both cases the $\pi\pi^*$ bands exhibit a blue-shift with respect to the smaller active spaces. Thereby, the multi-state treatment gives a slightly better agreement with experiment. As elucidated in the methodology sections we used these findings to define the criteria for selecting the snapshots for the mixed quantum-classical dynamics simulations, namely the snapshots which fall within the spectral envelope of the pump pulse (green solid line in Figure 10) at the SS-4-CASPT2/SA-4-CASSCF$(4, 5|0, 0|4, 11)$.

3.2.2 5-methyl-uridine (5mUrd)

Figure 11a,b compares the LA spectrum of 5mUrd computed at the SS-4-CASPT2/SA-4-CASSCF(10,8) level with active space comprising all π-orbitals of the system (black solid line) to the experimental spectrum (purple solid line). It is seen that both methods tend to red-shift the absorption energy of the $\pi\pi^*$ bands by ca. 0.35 eV ($\sim$ 25 nm at this wavelength).

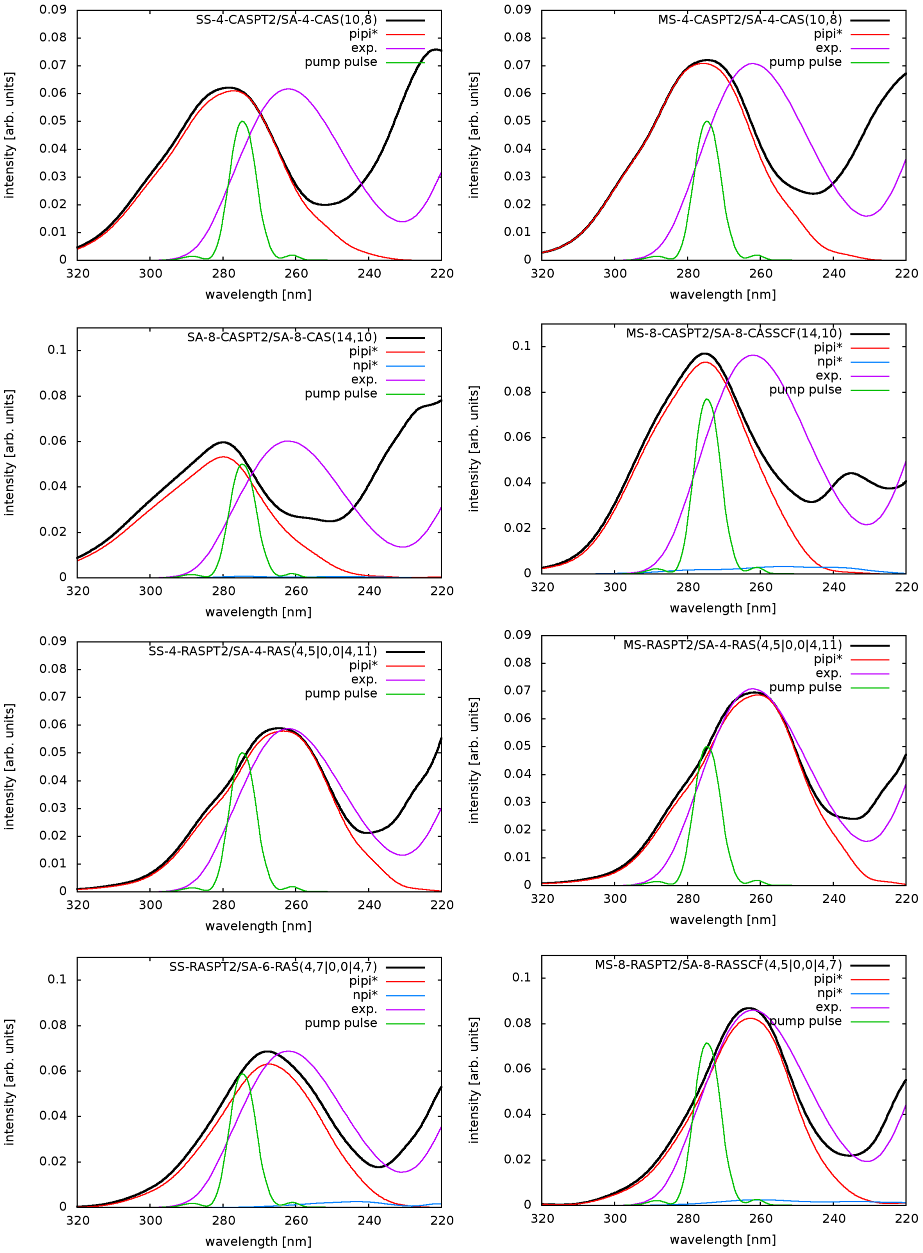

Supplementary Figure 10: Linear absorption spectra of Urd obtained with various levels of theory (specified in the legends of the individual panels). Contributions from the lowest $\pi\pi^*$ and $n\pi^*$ states are shown in red and blue colors, respectively. The experimental LA spectrum, as well as the pump pulse profile are also shown in magenta and green, respectively. The difference between the total spectrum (black) and the sum of the $\pi\pi^*$ and $n\pi^*$ contributions is due to wavefunction mixing with higher lying $\pi\pi^*$ states.

State-assignment (relying on the coefficients of the leading CSFs) demonstrates that the lowest absorption band (peaking at 266 nm (290 nm) in the experiment (simulation)) is dominated by the H$\rightarrow$L transition (red solid line). Including the lone pairs of the oxygens in the active space, thus using CASSCF(14,10), allows to compute the contribution of the lowest $n\pi^*$ states (blue solid line in Figure 11c,d). As in the case of Urd the GS$\rightarrow n\pi^*$ transition is essentially dark and it does not affect significantly the LA spectrum. Increasing the π-only active space with eight additional virtual orbitals, thus using RASSCF$(4,5|0,0|4,11)$ (Figure 11e-f), and with four additional virtual orbitals in the case of the active space augmented with lone pairs, thus using RASSCF$(4,7|0,0|4,7)$ (Figure 11g-h), leads to a much improved agreement with the experiment. As elucidated in the methodology sections we used these findings to define the criteria for selecting the snapshots for the mixed quantum-classical dynamics simulations, namely the snapshots which fall within the spectral envelope of the pump pulse (green solid line in Figure 11) at the SS-4-CASPT2/SA-4-CASSCF$(4,5|0,0|4,11)$.

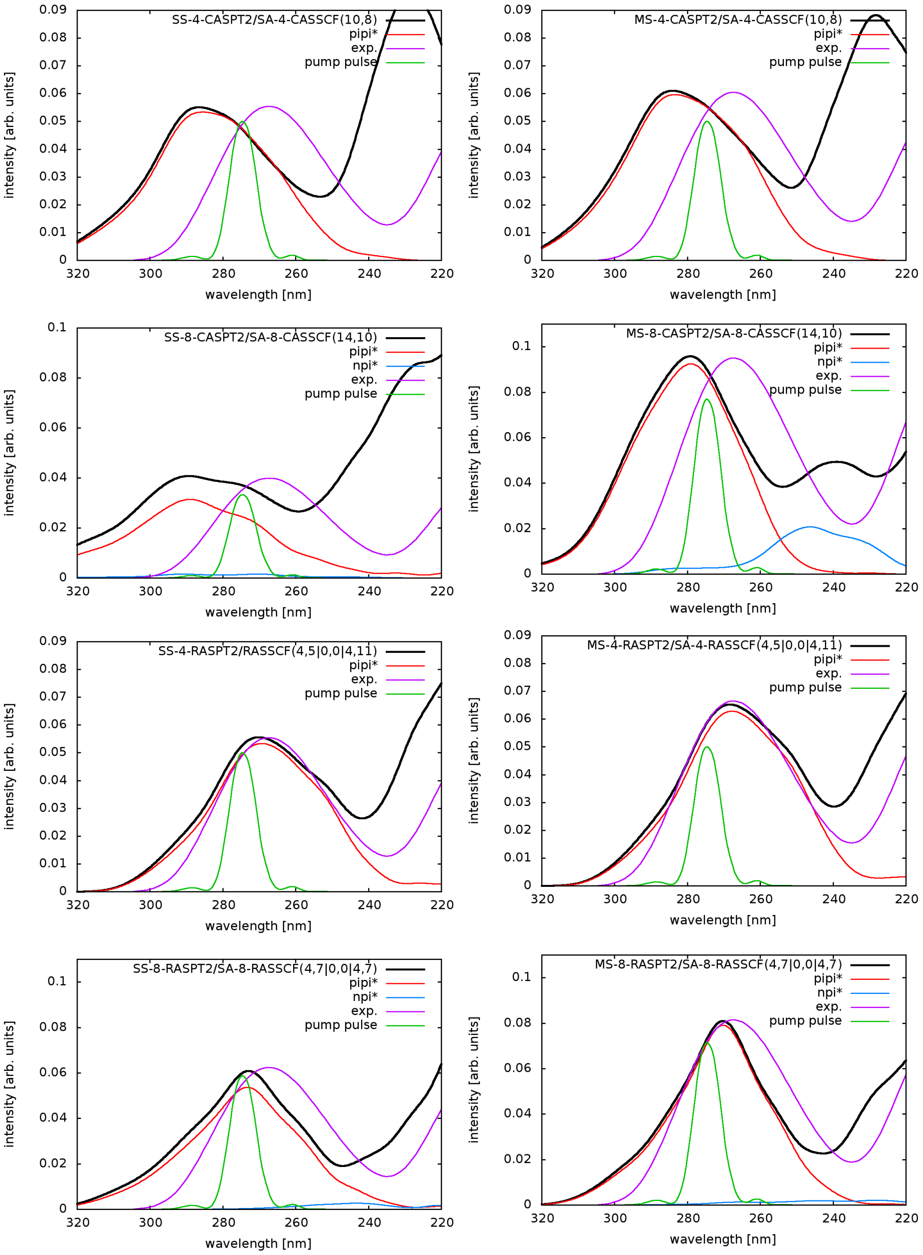

Supplementary Figure 11: Linear absorption spectra of 5mUrd obtained with various levels of theory (specified in the legends of the individual panels). Contributions from the lowest $\pi\pi^*$ and $n\pi^*$ states are shown in red and blue colors, respectively. The experimental LA spectrum, as well as the pump pulse profile are also shown in magenta and green, respectively. The difference between the total spectrum (black) and the sum of the $\pi\pi^*$ and $n\pi^*$ contributions is due to wavefunction mixing with higher lying $\pi\pi^*$ states.

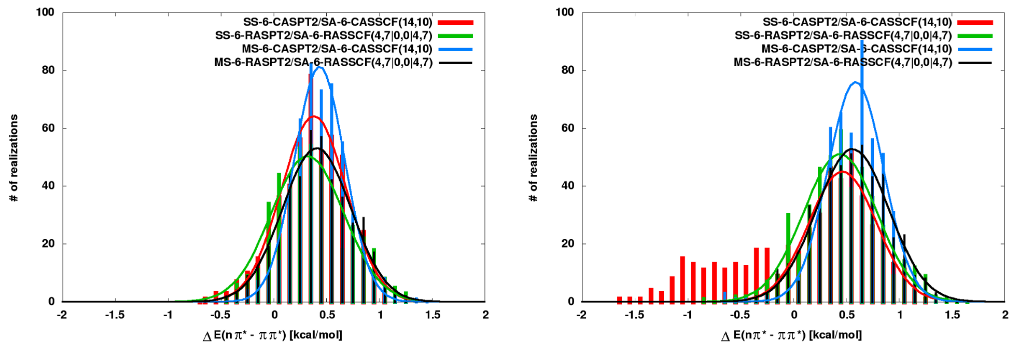

Supplementary Figure 12: $\pi\pi^* - n\pi^*$ energy gap distribution in Urd (left) and 5mUrd (right).

3.2.3 Comparison

Figure 12 compares the distributions of $n\pi^* - \pi\pi^*$ energy gaps in Urd (left) and 5mUrd (right) computed with the small CASSCF(14,10) (red and blue solid lines) and large CASSCF$(4,7|0,0|4,7)$ (green and black solid lines) at both single-state and multi-state RASPT2 level. A minor fraction of the samples (18% in the case of SS-CASPT2/CASSCF(14,10), 11% in the case of SS-RASPT2/RASSCF$(4,7|0,0|4,7)$) shows an inverted state order with the $n\pi^*$ being the lowest electronic state. At MS-CASPT2 level the number of snapshots with inverted state order is reduced with respect to the single state variant to 6% and 9%. Overall, it can be clearly seen that the majority of snapshots exhibit an $n\pi^*$ state well above the $\pi\pi^*$ state in the FC point slightly varying between 0.35-0.45 eV with the level of theory. Importantly, this demonstrates that the small active space seems to reproduce properly the $n\pi^*$ energy gap despite the overall red-shift of the transition energies seen in the corresponding spectra (Figure 10a,b)). With 0.50-0.60 eV the $n\pi^* - \pi\pi^*$ energy gap is even more pronounced in 5mUrd (Figure 12, right). A fraction of the samples (19% at SS-CASPT2/CASSCF(14,10)) shows an inverted state order. At MS-CASPT2 level the number of snapshots with inverted state order is reduced to 4%.

3.3 SS-CASPT2/SA-2-CASSCF(10,8) trajectory plots

In the following we plot the evolution of the individual representative trajectories computed for Urd and 5mUrd.

3.3.1 Uridine (Urd)

Figure 13 shows ten representative trajectories which were run for up to 500 fs. We observe a hop to the ground state in 53 of all 59 trajectories, with hops occurring as early as 50 fs (e.g. 13a,e), i.e. within two oscillations of the C-C double bond vibration. In total, we document 15 trajectories that hop on a sub-100 fs time scale, 21 trajectories which hop between 100-200 fs, 12 trajectories which hop between 200-300 fs and five trajectories which hop on a longer time scale (300-500 fs). Three trajectories do not hop on the time scale of the simulation (Figure 13i,j). The decay to the ground state occurs on all cases through H_9 out-of-plane puckering (Figure 25), whereas we observe a clear preference for down-puckering (41 trajectories, Figure 13a-d) with respect to up-puckering (12 trajectories, Figure 13e-h).

The CASPT2 dynamics with the full valence-π active space are overall stable, showing a tolerable increase of the total energy over the course of the simulation. There are several factors that play a role in this issue. Apart from the 1.0 fs time step, which is insufficient to describe fast stretching dynamics or the large amplitude dynamics of the "hot" ground state, the active space size also plays a significant role. Increasing the active space by two extra-valence orbitals reduces substantially the deviation of the total energy, in particular in the region of the crossing (Figure 14). Hence, the CAS(10,10) trajectories were utilized in the stochastic analysis and spectra simulations instead of their CAS(10,8) counterpart. The improved accuracy comes at the price of an increased cost in the computation, therefore only trajectories which show total energy deviation above 10 kcal/mol on the excited state (8 in Urd) were repeated with the larger active space. We note that the two sets of trajectories, SS-CASPT2/SA-2-CASSCF(10,8) (red) and SS-CASPT2/SA-2-CASSCF(10,10) (black) in Figure 14, behave qualitatively similarly on a short time scale (up to 300-400 fs). As a consequence the photophysics of Urd is not affected.

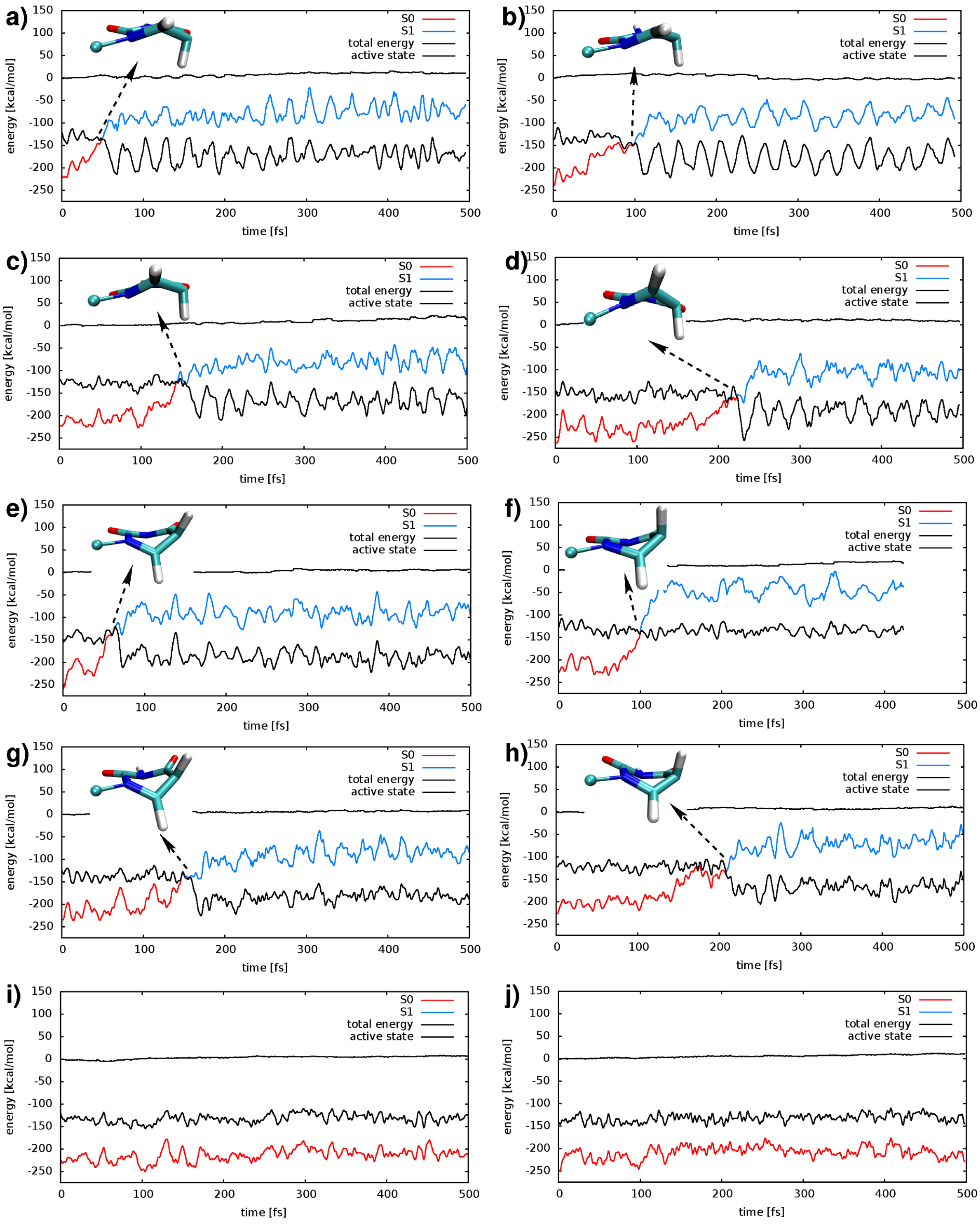

Supplementary Figure 13: a)-j): Ten representative trajectories for Urd

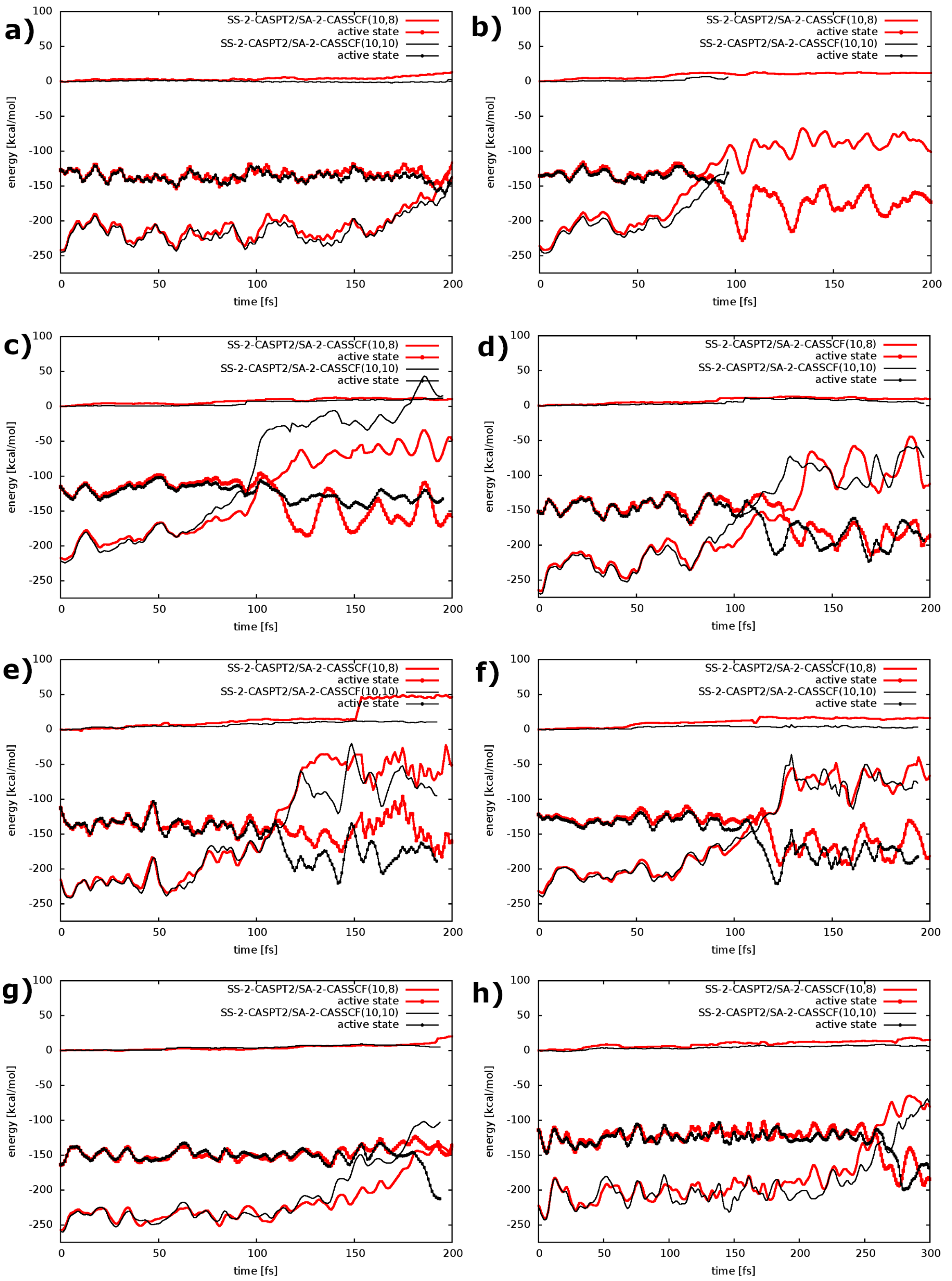

Supplementary Figure 14: a)-h): Comparison between eight representative trajectories computed at the SS-2-CASPT2/SA-2-CAS(10,8) (red) and at the SS-2-CASPT2/SA-2-CAS(10,10) (black) levels of the theory in Urd

3.3.2 5-methyl-uridine (5mUrd)

Figures 15 show ten representative trajectories which were run for up to 1000 fs. Overall, we observe a hop to the ground state in 40 of all 59 trajectories. Thereby, we document only one trajectory which hops to the ground state earlier than 200 fs (Figure 15a) where the decay is facilitated through N_1-C_2 bond breaking on a 100 fs time scale (conical intersection shown in Figure 26). In the majority of the remaining cases the decay decay to the ground state is facilitated through CH_3 out-of-plane puckering (conical intersections shown in Figure 26), whereas we observe a clear preference for down-puckering (31 trajectories, Figure 15,b-d) with respect to up-puckering (15 trajectories, Figure 15e-g). The hopping events are evenly distributed between 200 and 1000 fs with 12 hops between 200-400 fs, 11 hops between 400-600 fs, 7 hops between 600-800 fs and 10 hops between 800-1000 fs. In 19 trajectories no hop was encountered on the time scale of the simulation (Figure 15i,j). Thereof, in 7 the CI region is reached but no hop is observed (Figure 15i). In 3 of these cases the CI region is reached towards the end of the simulation (trajectories 49, 51 and 52). In the other 4 cases we observe a O_8-puckering causing the simulation to break due to a pronounced lone pair mixing in the active space (trajectories 53, 55, 57 and 58, Figure 15h). These 7 trajectories are regarded as decaying, thus resulting in a total a 47 hopping trajectories within the time scale of the simulation.

The CASPT2 dynamics with the full valence-π active space are overall stable, although with more pronounced deviations compared to Urd. As in the case of Urd, in the ground state the total energy increase is more pronounced due to the large gradient. Increasing the active space by two extra-valence orbitals reduces substantially the deviation of the total energy (Figure 16). Hence, the CAS(10,10) trajectories were utilized in the stochastic analysis and spectra simulations instead of their CAS(10,8) counterpart. Only trajectories which show total energy deviation above 10 kcal/mol on the excited state (16 in 5mUrd) were repeated with the larger active space. We note that the two sets of trajectories, SS-CASPT2/SA-2-CASSCF(10,8) (red) and SS-CASPT2/SA-2-CASSCF(10,10) (black) in Figure 16, behave qualitatively similarly on a short time scale (up to 200-300 fs). However, due to the significantly longer life time of 5mUrd the dynamics diverge beyond 400 fs. The CAS(10,10) dynamics show an accelerated decay, albeit still in the few-hundred femtosecond range.

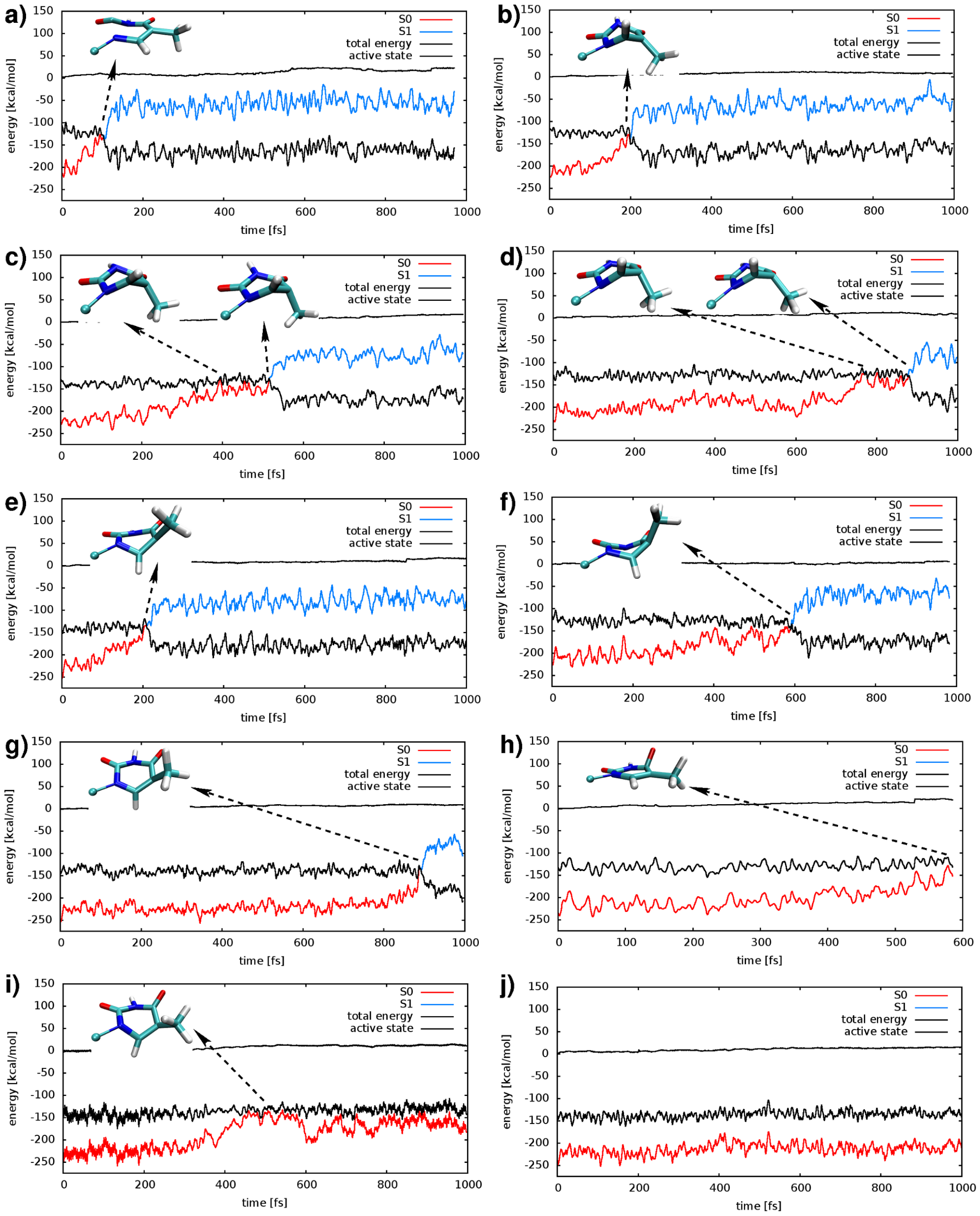

Supplementary Figure 15: a)-j): Ten representative trajectories for 5mUrd

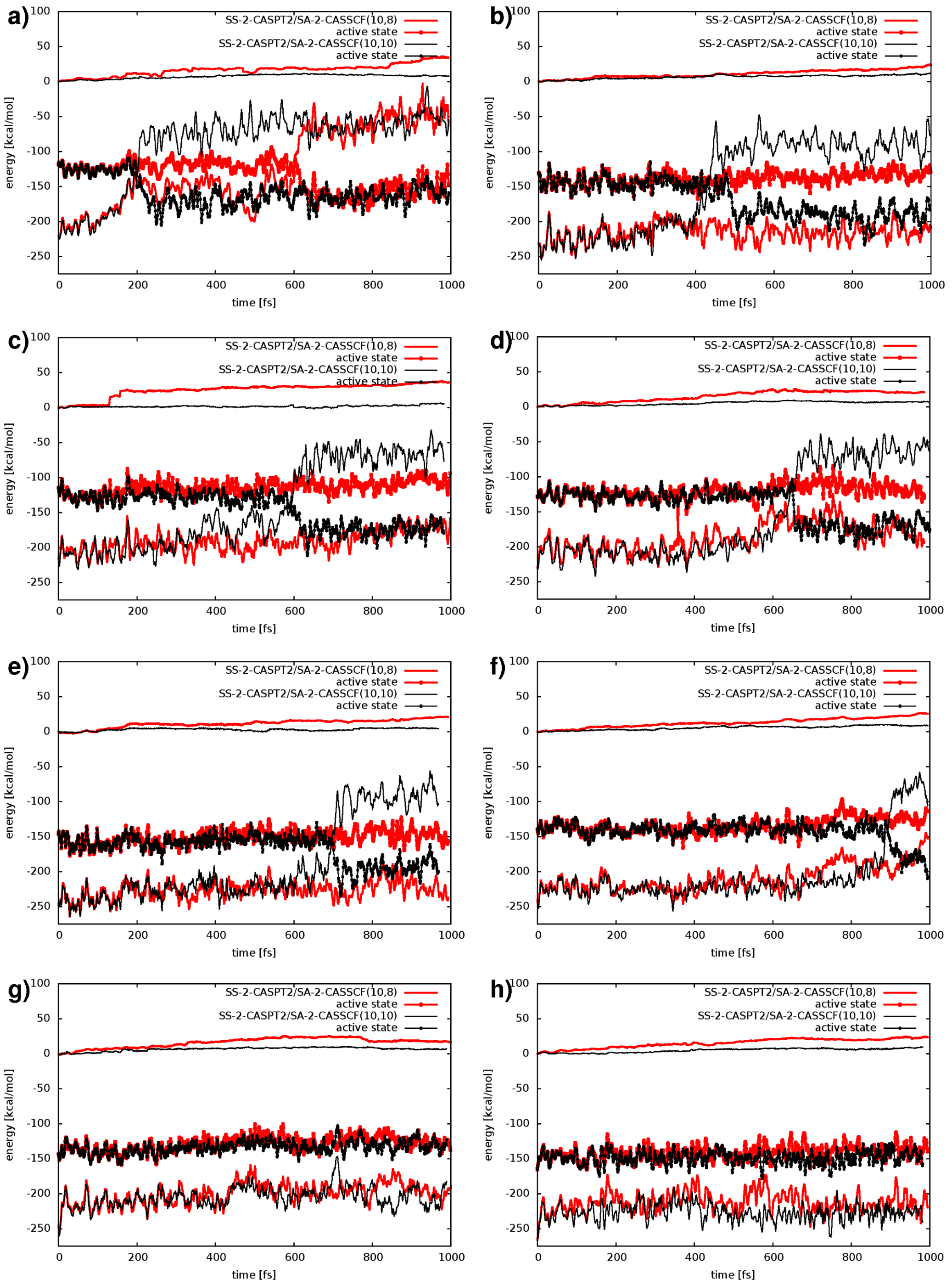

Supplementary Figure 16: a)-h): Comparison between eight representative trajectories computed at the SS-2-CASPT2/SA-2-CAS(10,8) (red) and at the SS-2-CASPT2/SA-2-CAS(10,10) (black) levels of theory in 5mUrd

3.4 Comparison of SS-, MS- and XMS-CASPT2 dynamics in Uridine

The following dynamics protocols were benchmarked on Uridine:

- the full-π SS-2-CASPT2/SA-2-CASSCF(10,8) level with a state averaging covering the ground state and the lowest $\pi\pi^*$ root on a set of 57 trajectories (the production run)
- the full-π MS-4-CASPT2/SA-4-CASSCF(10,8) level with a state averaging covering the ground state and the lowest three $\pi\pi^*$ roots on a subset of 50 trajectories from the production run
- full-π+2n(O) XMS-9-CASPT2/SA-9-CASSCF(14,10) level with a state averaging covering the lowest nine roots - the ground state, four $\pi\pi^*$ and four $n\pi^*$ states - on a subset of 25 trajectories from the production run.

In the following we compare 25 the subset of 25 trajectories common to all three levels, five representative cases are presented in Figures 17-21. Following observations can be made:

- the XMS-CASPT2 dynamics reproduce closely the SS-CASPT2 results (Figures 17-19): in 18 out of 25 trajectories the decay to the ground state happens at approximately the same time through a CI with a nearly identical structure; overall XMS-CASPT2 tends to be slightly faster compared to SS-CASPT2 (Figure 19);
- occasionally (in the case of XMS-CASPT2, Figure 20) and as a general rule (in the case of MS-CASPT2, Figures 18-20) the multi-state CASPT2 dynamics exhibit avoided crossings and no hopping to the ground state where SS-CASPT2 shows a real crossing accompanied by a hop; This makes MS-CASPT2 much slower than either SS- or XMS-CASPT2: only 4 trajectories show similar behavior to the other two methods (Figure 17). In all other cases the hopping event is either considerably delayed or does not occur within the simulated time; The reason for that is the strong sensitivity of the method to the active space size which is discussed in more detail below;
- in nearly all trajectories XMS-CASPT2 trajectories the nature of the active state remains 1$\pi\pi^*$, even when the dynamics exhibits excited state crossings (Figure 18) which is corroborated by the fast decay to the ground state instead of a trapping as would be expected for

dynamics in the $n\pi^*$ state. We identified merely two cases where the $1n\pi^*$ state gets involved which expectedly give rise to a longer living excited state (Figure 21).

The benchmarking demonstrates an outstanding agreement between SS- and XMS-CASPT2 flavors and supports the secondary role of the $1n\pi^*$ state in the deactivation process.

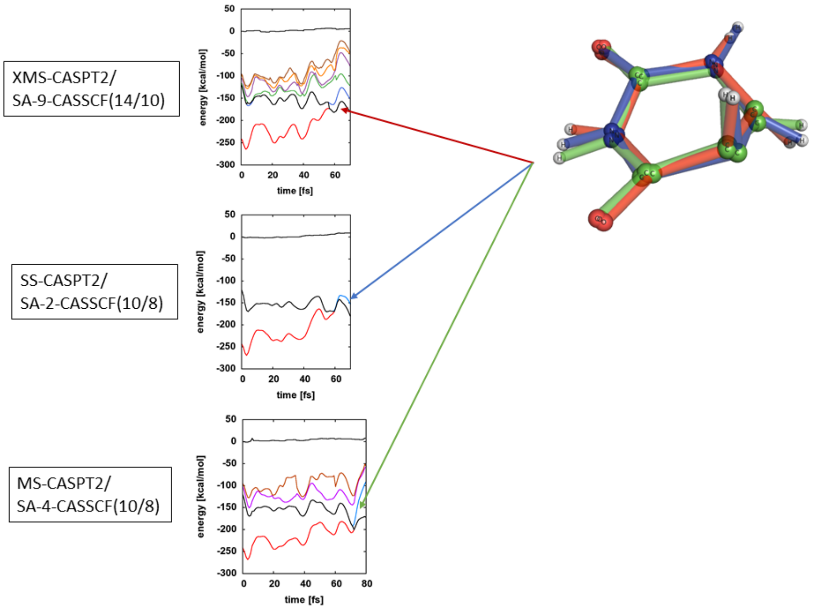

Supplementary Figure 17: Comparison between XMS- (top), SS- (middle) and MS- (bottom) CASPT2 levels of theory together with the structure of the hopping geometry for a representative Urd trajectory.

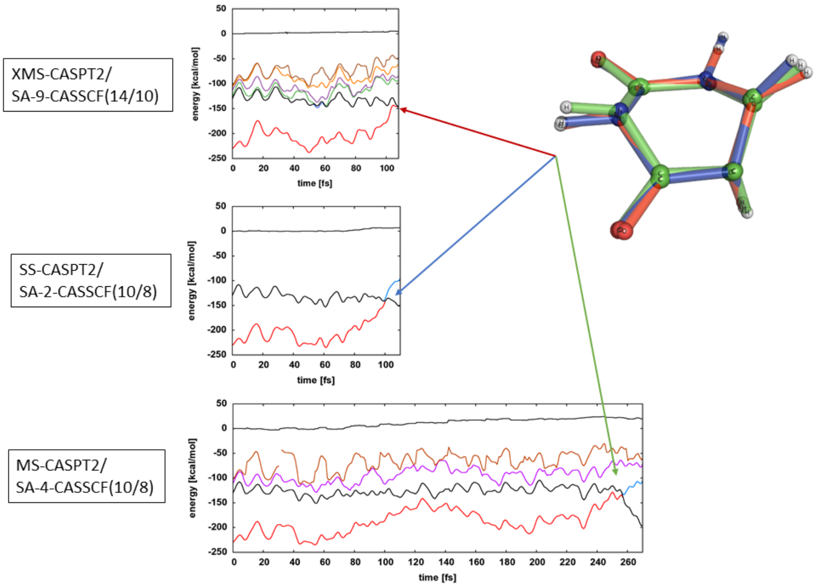

Supplementary Figure 18: Comparison between XMS- (top), SS- (middle) and MS- (bottom) CASPT2 levels of theory together with the structure of the hopping geometry for a representative Urd trajectory.

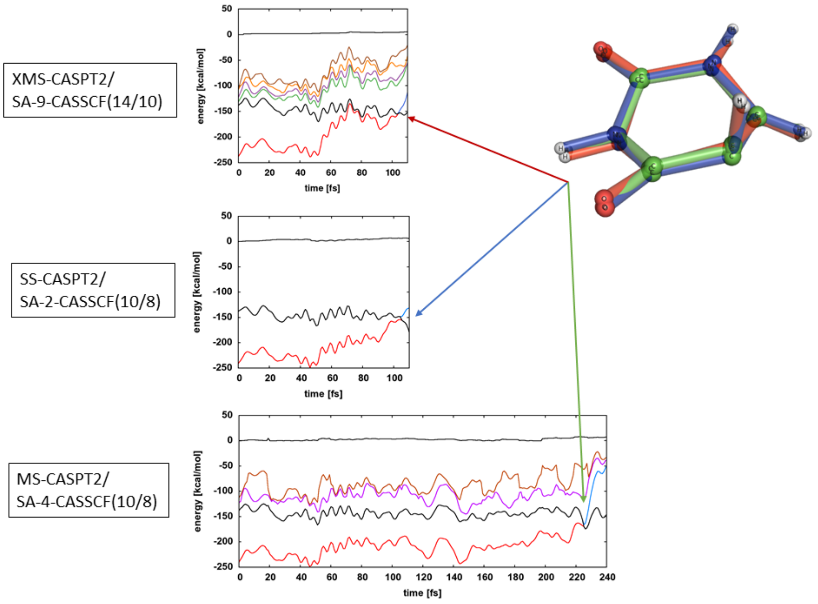

Supplementary Figure 19: Comparison between XMS- (top), SS- (middle) and MS- (bottom) CASPT2 levels of theory together with the structure of the hopping geometry for a representative Urd trajectory.

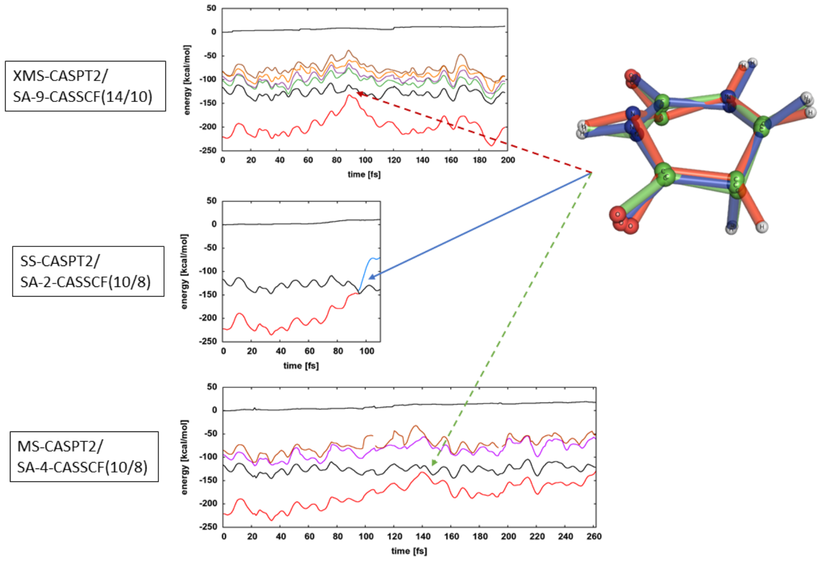

Supplementary Figure 20: Comparison between XMS- (top), SS- (middle) and MS- (bottom) CASPT2 levels of theory together with the structure of the hopping geometry for a representative Urd trajectory.

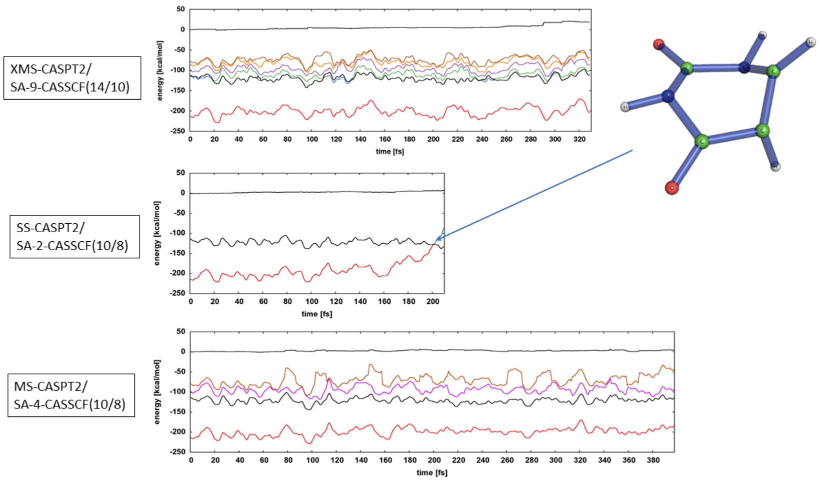

Supplementary Figure 21: Comparison between XMS- (top), SS- (middle) and MS- (bottom) CASPT2 levels of theory together with the structure of the hopping geometry for a representative Urd trajectory.

We now focus on the pronounced discrepancy of MS-CASPT2 dynamics. While the majority of SS- and XMS-CASPT2 trajectories have decayed to the ground state after 200 fs, the MS-CASPT2 dynamics are considerably slower with half of the trajectories not having decayed within the simulation period. While in a few cases single state and multi state simulations agree fairly well (e.g. Figure 17), in most cases we observe that the system roams in the CI region in the MS-CASPT2 simulations. This observation extends over the subset of 50 trajectories common to both levels of theory. The reason for this discrepancy is an artifact of the MS-CASPT2 approach with small active spaces that gives rise to relatively large off-diagonal S_0-S_1 elements in the MS-CASPT2 Hamiltonian (an order of magnitude larger than the value of 0.003 a.u. suggested [Ser05]). While not posing a problem in the FC region where the S_0-S_1 energy gap is large, these couplings become important in the cases when the two states come energetically close. In a direction in which the CASSCF wave functions mix, the MS-CASPT2 routine over-estimates the level of un-mixing thus pushing the two states apart (e.g. Figurs 18-20), introducing an artificial barrier in the excited state and preventing an efficient decay. To demonstrate the tendency of MS-CASPT2 to overestimate the splitting we document the distribution of energy gaps at the SS-CASPT2 and MS-CASPT2 level at the CIs encountered along the MD simulations in Figure 22. Thereby, we selected the geometry with the lowest S_1-S_0 energy gap along each trajectory (which in most cases coincides with the geometry at which the hopping occurs) and computed SS-2-CASPT2 and MS-2-CASPT2 energies with two active spaces, the full-π CAS(10,8) used in the simulations and a large RAS$(4,5|0,0|4,11)$ augmented with eight virtual orbitals. It can be seen that, while the energy gap at the SS-CASPT2 level does not change as a function of the active space (Figure 22,a), it is very much affected at the MS-CASPT2 level where the splitting is halved (Figure 22,b). The splitting is directly related to the magnitude of the MS-CASPT2 couplings at both levels (Figure 22,c). The only way for the excited state to decay to the ground state is in the form of a diabatic crossing where the two states do not mix at the CASSCF level but the system has to roam longer on the $\pi\pi^*$ potential energy surface to find such a crossing. Comparison with the experimental transient data clearly shows that the majority of the population has decayed to the ground state within 200 fs and that the dynamics is completed within 500 fs, in better agreement with the SS- and XMS-CASPT2 results.

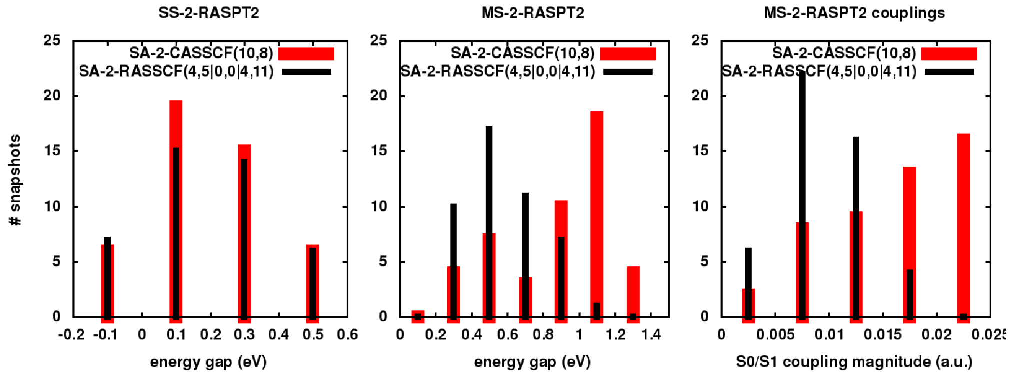

Supplementary Figure 22: Comparison between the distribution of energy gaps at the conical intersections encountered along the MD simulations: Left: SS-2-CASPT2/CAS(10,8) (red) and SS-2-RASPT2/RAS$(4,5|0,0|4,11)$ (black) levels; Middle: at MS-2-CASPT2/CAS(10,8 (red)) and MS-2-RASPT2/RAS$(4,5|0,0|4,11)$ (black) levels. Right: comparison at the MS-CASPT2 couplings between S_0 and S_1 at the MS-2-CASPT2/CAS(10,8) (red) and MS-2-RASPT2/RAS$(4,5|0,0|4,11)$ (black) levels.

3.5 MS-CASPT2/SA-6-CASSCF(14,10) plots along trajectories: adiabatic vs. diabatic picture

For reasons elucidated in Supplementary Note 2.5.2 the lone pairs of the oxygen atoms were not included in the active space used to perform the mixed quantum classical molecular dynamics simulations (thus denoted π-only active space). The involvement of higher lying excited states was estimated ad-hoc by computing the energetics along the trajectories at the MS-6-RASPT2/SA-6-RASSCF$(4, 5|0, 0|4, 7)$ level, thus considering four electronic states above the lowest $\pi\pi^*$ state and analyzing the probability for population transfer therein.

Figures 23a-f (Urd) and 24a-f (5mUrd) show a detailed analysis for several representative trajectories. The individual panels contain the following information (from top to bottom):

(A) shows the energetics of the ground (red) and S_1 (blue) states at the π-only SS-2-CASPT2/SA-2-CASSCF(10,8) level (solid lines), as well as the energetics of the diabatic states D_0, $D_1(\pi\pi^*)$ and D_2 resulting from the diabatization (sec. 2.6) of the lowest three states computed at the MS-6-RASPT2/SA-6-RASSCF$(4, 5|0, 0|4, 7)$ level (dotted lines) along the π-only trajectories.

(B) documents the evolution of the $D_1(\pi\pi^*)$-D_0 energy gap (diagonal elements of the diabatic Hamiltonian)

(C) documents the evolution of the D_2-$D_1(\pi\pi^*)$ energy gap (diagonal elements of the diabatic Hamiltonian)

(D) shows the evolution of the magnitude of the $D_0 \rightarrow D_1(\pi\pi^*)$ (black) and $D_0 \rightarrow D_2$ (green) transition dipole moments.

(E) documents the population dynamics in the diabatic states D_0, D_1 and D_2 (blue, black and red solid lines, respectively).

We remind that the nature of D_2 can change along the trajectories as the $n\pi^*$ and second $\pi\pi^*$ states often swap order due to their energetic proximity. This state swapping can be recognized by inspecting the magnitude of the $D_0 \rightarrow D_2$ transition dipole moment in panel (D). While the photoactive state $D_1(\pi\pi^*)$ shows a constant value around 2 Debye which decreases to zero towards the crossing region, the oscillator strength of D_2 often exhibits sudden jumps between zero and 2 Debye, an indicator of its variable nature. This does not affect the dynamics on the photoactive state when it happens outside of the energy window considered to give rise to a notable state mixing

(D_2-D_1 gap below 10 kcal/mol). Thus, panel (D) shows the effective probability for the population to depart from the photoactive $\pi\pi^*$ state to the energetically closest electronic state.

3.5.1 Uridine (Urd)

Analysis of wavefunction mixing in the FC region The wavefunction analysis in the Franck-Condon region shows that six out of 56 trajectories ($\sim 10\%$) exhibit pronounced $\pi\pi^* - n\pi^*$ mixing which makes it impossible to perform initial assignment of the adiabatic states. In 18 of the remaining 50 trajectories (1/3) we observe a weak mixing (with $n\pi^*$ CSF coefficients in the $\pi\pi^*$ wavefunction of up to 0.3). In the remaining 32 trajectories the $\pi\pi^*$ and $n\pi^*$ states could be clearly identified, and showed no significant mixing. In one case the $n\pi^*$ was found to be the lowest excited state in the Franck-Condon point. Based on this analysis, we can conclude that roughly 5% of population (half of the trajectories exhibiting strong wavefunction mixing) would immediately adopt $n\pi^*$ due to the wavefunction mixing in the Franck-Condon region.

Analysis of the $n\pi^*$ energetics along the π-only trajectories Including the oxygen lone pairs in the active space seemingly affects strongly the energy profiles of the electronic states (compare solid vs. dotted profiles in panels (A) of Figure 23a-f). The trajectories with and without lone pairs in the active space often exhibit considerably differing profiles (e.g. 23c). However, the $\pi\pi^*$/ground state energy gaps remain quasi-identical (see panel (B) in Figure 23a-f) except for the crossing region where the MS-CASPT2 calculations result in large state splitting as discussed earlier (sec. 3.4). This indicates that the energy profiles of S_0 and S_1 are modulated in the same manner by the presence of the oxygen lone pairs in the active space.

Regarding the relative positions of the states $D_1(\pi\pi^*)$ and D_2, it is evident that they are close in the FC region (often within 10 kcal/mol, see also Figure 12) but diverge towards the conical intersection. We observe oscillatory dynamics for the D_2-D_1 energy gap with a period of ca. 20 fs (see panel (C) in Figure 23a-f which correlates to the period of the oscillation of the double bonds, thereby often reducing the gap to a few kcal/mol. Nevertheless, an inversion of the energies of D_1 and D_2 is observed only on three occasions (e.g. Figure 23e,f). Furthermore, we generally observe a weak and temporally confined mixing of the diabatic states (indicated by the small off-diagonal elements in the diabatic Hamiltonian). Thus, overall, Urd rarely explores regions of state-mixing and when this is the case it spends therein only a few femtoseconds.

Non-adiabatic dynamics (including the $n\pi^*$ state) in the diabatic representation Finally, we used the diabatic profiles and propagated the amplitudes of the electronic wavefunction starting with amplitude 1.0 in the $D_1(\pi\pi^*)$ state. Panels (E) of Figure 23a-f show the evolution of the population along each trajectory[6]. Due to the weak wavefunction mixing, the population of D_1 rarely deviates from unity and, consequently, we find low probability for population transfer to another excited state. In ten repetitions of the dynamics of the entire set of trajectories we found that on average 15% of the trajectories depart from the $D_1(\pi\pi^*)$ state before a funnel to the ground state is reached.

Conclusion Thus, based on the above analysis we estimate that in Urd, pumped at 275 nm, an upper limit of 20% of the population could potentially evolve on a different electronic state (an $n\pi^*$ or a higher lying $\pi\pi^*$ state). The D_2-D_1 energy gap dynamics exhibit a high-frequency oscillatory behaviour which can be traced back to double bonds vibrations in the aromatic ring.

[6] In order to explore the hopping probability along the entire trajectory hopping was suspended.

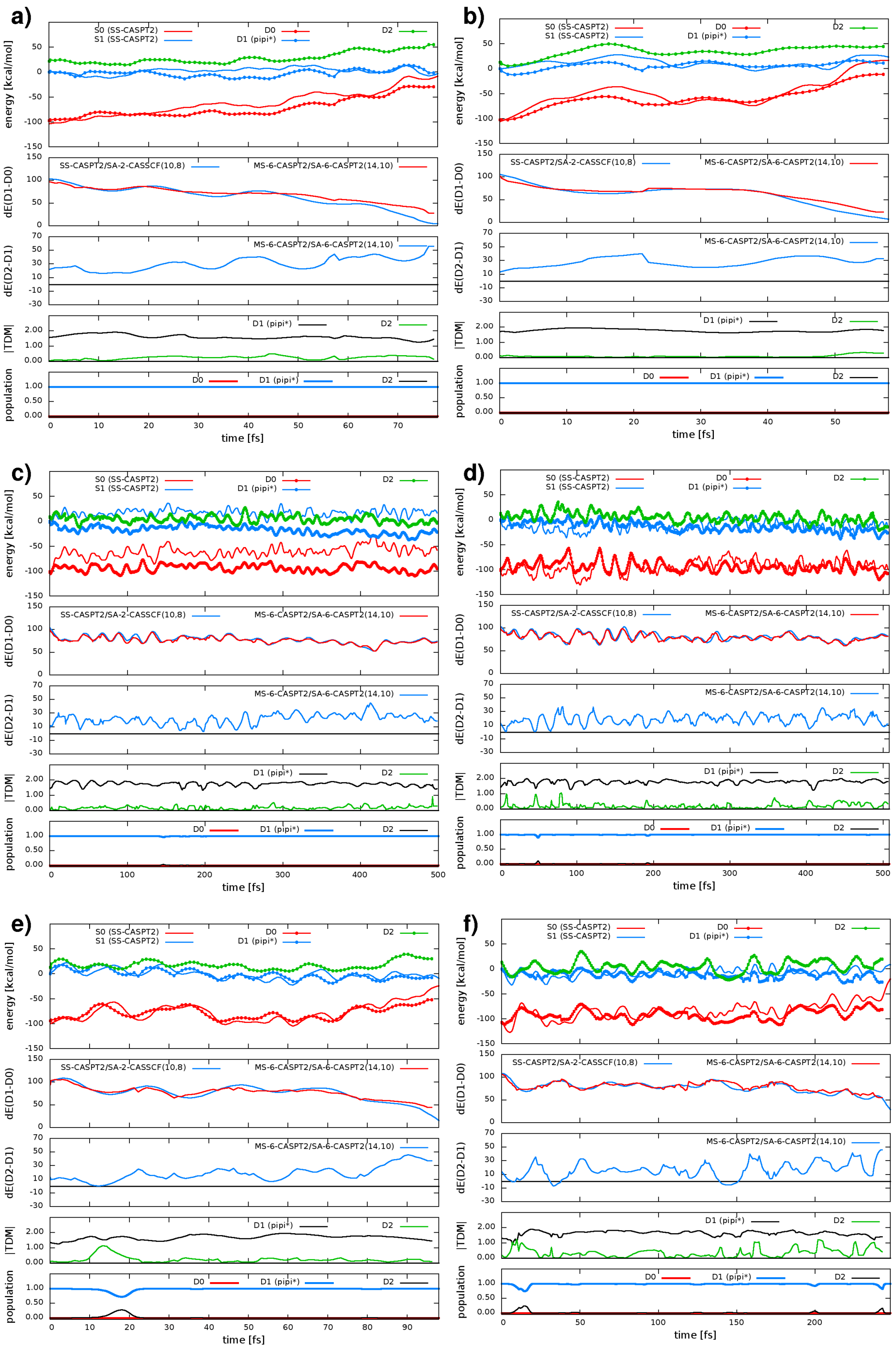

Supplementary Figure 23: a)-f): Analysis of the diabatic dynamics along six representative trajectories of Urd.

3.5.2 5-methyl-uridine (5mUrd)

Analysis of wavefunction mixing in the Franck-Condon region The wavefunction analysis in the Franck-Condon region shows that, in contrast to Urd, 5mUrd does not exhibit pronounced $\pi\pi^* - n\pi^*$ wavefunction mixing. In fact, in no case we find strong mixing, while we observe a weak mixing (with $n\pi^*$ CSF coefficients in the $\pi\pi^*$ wavefunction of up to 0.3) in seven out of the 54 analyzed trajectories ($\sim$ 13%). This trend is in line with the larger $\pi\pi^* - n\pi^*$ energy gap found for 5mUrd (0.50-0.55 eV) compared to Urd (0.35-0.40) as shown in Figure 12. Furthermore, we observe slightly more often (six cases) state swapping between the lowest $n\pi^*$ state and the second $\pi\pi^*$ state, which was encountered three times for Urd. This finding is in line with the predicted stronger involvement of the second $\pi\pi^*$ state in the photophysics of the 5mUrd in comparison to Urd [Pepino17]. However, we emphasize that we do not observe mixing of the lowest two $\pi\pi^*$ states. We expect this to be the case when pumping in the blue part (e.g. 260-240 nm) of the lowest absorption band in the LA spectrum (see Figure 11). Based on this analysis, we can conclude that immediate population of the $n\pi^*$ state is improbable in the Franck-Condon region.

Analysis of the $n\pi^*$ energetics along the π-only trajectories Most of the conclusions drawn for Urd apply also in the case of 5mUrd. The $\pi\pi^*$/ground state energy gaps remain quasi identical in all cases except for the crossing region where the $\pi\pi^*$/ground state degeneracy is often lifted and the splitting can reach up to 50 kcal/mol (panels (B) in Figure 24,a-f). Regarding the relative positions of the $D_1(\pi\pi^*)$ and D_2 we observe state mixing even more rarely compared to Urd. Inversion of the energies of D_1 and D_2 is again observed only at three occasions and is temporarily confined to a few femtoseconds. In contrast to Urd the magnitude of the D_0-D_2 transition dipole moment frequently exhibits sudden jumps between zero and ca. 2 Debye, an indicator of the constant swapping of $n\pi^*$ and the second $\pi\pi^*$ states (panels (D) in Figure 24,a-f).

Non-adiabatic dynamics (including the $n\pi^*$ state) in the diabatic representation Finally, we used the diabatic profiles and propagated the amplitudes of the electronic wavefunction starting with an amplitude 1.0 in the $D_1(\pi\pi^*)$ state. Panels (E) of Figure 24,a-f show the evolution of the population along each trajectory[7]. Due to the larger (in comparison to Urd)

[7] In order to explore the hopping probability along the entire trajectory hopping was suspended.

energy gap to the higher lying state leading to a weak wavefunction mixing, the population of D_1 rarely deviates from unity and, consequently, we find lower probability for population transfer to other excited states compared to Urd. In ten repetitions of the dynamics of the entire set of trajectories we found that on average merely 1% of the trajectories depart from the $\pi\pi^*$ state before the funnel to the ground state is reached.

Non-adiabatic dynamics (including the $n\pi^*$ state) in the adiabatic representation Considering the fact that the character of the S_1 state is dominated by the $\pi\pi^*$ configuration throughout the entire dynamics[8], we performed also ad-hoc non-adiabatic fewest switches surface hopping dynamics in the *adiabatic* representation in order to remove the bias of the diabatization procedure. To that aim we run the trajectories, re-using at each time step the velocities for the nuclei computed at the SS-2-CASPT2/SA-2-CASSCF(10,8) level[9] and propagated the amplitudes of the electronic wavefunction starting with an amplitude 1.0 in the S_1 state. At each step time-derivative couplings σ_{ij} were computed based on wave function overlaps (sec. 2.5) and hopping was realized through the usual stochastic procedure. The simulations confirm the findings of the dynamics performed in the diabatic representation, namely, that the S_2 state is not involved in the non-adiabatic dynamics. In ten repetitions of the dynamics of the entire set of trajectories we found that the population of S_1 rarely deviates from unity and on average merely 1% of the trajectories depart from the S_1 state before the funnel to the ground state is reached.

Conclusion Thus, based on the above analysis we estimate that in 5mUrd, pumped at 275 nm, the involvement of further excited state ($n\pi^*$ or higher lying $\pi\pi^*$) is marginal (around $\sim 1\%$).

[8] S_1 mixes weakly with higher lying electronic states along the dynamics and inversion of the $D_1(\pi\pi^*)/D_2$ energies is virtually not observed.

[9] Essentially, we assume that the S_1 does not mix with higher lying electronic states and their energetic vicinity does not affect the potential energy surface topology of S_1.

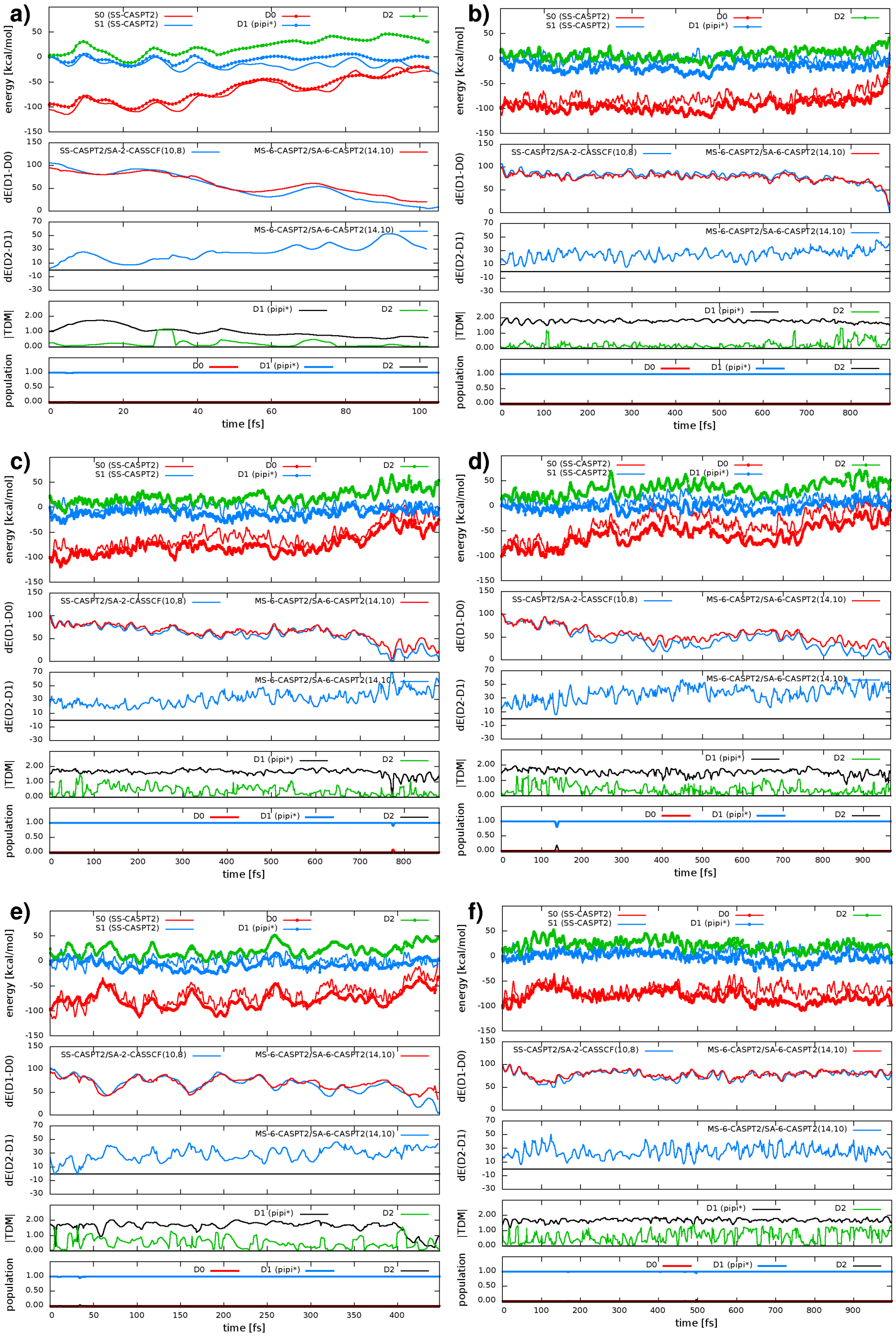

Supplementary Figure 24: a)-f): Analysis of the diabatic dynamics along six representative trajectories of 5mUrd.

3.6 Conical intersections

3.6.1 Uridine (Urd)

Figure 25 shows superimposed CIs encountered along the 59 trajectories of Urd. The decay to the ground state occurs in all cases through H_9 out-of-plane puckering, whereas we observe a clear preference for down-puckering with respect to up-puckering.

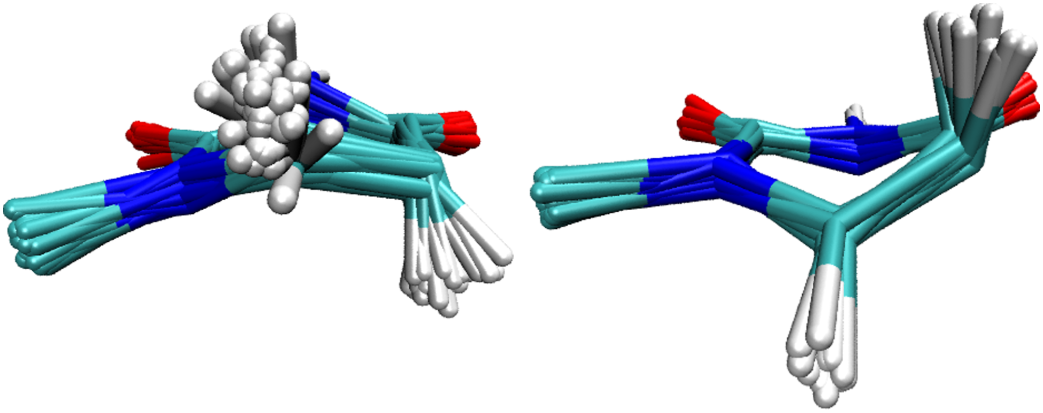

Supplementary Figure 25: Conical intersections in Urd: Left: hydrogen out-of-plane down-puckering (46); Right: hydrogen out-of-plane up-puckering (13). Points of degeneracy along the same trajectory separated by more than 20 fs were considered as different conical intersection geometries.

3.6.2 5-methyl-uridine (5mUrd)

Figure 26 shows superimposed CIs encountered along the 59 trajectories of Urd. The decay to the ground state occurs through three different mechanisms, the dominant one being the CH_3 out-of-plane puckering, whereas we observe a clear preference for down-puckering with respect to up-puckering. O_8 out-of-plane puckering and N_1-C_2 bond breaking are secondary channels.

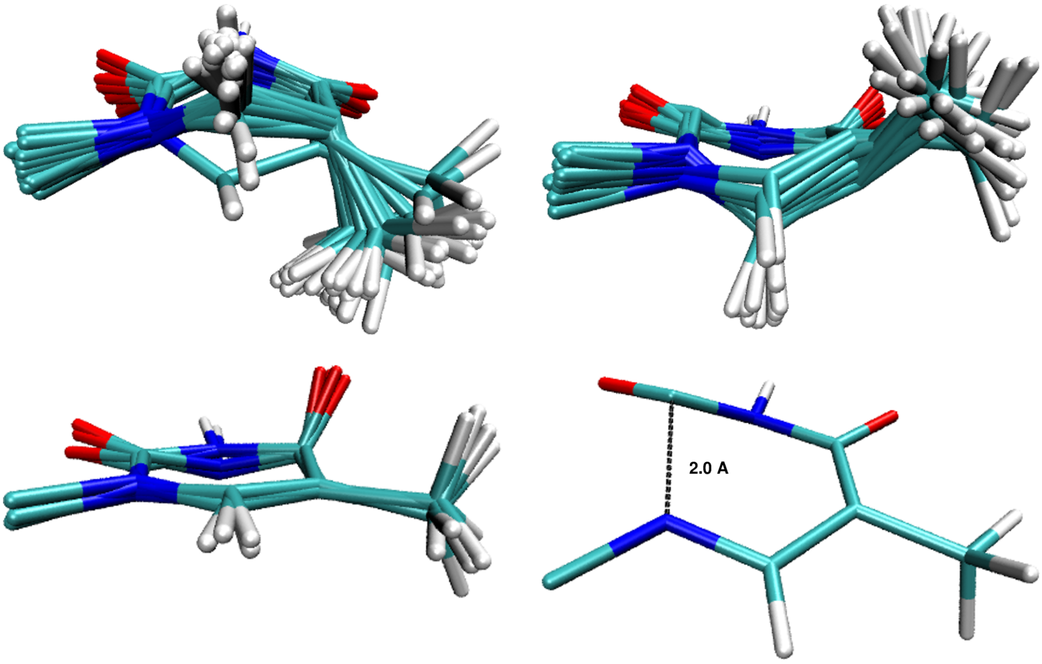

Supplementary Figure 26: Conical intersections in Urd: Top Left: methyl out-of-plane down-puckering (31); Top Right: methyl out-of-plane up-puckering (15); Bottom Left: O_4 out-of-plane puckering (4); Bottom Right: N_1-C_2 bond breaking (1). Points of degeneracy along the same trajectory separated by more than 20 fs were considered as different conical intersection geometries.

3.7 Analysis of vibrational coherences

To obtain a better idea of the ES vibrational dynamics of Urd and 5mUrd we performed a normal mode analysis. To this aim, we optimized the nuclear coordinates in the S_1 ($\pi\pi^*$) state utilizing the same QM/MM set up outlined in Supplementary Note 2.2 and using the SS-2-CASPT2/SA-2-CASSCF(10,8)/ANO-L[321,21] level of theory of the High Layer. The optimized S_1 geometries superimposed over the ground state equilibrium geometries are shown in Figure 27. We note a small distortion of the planarity of the aromatic ring, more pronounced in the 5mUrd. Subsequently, a frequency calculation was performed (numerically) at the same level of theory. All frequencies resulted positive, thus confirming the stationary nature of the obtained geometries.

Starting from the definition of the normal mode matrix

$$\mathbb{P}^{\dagger} = \mathbb{M}^{\frac{1}{2}} \nabla_{\tilde{\boldsymbol{q}}} \boldsymbol{Q} \tag{20}$$

where $\mathbb{P}$ is a matrix whose columns are the normal modes of the system $\tilde{\boldsymbol{q}}_k$ expressed in normalized mass-weighted Cartesian coordinates $\boldsymbol{Q}$ and $\mathbb{M}$ is a diagonal matrix with the nuclear masses, one can reformulate it in terms of finite differences

$$\mathbb{P}^{\dagger} \Delta \tilde{\boldsymbol{q}} = \mathbb{M}^{\frac{1}{2}} \Delta \boldsymbol{Q} \tag{21}$$

Thus, $\Delta \tilde{\boldsymbol{q}}$ represents the array of displacements $\tilde{\boldsymbol{d}}_{\boldsymbol{i}}$ along the normal modes of the system when the difference is taken with respect to the reference point on the $i-$the electronic state. Rearranging gives the final working equation

$$\tilde{\boldsymbol{d}}_i = \mathbb{P}^{-1} \mathbb{M}^{\frac{1}{2}} \Delta \boldsymbol{Q} \tag{22}$$

which allows to estimate how much every normal mode $\tilde{\boldsymbol{q}}_k$ has to be displaced to connect two points in Cartesian coordinate space of the potential energy surface of the $i-$th electronic state. $\Delta \boldsymbol{Q}$ is the difference in Cartesian coordinates between two geometries. The displacements $\tilde{\boldsymbol{d}}_i$ can be related to spectroscopic parameters like the Huang-Rhys factors S_{ik} or the reorganization energy λ_{ik} of the system:

$$S_{ik} = \frac{\tilde{d}_{ik}^2 \omega_k}{2\hbar} \qquad \text{and} \qquad \lambda_{ik} = \frac{\tilde{d}_{ik}^2 \omega_k^2}{2\hbar}. \tag{23}$$

where ω_k is the frequency of the $k-$th normal mode. With the normal mode analysis attention should be paid to global translational and rotational degrees of freedom which have to be removed prior to computing the difference in Cartesian coordinates. To this end we followed an iterative procedure

Table 3: Reorganization energy in selected normal modes of Urd

No.	ω (cm^{-1})	λ (cm^{-1})
1	468.8	169.9
2	627.1	154.8
3	713.4	382.7
4	772.1	847.2
5	882.2	107.1
6	970.3	115.1
7	1043.7	569.4
8	1449.3	110.3
9	1688.7	373.5
10	1852.2	1367.0

relying on the vectors of inertia in order to minimize the distance in space between two geometries as outlined in ref. [Kur01b].

In Tables 3 and 4 we list the modes accumulating the majority of the reorganization energy (> 90 cm^{-1}). Figure 28 shows the three modes accumulating the most reorganization energy.

The NMA shows that the 775 cm^{-1} breathing mode characterized by a large amplitude of the $N_1C_6C_5$ angle (numeration according to Figs. 4 and 7) is the dominant mode in the mid-frequency range of both systems (< 1000 cm^{-1}). This observation is in agreement with the Fourier transform of the residuals of the transient absorption spectra (Figure 3 of the main text). The ring breathing mode, together with the C_5C_6 (1044 cm^{-1}) and C_5C_6 (1852 cm^{-1}) stretch modes dominates the excited state vibrational dynamics of Urd. We note the significant red-shift of the frequency of the C_5C_6 stretch mode with respect to its ground state value of ca. 1700 cm^{-1}. Notably, in 5mUrd the C_5C_6 stretching is not among the active modes in the excited state. Instead, a notable amount is inserted in the symmetric CO stretching (1672 cm^{-1}), not directly involved in the hydrogen out-of-plane bending deformation facilitating the internal conversion to the ground state.

To correlate the findings of the NMA with the results of the Fourier transform analysis of the residuals of the transient absorption spectra (Figure 3 in the main text) we traced the evolution of the main geometrical deformation associated with the 770 cm^{-1} breathing mode, namely the $N_1C_6C_5$ angle, along the dynamics. Figure 29 shows heat maps of the fluctuation of the $N_1C_6C_5$ angle for Urd (top) and 5mUrd (bottom) as a function of time.

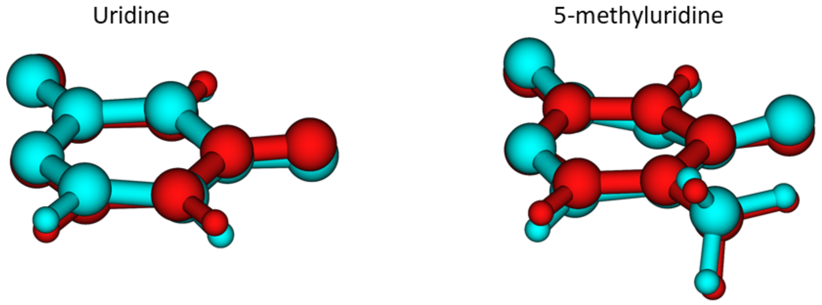

Supplementary Figure 27: Overlaid structures of the ground state and $\pi\pi^*$ excited state equilibrium geometries in Urd (left) and 5mUrd (right).

Table 4: Reorganization energy in selected normal modes of 5mUrd

No.	ω (cm^{-1})	λ (cm^{-1})
1	128.5	400.1
2	189.9	253.5
3	303.8	193.0
4	418.3	167.7
5	460.9	157.5
6	667.0	214.3
7	775.6	860.6
8	799.0	104.6
9	994.0	91.7
10	1299.9	254.1
11	1544.7	138.6
12	1672.4	1038.8
13	1731.6	978.6

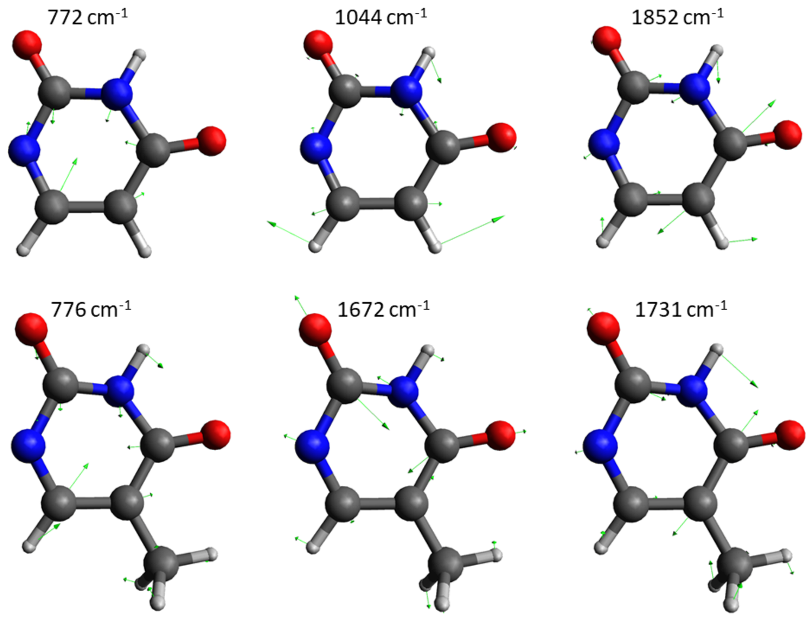

Supplementary Figure 28: Modes accumulating the most reorganization energy in Urd (top) and 5mUrd (bottom)

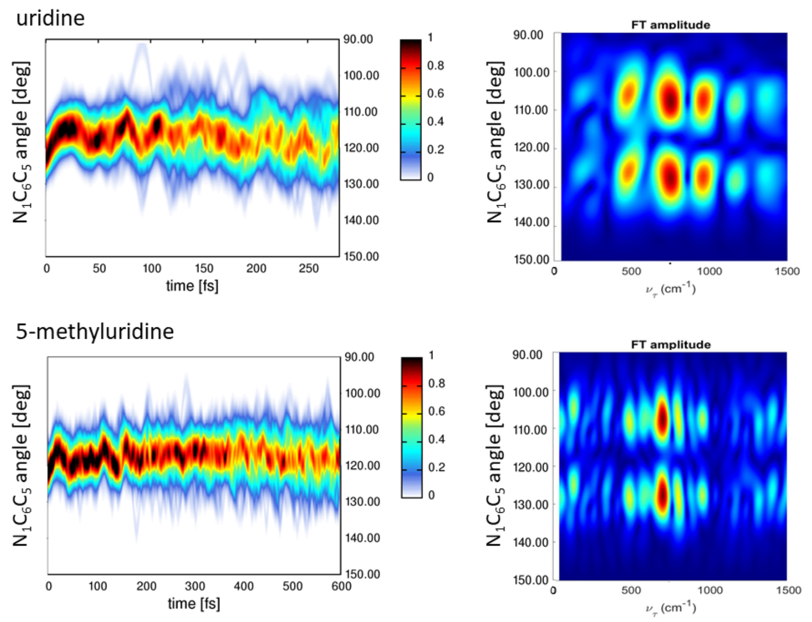

Supplementary Figure 29: Heat maps of the fluctuation of the $N_1C_6C_5$ angle (left) and their Fourier transform for Urd (top) and 5mUrd (bottom).

Fourier transform of the dynamics (until 250 fs for Urd and until 600 fs for 5mUrd) confirms that that $N_1C_6C_5$ angle exhibits a coherent oscillation with ca. 750 cm^{-1} frequency, as predicted by the NMA.

Similarly, we also traced the dynamics of the C_5C_6 bond and the $H_9/CH_3C_5C_6H_{10}$ hydrogen/methyl out-of-plane bending involved in the internal conversion to the ground state. Figure 30 and 31 show the evolution of the C_5C_6 bond length and $H_9/CH_3C_5C_6H_{10}$ dihedral angle during the MD in Urd (top) and 5mUrd (bottom). Regarding the C_5C_6 bond length we observe immediate elongation to 1.65 Å after populating the $\pi\pi^*$ state, followed by coherent oscillatory dynamics in the first few hundred femtoseconds. As Urd rapidly decays to the ground state the C_5C_6 bond length is restored to its ground state equilibrium value of 1.38 Å within 0.5 ps. As 5mUrd remains in the excited state on a picosecond time scale we observe oscillations around 1.45 Å in the first 0.4 ps, i.e. the value of the C_5C_6 bond length in the $\pi\pi^*$ equilibrium. After 0.4 ps a shift of the average C_5C_6 length to smaller values is visible. Fourier transform of the dynamics (until 250 fs for Urd and until 600 fs for 5mUrd) shows that in Urd the stretching is governed in the excited state by a single mode around 1000 cm^{-1}. This matches perfectly with the 1040 cm^{-1} active mode revealed by the NMA (Figure 28, top). In 5mUrd the stretching contributes to three modes, ca. 750 cm^{-1}, 1000 cm^{-1} and 1250 cm^{-1} which correspond to modes 7,9 and 10 in Table 4 whose Cartesian deformations show contribution by the C_5C_6 stretching.

The plot of the $H_9C_5C_6C_{10}$ dihedral angle evolution in Urd (Figure 31, top left) shows that it takes about 50-75 fs to distribute energy in the hydrogen out-of-plane bending mode. Between 50-250 fs we observe a scattered plot due to the decay to the ground state through the puckered conical intersection with values around 90° and -90°. 5mUrd takes considerably longer to distribute energy in the methyl out-of-plane bending mode (Figure 31, bottom left), i.e. around 250-300 fs. Puckering events are scattered in the plot.

We recognize that the aromatic ring exhibits a symmetry plane with respect to the hydrogen/methyl out-of-plane bending which is resembled in the two sets of conical intersections with the ground state which can be reached. Thus, in Figure 31, right, we plot the evolution of the absolute value of the dihedral $H_9/CH_3C_5C_6H_{10}$. This helps to recognize signs of coherent ultrafast deformation of the planarity of the aromatic ring in Urd where the hydrogen out-of-plane bending reaches values of up to 50-60°already after 25 fs before

[9] We note that the symmetry is broken in the presence of the surrounding (sugar and solvent) which might explain the preferred population of one of the two conical intersections in each nucleoside)

going back to 0°at 50 fs. The period associated with this ring deformation matches well the period (55 fs) associated with the 600 cm^{-1} peak appearing in the Fourier transform maps of Urd shown in Figure 3 of the main draft suggesting that this may be a spectral signature of the coherent ring deformation observed at early times in simulations. Thereby, a candidate for a driving mode is a 600 cm^{-1} hydrogen out-of-plane bending, immediately activated in the FC region. After the first period (ca. 50 fs) the coherence is partially destroyed and the individual trajectories reach the conical intersection region within a time window of three periods of the mode (most of the trajectories decay between 75 fs and 225 fs). Correspondingly Fourier transform of the heat maps (not shown) does not reveal clear vibrational signatures.

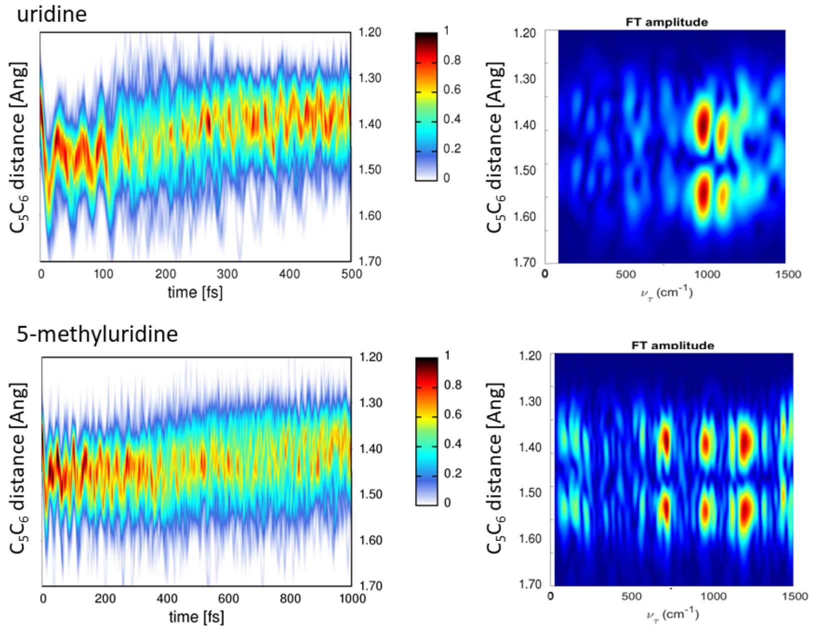

Supplementary Figure 30: Heat maps of the fluctuation of the C_5C_6 bond length (left) and their Fourier transform for Urd (top) and 5mUrd (bottom).

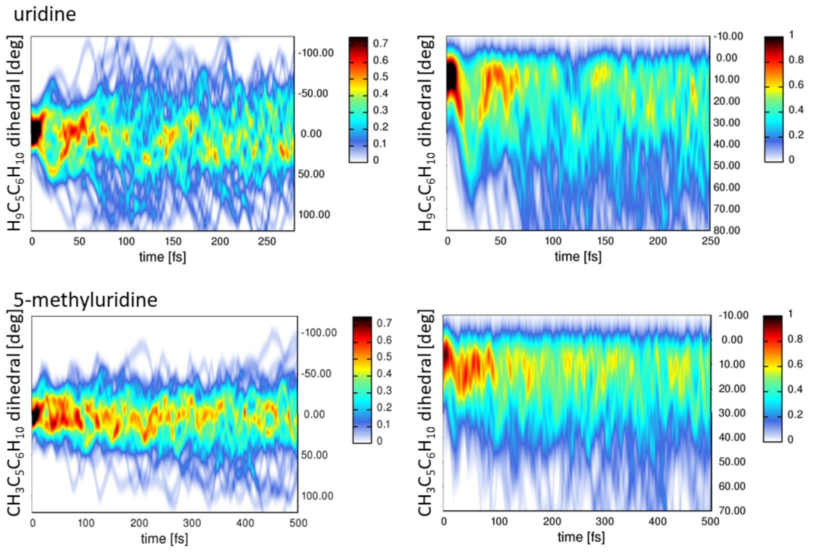

Supplementary Figure 31: Heat maps of the fluctuation of the $H_9/CH_3C_5C_6H_{10}$ dihedral (left: using real values; right: using absolute values) for Urd (top) and 5mUrd (bottom).

Supplementary Note 4:
Materials and Steady-State Spectroscopy

5mUrd (97% purity) and Urd (99% purity) were purchased from Sigma-Aldrich and used as received. A phosphate-buffered saline (PBS) solution was prepared by dissolving 3.6 g of sodium dihydrogen phosphate and 4.26 g of sodium hydrogen phosphate in ultrapure water to obtain a pH 7.4 and a concentration of 15 mM. The 5mUrd and Urd in PBS solutions were prepared to obtain a concentration of 24.2 and 27.6 mM. Different concentrations (from 0.4 mM to 30 mM) were tested without finding significant differences in their absorption spectra.

The steady-state absorption spectra were measured at room temperature using a V-570 Jasco spectrophotometer. They were background corrected by subtracting the solvent spectrum measured at the same experimental conditions. The absorption spectra of the samples used for the experiments reported in the paper are shown in Figure 32.

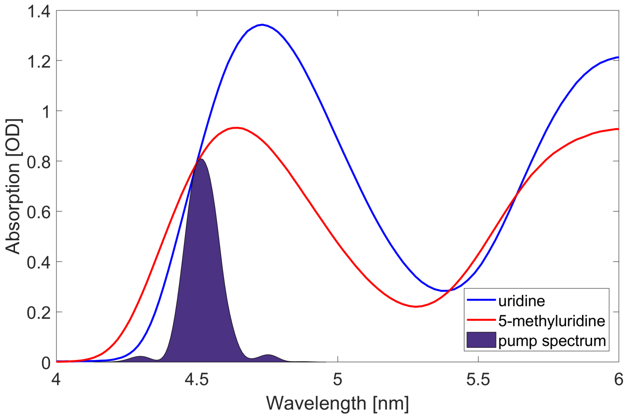

Supplementary Figure 32: Linear absorption spectra for Urd (blue) and 5mUrd (red) together with the pump pulse spectrum (purple).

Supplementary Note 5:
Transient absorption spectroscopy

5.1 Transient absorption spectra

Figure 33 shows the transient absorption spectra at different time delays between pump and probe (parallel polarizations) for 5mUrd (left) and Urd (right).

In 5mUrd the SE signal progressively decays within 1 ps presenting a red-shift. This decay is concomitant with that of the photo-induced absorption band peaked at 2 eV (PA1). A third spectral feature around 4.2 eV (PA2) is formed within 100 fs and decays blue-shifting on the picosecond time scale.

In Urd the SE band completely vanishes within 200 fs, followed by the formation of PA band (PA2) which reaches its maximum in about 300 fs. A photo-induced absorption signal in the visible (PA1) progressively decays in 500 fs.

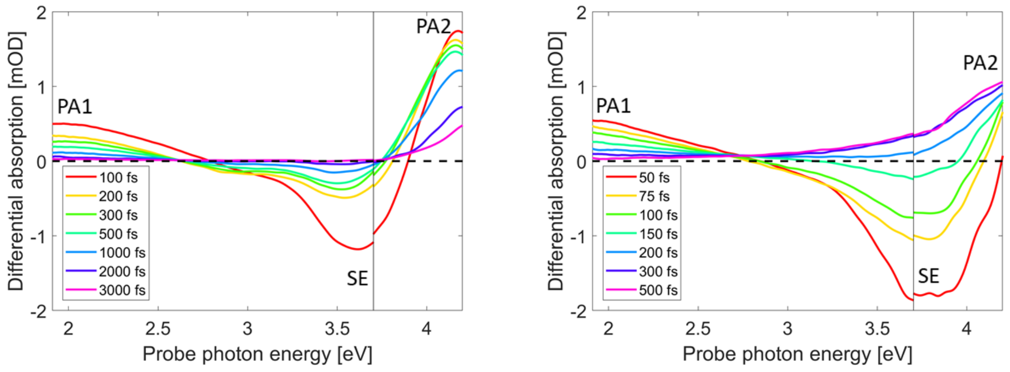

Supplementary Figure 33: Transient absorption spectra at selected time delays for 5mUrd (left) and Urd (rigth). The vertical line at 3.65 eV stands for the two probe spectral ranges used in the experiment.

5.2 Measurements on the pure solvent

Figure 34 shows the dynamics at selected energies of the phosphate buffer solution. Transient absorption spectra at different time delays between pump and probe (parallel polarizations) are reported in figure 35. No signal from solvated electrons was noticed under our experimental conditions.

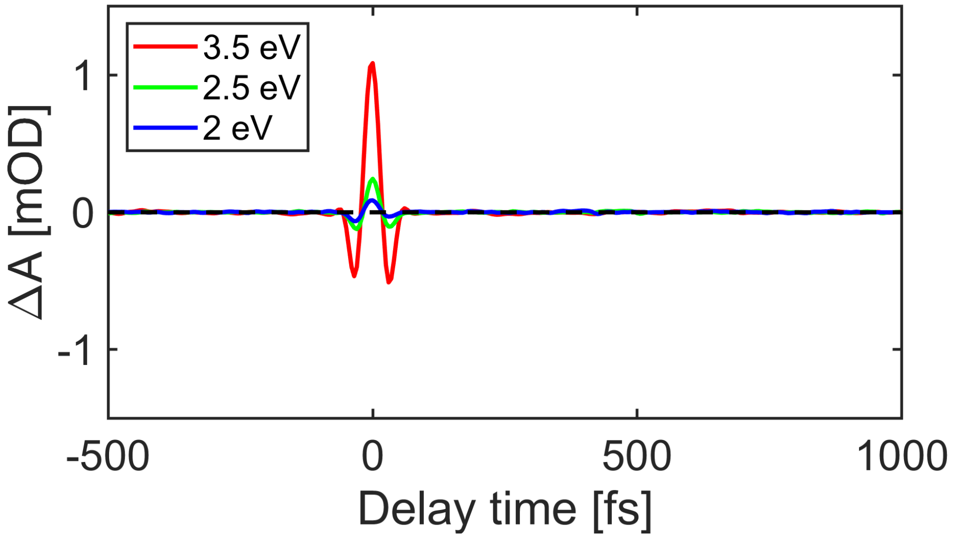

Supplementary Figure 34: Dynamics of the PBS solvent at selected energies.

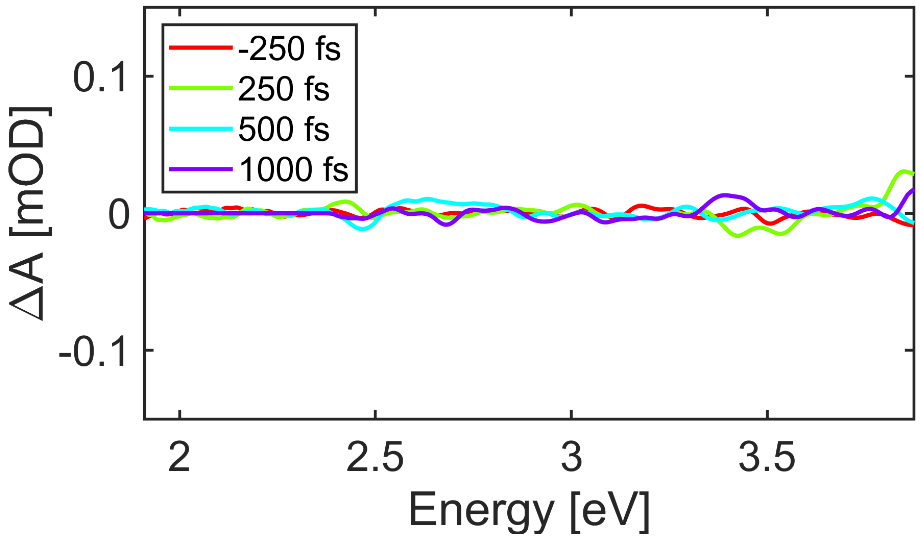

Supplementary Figure 35: Transient absorption spectra at selected time delays for the PBS solvent.

5.3 Measurements at magic angle and orthogonal polarizations

Figures 36 and 37 present the experimental and theoretical transient absorption maps for Urd at magic angle and crossed polarizations, respectively. In addition to the signals identified in the main text, a photo-induced absorption band (PA3) becomes visible when using orthogonal polarizations, due to the partial suppression of the SE signal.

Figures 38 and 39 present the experimental and theoretical transient absorption maps for 5mUrd at magic angle and crossed polarizations, respectively. In addition to the signals identified in the main text, a photo-induced absorption band (PA3), which follows the same trend as PA1, becomes visible for perpendicular polarizations.

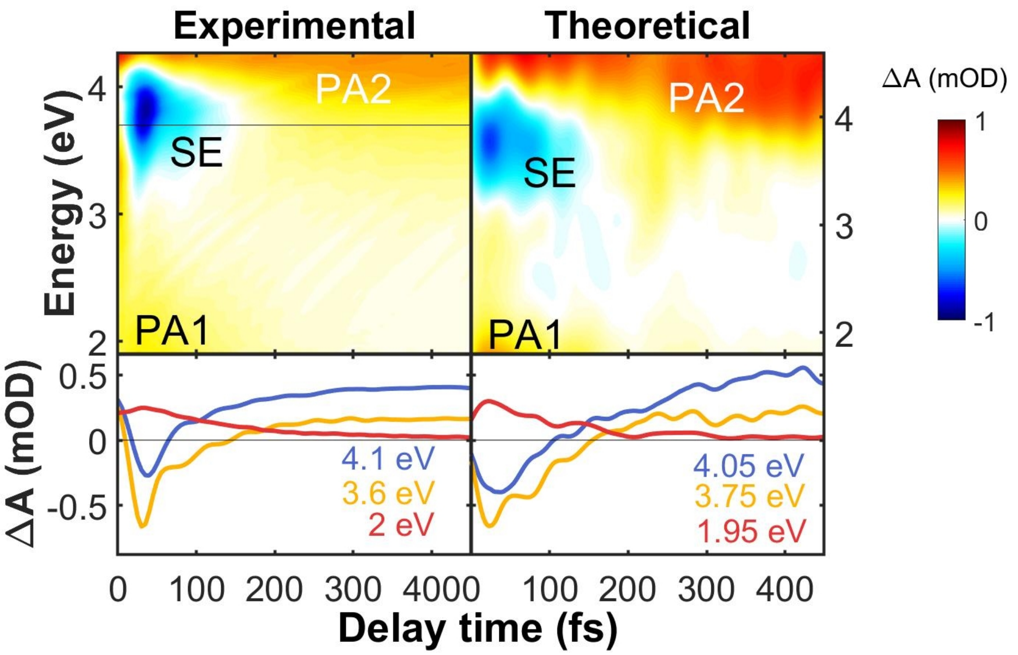

Supplementary Figure 36: Experimental (left) and simulated (right) transient absorption spectra maps (top) and selected dynamics (bottom) of Urd with the polarization of pump and probe at magic angle.

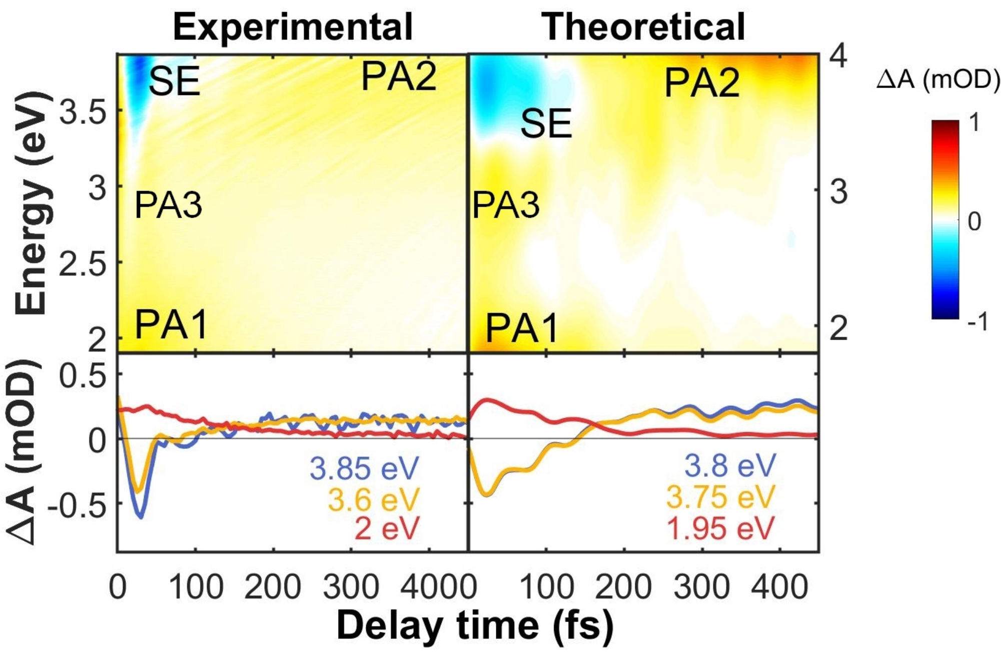

Supplementary Figure 37: Experimental (left) and simulated (right) transient absorption spectra maps (top) and selected dynamics (bottom) of Urd with orthogonal polarizations of pump and probe.

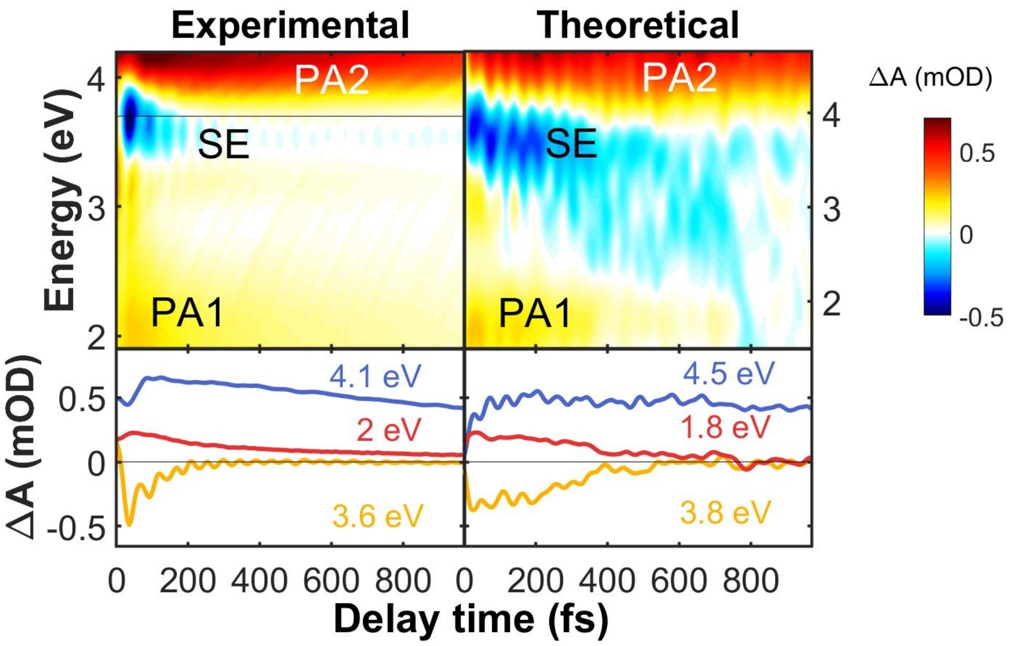

Supplementary Figure 38: Experimental (left) and simulated (right) transient absorption spectra maps (top) and selected dynamics (bottom) of 5mUrd with the polarization of pump and probe at magic angle.

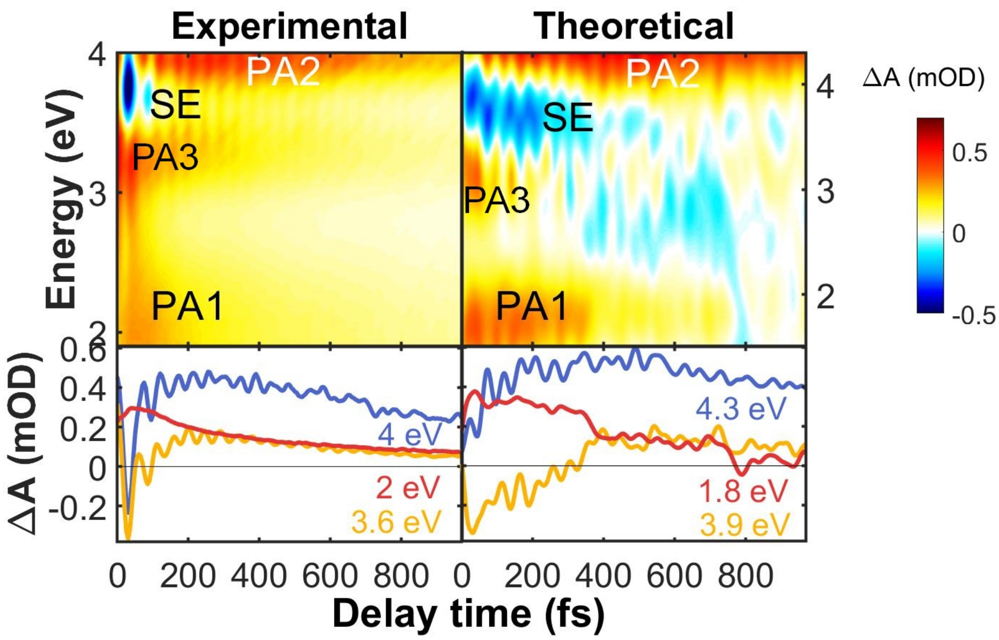

Supplementary Figure 39: Experimental (left) and simulated (right) transient absorption spectra maps (top) and selected dynamics (bottom) of 5mUrd with orthogonal polarizations of pump and probe.

5.4 Components of the simulated transient spectra

Figures 40 and 41 present individual components in the simulated transient absorption maps of Urd and 5mUrd at magic angle. Specifically, signals involving the ground state (SE and PA2, labels according to Figs. 1 and 2 of the main text) are shown separately from the remaining PA signals. We have deliberately used the same scale as in the total maps (Figures 36 and 38). This allows to appreciate the destructive overlap between signals of opposite sign, as well as the presence of characteristic S_1 fingerprint PA which remain covered in the total spectra. Note that the PA2 signal in the spectra of 5mUrd in Figure 41 is dominated by excited state PA at early times and by ground state PA at later times.

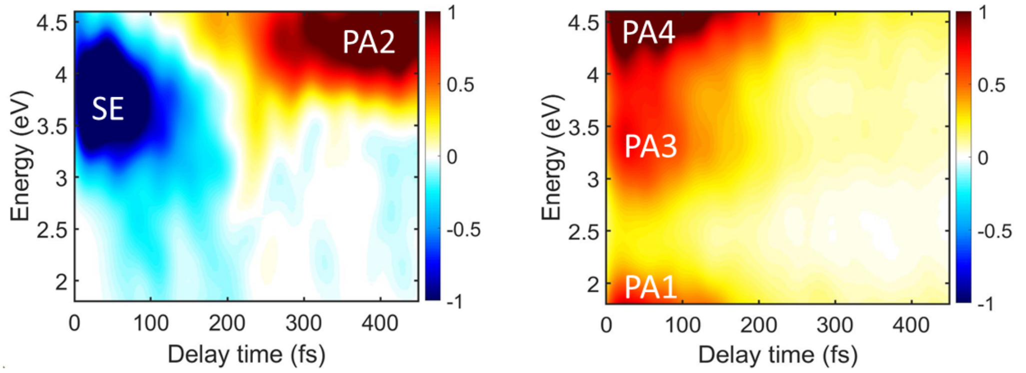

Supplementary Figure 40: Components of the simulated transient absorption spectra (with the polarization of pump and probe at magic angle) of Urd; left: Stimulated emission (SE) and photoinduced absorption of the ground state (PA2); right: excited state (S_1) photoinduced absorption signals PA1, PA3 and PA4. Due to destructive overlap PA3 and PA4 are not visible in Figure 1 of the main text. Same scale used as in the total map (Figure 36).

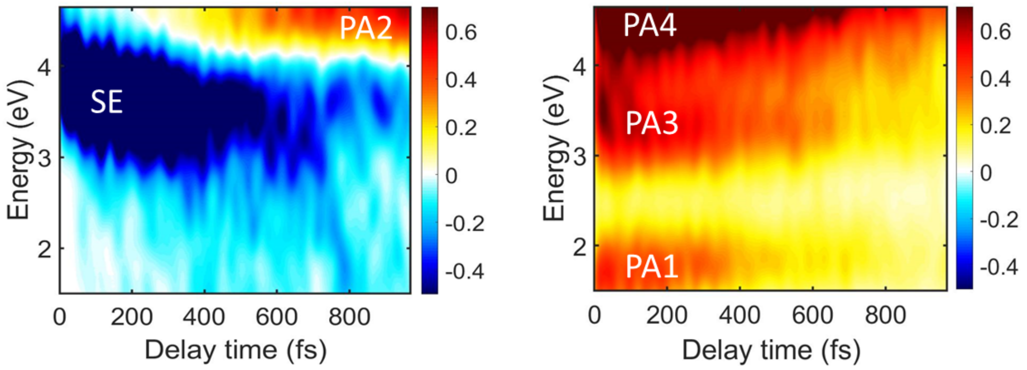

Supplementary Figure 41: Components of the simulated transient absorption spectra (with the polarization of pump and probe at magic angle) of 5mUrd; left: Stimulated emission (SE) and photoinduced absorption of the ground state (PA2); right: excited state (S_1) photoinduced absorption signals PA1, PA2 and PA3. Note that signal PA2 is dominated by excited state PA at early times and by ground state PA at later times. Due to destructive overlap PA3 is not visible in Figure 2 of the main text. Same scale used as in the total map (Figure 38).

5.5 Measurements in the picosecond time scale

A complementary set of measurements was taken at longer time scales. Figures 42 and 44 present the maps of Urd (up to 150 ps) and 5mUrd (up to 5 ps). In both measurements, the transient spectra were recorded at magic angle.

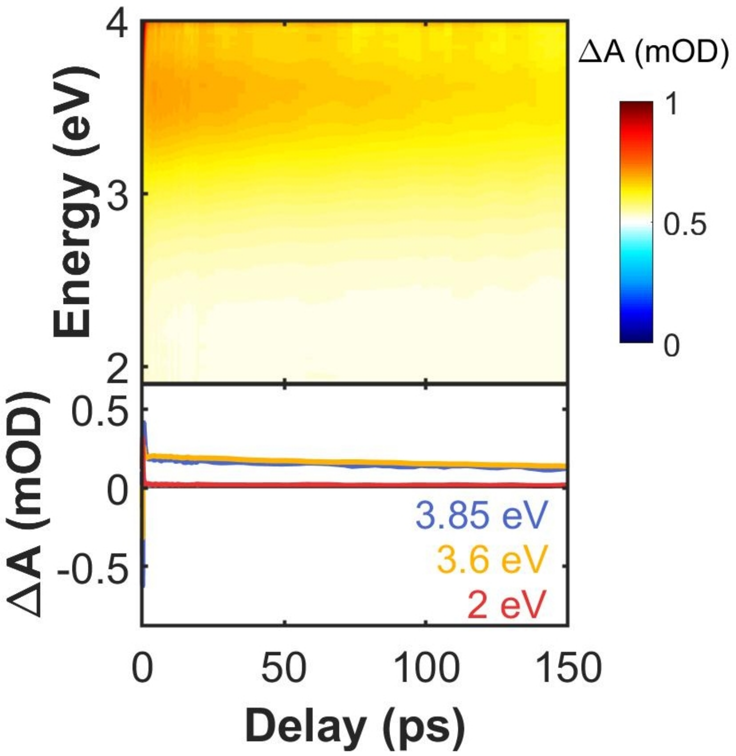

Supplementary Figure 42: Transient absorption spectra map and selected dynamics of Urd up to 150 ps.

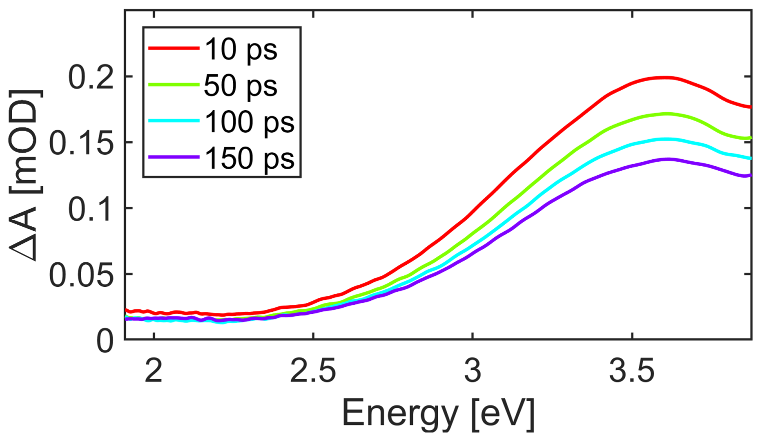

Supplementary Figure 43: Transient absorption spectra of Urd at selected time delays.

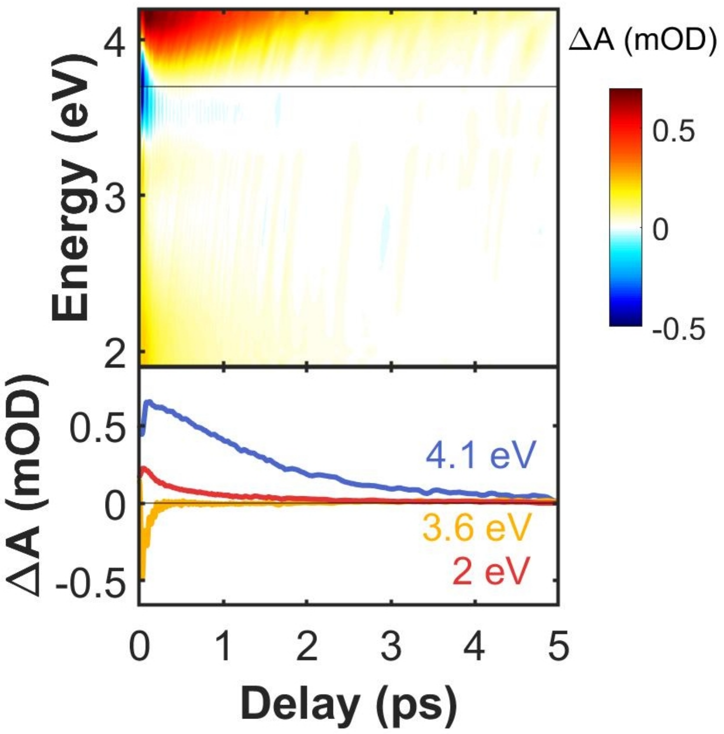

Supplementary Figure 44: Transient absorption spectra map and selected dynamics of 5mUrd up to 5 ps.

Supplementary Note 6: Data analysis

6.1 Fits at selected wavelengths

The following model was used to fit the experimental transient absorption dynamics at selected probe photon energies:

$$h(t) = g(t)+A_{rise}\left[1-\exp\left(-\frac{t}{\tau_{rise}}\right)\right]\left[A_1\exp\left(-\frac{t}{\tau_1}\right)+A_2\exp\left(-\frac{t}{\tau_2}\right)\right]H(t) \tag{24}$$

where $H(t) = u(t) \otimes f(t)$ is the convolution of the Heaviside function, u(t), and the instrumental response function, f(t). The latter was approximated by a Gaussian, whose temporal width was a free parameter in the model. The function g(t) models the coherent artifact [Lor02]:

$$g(t) = A_{art}\left[t.\exp\left[-2\left(\frac{t}{\tau}\right)^2\right]-(t-\tau_{GVD})\exp\left[-2\frac{(t-\tau_{GVD})^2}{\tau^2}\right]\right] \tag{25}$$

being τ the pump pulse duration and τ_{GVD} the difference of passage times through the medium between the pump and probe pulses.

The following table reports the time constants found for the dynamics of Urd reported in the main text. Note that we in addition we included an additional offset term to account for the hundreds of picoseconds signal.

Energy (eV)	τ_{rise}(fs)	τ_1 (fs)	τ_2 (fs)
2.0	–	125	–
3.6	–	80	1000
4.1	150	1000	–

Table 5: Time constants for dynamics at selected probe photon energies in Urd

The same procedure was applied for 5mUrd finding the following time constants:

Energy (eV)	τ_{rise}(fs)	τ_1 (fs)	τ_2 (fs)
2.0	–	135	1160
3.6	–	70	770
4.1	70	–	1600

Table 6: Time constants for dynamics at selected probe photon energies in 5mUrd

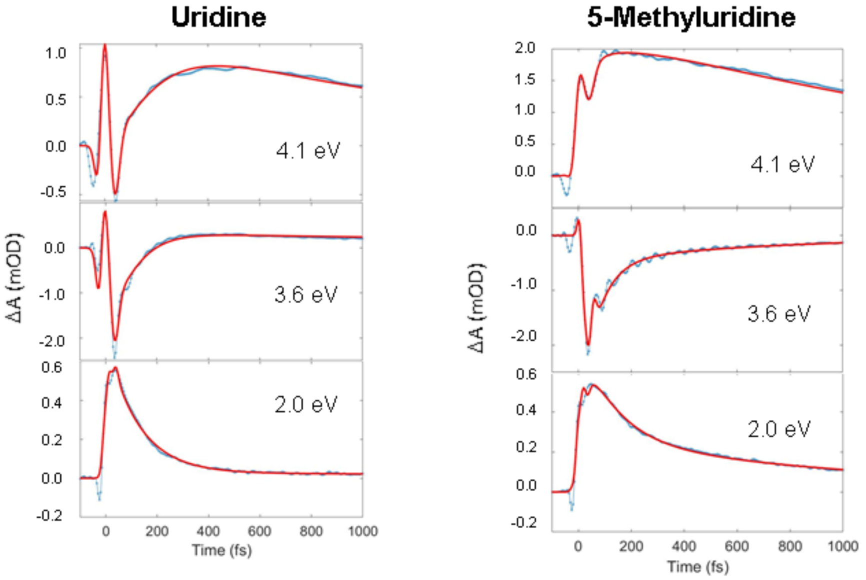

Supplementary Figure 45: Experimental data (blue) and fit (red) for selected dynamics in Urd (left) and 5mUrd (right).

6.2 Global analysis

6.2.1 Uridine (Urd)

A global analysis [Sto04, Sne12] using a sequential kinetic model was performed on the theoretical maps, which allowed us to assign the features in the evolution associated spectra (EAS) to the signatures of the SE and the different PAs:
a) Map containing only the PA signals from the S_1 state (right panel of Figure 40): a single component decaying with 130 fs time constant was found. We identify three excited-state absorption bands peaking at 4.4 eV, 3.35 eV and 1.75 eV.
b) Map containing only the SE signal from the S_1 state and the PA from the hot S_0 (left panel of Figure 40). We identify two main EAS corresponding to the SE from the S_1 state (black, peaking at 3.7 eV) and the PA from the hot S_0 (red, peaking at 4.4 eV). A minor component (dotted gray line) was necessary to finely adjust the fitting; we assign it to a slight redshift of the SE and the hot S_0 at early times.
c) Simulated pump-probe map containing all the signatures (Figure 1b of the main manuscript). The EAS of the S_1 state (black), composed by both the SE (3.7 eV) and the PAs (4.4 eV, 3.35 eV and 1.75 eV), decays with a time constant of 110 fs giving rise to the hot ground state absorption peaking at 4.4 eV (red). The latter is decaying beyond the time window of the simulations, which was limited to 500 fs.

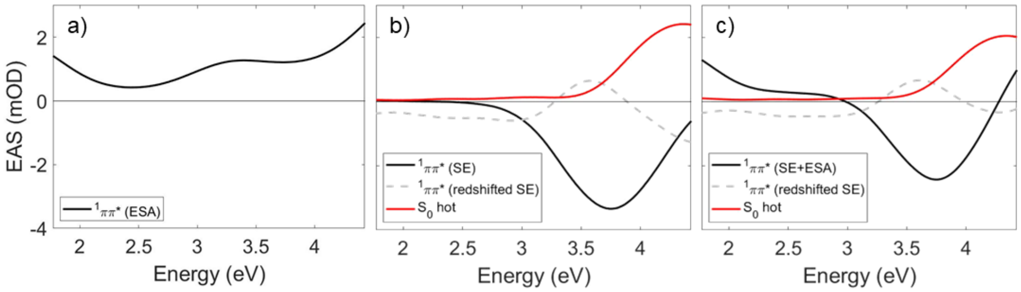

Supplementary Figure 46: EAS from the global fit for the simulated pump-probe maps containing: a) Only the PA from the S_1 state; b) only the SE signal from the S_1 state and the PA from the hot S_0; c) all the spectroscopic signals.

The same protocol was applied on the experimental data (Figure 1a of the main text).

We find that the experimental data are best described by four time constants: 97 fs (black), 480 fs (red), 550 fs (blue) and a long-living signal (green) decaying beyond the time window of the data, which was limited up to 5 ps.
The first two components match those of the EAS obtained from the computed map (Figure 46c). Hence we assigned the first EAS (black) to the S_1 state, whereas the second (red) is attributed mainly to the PA from the hot S_0.
Two additional components are found, which are not present in the theory: an EAS peaking at 4.2 eV (blue) and a long-living signal (green). We attribute the first one to the blue shifted PA from hot S_0. According to the reported oscillator strengths in literature [Pep18] we presume that the long-living signal could potentially arise from the $n\pi^*$ state and/or from the triplets.

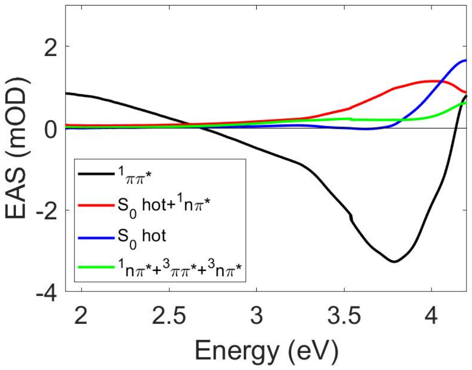

Supplementary Figure 47: EAS from the global fit of the experimental pump-probe map.

6.2.2 5-Methyluridine (5mUrd)

A global analysis using a sequential kinetic model was first performed on the theoretical maps:
a) Map containing only the PA signals from the S_1 state (right panel of Figure 41), describe by a single component decaying with 750 fs time constant. We identify three excited-state absorption bands peaking at 4.6 eV, 3.3 eV and 1.8 eV.
b) Map containing only the SE signal from the S_1 state and the PA from the hot S_0 (left panel of Figure 41). We identify three EAS corresponding to the SE from the S_1 state (black, peaking at 3.85 eV), the redshifted SE (red,

peaking at 3.4 eV) and the PA from the hot S_0 (blue, peaking above 4.4 eV). c) Simulated pump-probe map containing all the signatures (Figure 2b of the main manuscript). The EAS of the S_1 state (black), composed by both the SE (3.85 eV) and the PAs (4.6 eV, 3.3 eV and 1.8 eV), decays with a time constant of 190 fs giving rise to the redshifted SE (red) with an associated decay time constant of 270 fs. Finally, we observe a signal surviving beyond the observation window of the simulations (limited to 1 ps), which is attributed to the hot ground state absorption (blue).

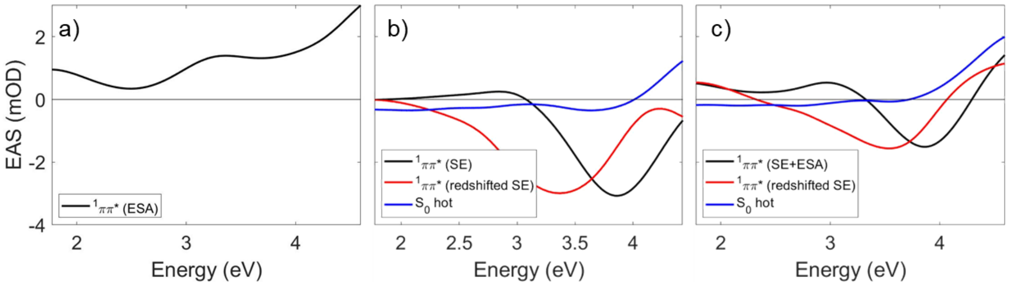

Supplementary Figure 48: EAS from the global fit for the simulated pump-probe maps containing: a) Only the PA from the S_1 state; b) only the SE signal from the S_1 state and the PA from the hot S_0; c) all the spectroscopic signals.

For the experimental data (Figure 2a of the main text), we find four components: 100 fs (black), 575 fs (red), 1.3 ps (blue) and a long-living signal (green) decaying beyond the limit of our observation window which was set to 5 ps.

The EAS with the shortest time constant (black) reproduces well that obtained from the simulations (Figure 48c) and it is thus attributed to the S_1 state. As for the second component (red EAS), we assign it to the redshifted SE (2.5-3.7 eV) and absorption of the hot ground state (peaking at 4 eV). We observe that the redshifted SE is more pronounced in the computed signals, meaning that the dynamics are slower in the simulations. The most probable reasons are:

i) the restriction of the movable solvent layer only to water molecules in 5 Å distance from the center of mass of the nucleoside, thus partially constraining (and hence slowing down) the rearrangement of the solvent;

ii) the need to exclude two low-frequency normal modes involving the methyl group during the Wigner sampling (see also chapter 2.3), thus effectively decreasing the kinetic energy at disposal.

Two additional EAS, not present in the simulated maps, are an EAS peaking at 4.2 eV (blue) and a low long-living signal (green). We attribute the first one to the blue shifted PA from hot S_0. According to the reported oscillator strengths in literature [Pep18] the long-living signal is probably due to the $n\pi^*$ state and/or from the triplets.

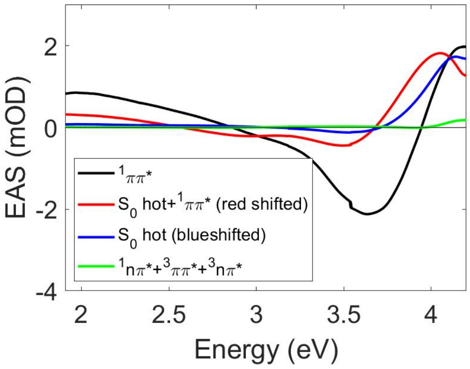

Supplementary Figure 49: EAS from the global fit of the experimental pump-probe map.

6.3 Excited-state vibrational modes

In order to extract the oscillatory pattern a Fourier low-pass filter was applied to the transient absorption spectra maps. The first 100 fs were removed in order to avoid artifacts in the 2D Fourier map due to the presence of the coherent artifact. To keep a good signal-to-noise ratio, we analyze a temporal window up to 400 fs in Urd and 1 ps in 5mUrd. The residual maps are shown in Figure 50. In both cases we observe a phase jump coinciding with the maximum of the SE signal, thus confirming that the oscillations belong to vibrational motions of the excited state wave-packet.

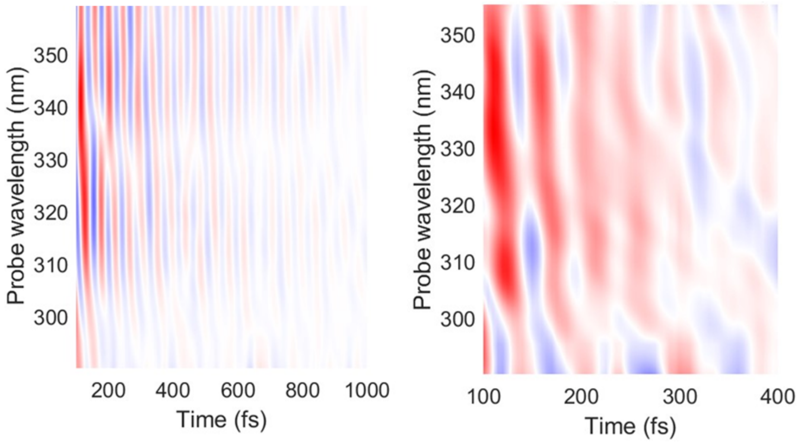

Supplementary Figure 50: Residuals showing an oscillatory pattern in 5mUrd (left) and Urd (right).

Supplementary Note 7: Analysis of the potential energy surfaces

7.1 Uridine (Urd)

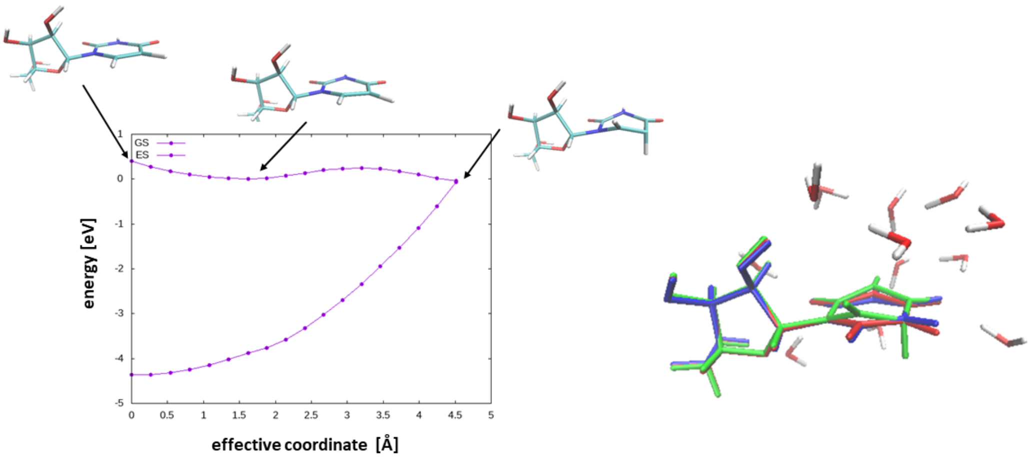

Supplementary Figure 51: Left: Potential energy scan between **FC**, $\mathbf{S1_{min}}$ and **CI** on the S_1 potential energy surface of Urd; Right: superimposed geometries of **FC**, $\mathbf{S1_{min}}$ and **CI** with waters in nearest vicinity (3.5 Å) to the nucleobase; note that the solvent arrangement was kept fixed in the optimizations.

By optimizing the reference[10] Urd geometry in *fixed* solvent at the same level of theory used for the dynamics simulation (i.e. SS-2-CASPT2/SA-2-CASSCF(10,8)/ANO-L[321,21]) we could locate a local minimum, labeled $\mathbf{S1_{min}}$, at which the ring is only slightly distorted from planarity (H-C-C-H = 6°) with a vertical gap of 3.88 eV (320 nm). We also optimized a conical intersection (CI) with the ground state (GS), labeled **CI**, which constitutes a local minimum, isoenergetic with $\mathbf{S1_{min}}$ (Figure 51, left), where we encounter the usual hydrogen out-of-plane (HOOP) puckering (H-C-C-H ∼ 90°). From the Franck-Condon (FC) point to the CI the geometrical deformations are exclusively localized in the nucleobase (Figure 51, right). An unrelaxed scan between $\mathbf{S1_{min}}$ and **CI** reveals a barrier of ca. 0.2 eV (Figure 51, left).

As a consequence of the Wigner sampling protocol which allows for the solvent to equilibrate for 100 ps (in order to remove the bias of the solvent's coordinates at the reference geometry, see 2.3) every generated structure has

[10]By reference we mean the snapshot selected from the MM simulation.

a different environment. Therefore, we studied to what extent the potential energy surface (PES) profile of the reference geometry is representative for the 57 snapshots used in the dynamics. Indeed, in fixed solvent optimizations we located in all but one[11] cases a local minimum $\mathbf{S1_{min}}$ with an average HOOP distortion of 10°and an average vertical energy gap of 3.79 eV (327 nm). Furthermore, we identified in all cases a S_0/S_1 CI lying on average 0.15 eV below the corresponding minimum.

Overall, the PES profiles demonstrate that the CI region can be reached by surmounting a small barrier and moreover, that no solvent reorganization is necessary. This facilitates the ultrafast decay of Urd (on a sub-100 fs time scale).

7.2 5-methyl-uridine (5mUrd)

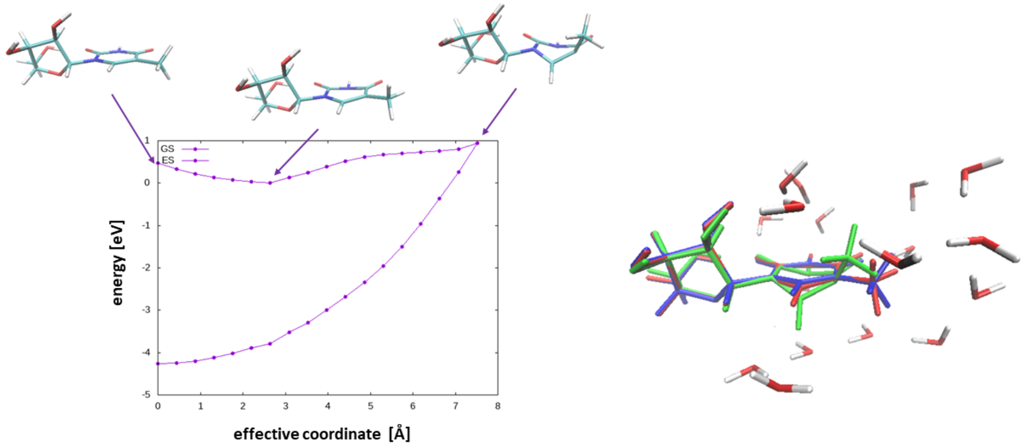

Supplementary Figure 52: Left: Potential energy scan between **FC**, $\mathbf{S1_{min}}$ and **CI** on the S_1 potential energy surface of 5mUrd; Right: superimposed geometries of **FC**, $\mathbf{S1_{min}}$ and **CI** with waters in nearest vicinity (3.5 Å) to the nucleobase; note that the solvent arrangement was kept fixed in the optimizations.

Similar analysis using the reference 5mUrd geometry also encountered a local minimum, labeled $\mathbf{S1_{min}}$ at which the ring is only slightly distorted from planarity (H-C-C-H = 8°) with a vertical gap of 3.79 eV (327 nm). A CI was also found, labeled **CI** (H-C-C-H $\sim$ 90°), however it lies ca. 1 eV higher in energy with respect to $\mathbf{S1_{min}}$ and does not correspond to a local

[11] In one case the optimization led straight to the CI with the ground state.

minimum (Figure 52, left)[12].

In order to rationalize the differing PES profiles for Urd and Thd, we allowed the solvent to equilibrate around the optimized $\mathbf{S1_{min}}$ and **CI** structures by allowing it to relax while keeping the coordinates of the nucleobase frozen. This lowered the barrier towards the CI to 0.6 eV. Simultaneous relaxation of solute and solvent led to a remarkable stabilization of **CI** relative to $\mathbf{S1_{min}}$ so that both geometries eventually become isoenergetic (Figure 53, left). A scan between the two geometries (green profile in Figure 52, left, selected structures shown in Figure 53, right) revealed a barrier of ca. 0.2 eV, similar to the one obtained for Urd. Thus, in the long time / slow dynamics limit the profiles of Urd and 5mUrd become very similar. This reconciles our findings with previous works that do not report significant differences between the PES of the two nucleosides[13].

We note that the stabilization of the CI region is associated with a favorable rearrangement of the near-by waters as shown in Figure 53, right. Thereby, we identified two waters, labeled **a** and **b** in Figure 53, right, which show pronounced displacements. Water **a** moves away (red⇒green) from the CH_3 group in order to leave space during the methyl out-of-plane deformation. Water **b** moves closer to the nucleobase in order to stabilize the negative charge forming on the pyramidalized carbon atom towards the CI.

The two PES profiles in 5mUrd, the one in *fixed* solvent (purple profile in Figure 53, left) and in *relaxed* solvent (green profile in Figure 53, left) should be regarded as the profiles the nucleoside feels in the limiting cases of ultrafast and slow dynamics with respect to the time scales of solvent rearrangement, respectively. Relaxation processes in water show time scales of $\sim$ 0.1 ps, $\sim$ 1 ps and $\sim$ 10 ps [Ben08]. Thereby, it is argued that the ultrafast time scale is associated with "*a structural fluctuation occurring over molecular length scales and within relatively fixed intermolecular configurations*". Thus, at early stages of the dynamics 5mUrd sees an strongly repulsive potential towards the CI region which explains why none of the trajectories reaches the CI region in the first 200 fs. Moreover, the only trajectory which decays within 200 fs to the GS is the one in which the C-N bond breaks, a pathway which does not require solvent rearrangement but is intrinsically associated with a barrier. On the time scale of several hundred femtoseconds the solvent adapts to the excited state electronic structure and the profile "flattens" allowing to reach the CI region in a virtually barrierless fashion.

Next, we studied to what extent the PES profile of the reference geometry

[12] In fact, minimum optimization from the CI leads back to $\mathbf{S1_{min}}$.

[13] We note that the profiles reported in Pepino et al. [Pep17] were obtained allowing the solvent to equilibrate.

is representative for all 57 snapshots used in the dynamics. We located in all cases a local minimum $\mathbf{S1_{min}}$ with an average methyl out-of-plane distortion of 8°and an average vertical energy gap of 3.80 eV (326 nm). Attempts to locate a minimum in the CI region for fixed solvent failed suggesting that the CI region is destabilized with respect to $\mathbf{S1_{min}}$, in concordance with the finding for the reference geometry.

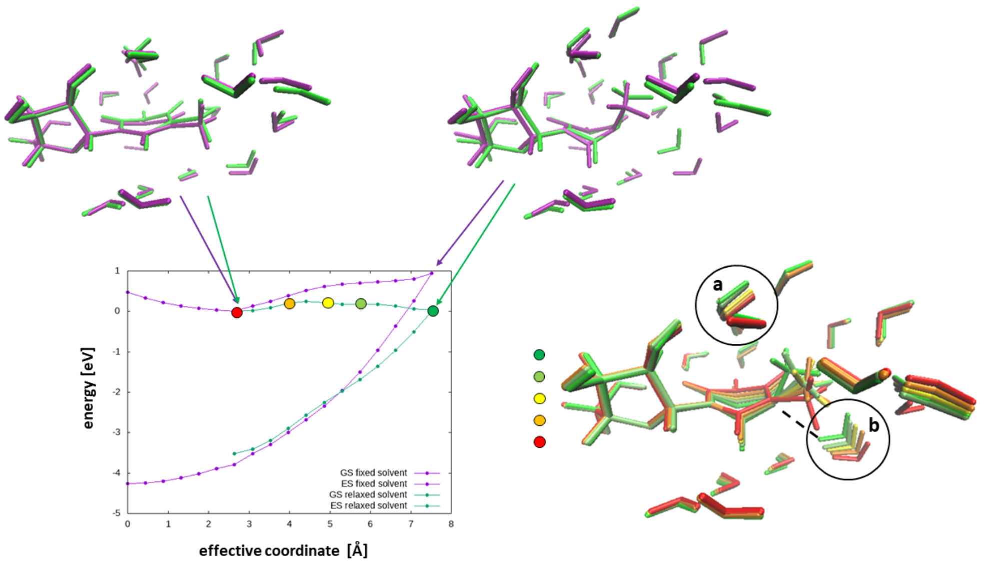

Supplementary Figure 53: Left: Potential energy scans between unrelaxed (purple line) and relaxed (green line) $\mathbf{S1_{min}}$ and **CI** on the S_1 potential energy surface of 5mUrd with corresponding geometries superimposed; Right: selected geometries along the scan between the relaxed $\mathbf{S1_{min}}$ and **CI**; waters showing most notable displacement are highlighted.

Notably, simultaneous relaxation of solute and near-by (5 Å) solvent leads to a red-shift of the average vertical energy gap by 20 nm (from 3.80 eV in fixed solvent to 3.57 eV in relaxed solvent). In several snapshots the red-shift is very strong (below 3.00 eV) and is accompanied by a pronounced methyl out-of-plane distortion[14]. These observations demonstrate that experimentally observed ultrafast red-shift of the stimulated emission (SE), accompanied by a pronounced intensity decay in the case of 5mUrd is, in fact, the spectral fingerprint of the coupled solvent-solute dynamics which facilitates the access to the CI region over a quasi-barrierless pathway on few hundred femtosecond time scale.

[14]We note, however, that in most structures the nucleobase remains predominantly planar so that the average value of the dihedral H-C-C-CH_3 = ±9°.

Supplementary Note 8: References

1. [Abr07] Abramavicius, D.; Valkunas, L.; Mukamel; S. Europhys. Lett. 2007, 80, 17005.
2. [Abr09] Abramavicius, D.; Palmieri, B.; Voronine, D. V.; Sanda, F.; Mukamel, S. Chem. Rev. 2009, 109, 2350-2408.
3. [Alt07] Altoè, P.; Stenta, M. ; Bottoni, A.; Garavelli M. Theor. Chem. Acc. 2007. 118, 219-240.
4. [Aqu09] Aquilante, F.; Pedersen, T. B.; Lindh R. Theor. Chem. Acc. 2009, 124, 1-10.
5. [Aqu16] Aquilante, F.; Autschbach, J.; Carlson, R.; Chibotaru, L.; Delcey, M. G.; De Vico, L.; Fernández Galvan,I. F.; Ferré, N.; Frutos, L. M.; Gagliardi, L.; Garavelli, M.; Giussani, A.; Hoyer, C.; Li Manni, G.; Lischka, H.; Ma, D.; Malmqvist, P.-Å.; Müller, T.; Nenov, A.; Olivucci, M.; Pedersen, T. B.; Peng, D.; Plasser, F.; Pritchard, P B.; Reiher, M.; Rivalta, I.; Schapiro, I.; Segarra-Martı, J.; Stenrup, M.; Truhlar, D. G.; Ungur, L.; Valentini, A.; Vancoillie, S.; Veryazov, V.; Vysotskiy, V.; Weingart, O.; Zapata, F.; Lindh, R. J Comput. Chem. 2016, 37, 506-541.
6. [Bar10] Barbatti, M.; Aquino, A. J. A.; Szymczak, J. J.; Nachtigallová, D.; Hobza, P.; Lischka, H. Proc. Natl. Acad. Sci. 2010, 107, 21453–21458.
7. [Bar13] Barc, B.; Ryszka, M.; Spurrell, J.; Dampc, M.; Limão-Vieira, P.; Parajuli, R.; Mason; N. J.; Eden, S. J. Chem. Phys. 2013, 139(24), 244311.
8. [Bea94] Bearpark M. J.; Robb M. A.; Schlegel H. B. Chem. Phys. Lett. 1994, 223, 269-274.
9. [Ben08] Beneduci, A. J. Mol. Liquids, 2008, 138, 55-60.
10. [Bor14] Borrego-Varillas, R.; Candeo, A.; Viola, D.; Garavelli, M.; De Silvestri, S.; Cerullo, G.; Manzoni, C. Opt. Lett. 2014, 39, 3849-3852.
11. [Bor15] Borrego-Varillas, R.; Oriana, A.; Branchi, F.; De Silvestri, S.; Cerullo, G.; Manzoni, C. J. Opt. Soc. Am. B, 2015, 32, 1851-1855.
12. [Bor16] Borrego-Varillas, R.; Oriana, A.; Ganzer, L.; Trifonov, A.; Buchvarov, I.; Manzoni, C.; Cerullo, G. Opt. Express, 2016, 24, 28491-28499.
13. [Bor18] Borrego-Varillas, R.; Ganzer, L.; Cerullo, G.; Manzoni, C. Appl. Sci., 2018, 8, 989.
14. [Bri19] Brister, M. M.; Crespo-Hernández, C. E. J. Phys. Chem. Lett., 2019, 10(9), 2156-2161.
15. [Buc15] Buchner, F., Nakayama, A., Yamazaki, S., Ritze, H.-H. ; Lübcke, A. J. Am. Chem. Soc. 2015, 137, 2931–2938.
16. [But12] Butkus, V.; Valkunas, L.; Abramavicius, D. J. Chem. Phys. 2012, 137, 044513.

17. [Cas12] Case, D. A.; Darden, T. A.; T. E. C. III, Simmerling, C. L.; Wang, J.; Duke, R. E.; Luo, R.; Walker, R. C.; Zhang, W.; Merz, K. M.; Roberts, B.; Hayik, S.; Roitberg, A.; Seabra, G.; Swails, J.; Götz A. W.; Kolossváry, I.; Wong, K. F.; Paesani, F.; Vanicek, J.; Wolf, R. M.; Liu, J.; Wu, X.; Brozell, S. R.; Steinbrecher, T.; Gohlke, H.; Cai, Q.; Ye, X.; Wang, J.; Hsieh, M.-J.; Cui, G.; Roe, D. R.; Mathews, D. H.; Seetin, M. G.; Salomon-Ferrer, R.; Sagui, C.; Babin, V.; Luchko, T.; Gusarov, S.; Kovalenko, A.; Kollman, P. AMBER 12, University of California, San Francisco, 2012.
18. [Can05] Canuel, C.; Mons, M.; Piuzzi, F.; Tardivel, B.; Dimicoli, I.; Elhanine, M. J. Chem. Phys. 2005, 122(7) 074316.
19. [Cer18] Cerezo, J.; Liu, Y.; Lin, N.; Zhao, X.; Improta, R.; Santoro, F. J. Chem. Theory Comput. 2018, 14(2), 820-832.
20. [Che13] Chen, J.; Thazhathveetil, A. K.; Lewis, F. D.; Kohler, B. J. Am. Chem. Soc., 2013, 135(28), 10290-10293.
21. [Che14] Chen, J.; Zhang, Y.; Kohler, B. Photoinduced phenomena in nucleic acids II. Springer, Cham, 2014. p. 39-87.
22. [Coh04] Cohen, B., Crespo-Hernández, C. E. ; Kohler, B. Faraday Discuss. 2004, 127, 137–147.
23. [Con18] Conti, I. ; Garavelli, M. J. Phys. Chem. Lett. 2018, 9, 2373–2379.
24. [Cre04] Crespo-Hernández, C. E.; Cohen, B.; Hare, P. M.; Kohler, B. Chem. Rev. 2004, 104, 1977–2019.
25. [Du15] Du, L.; Lan, Z. J. Chem. Theory Comput., 2015, 11, 1360.
26. [Eri19] Erickson, B. A.; Heim, Z. N.; Pieri, E.; Liu, E.; Martínez, T. J.; Neumark, D. M. J. Phys. Chem. A, 2019, 123(50), 10676-10684.
27. [Fei01] Feig, M.; Karanicolas, J.; Brooks, C. L., III: MMTSB Tool Set (2001), MMTSB NIH Research Resource, The Scripps Research Institute.
28. [Fer12] Ferrer, F. J. A.; Santoro, F. Phys. Chem. Chem. Phys., 2012, 14, 13549-13563.
29. [Fer13] Salomon-Ferrer, R.; Case, D. A.; Walker, R. C., WIREs Comput. Mol. Sci., 2013, 3, 198-210.
30. [Fin13] Fingerhut, B. P.; Dorfman, K. E.; Mukamel, S. J. Phys. Chem. Lett. 2013, 4, 1933–1942.
31. [Fin14] Fingerhut, B. P.; Dorfman, K. E.; Mukamel, S. J. Chem. Theory Comput. 2014, 10, 1172–1188.
32. [For97] Forsberg, N.; Malmqvist, P. A. Chem. Phys. Lett. 1997, 274, 196-204.
33. [Fri10] Frisch, M. J.; Trucks, G. W.; Schlegel, H. B.; Scuseria, G. E.; Robb, M. A.; Cheeseman, J. R.; Scalmani, G.; Barone, V.; Mennucci, B.; Petersson, G. A.; Nakatsuji, H.; Caricato, M.; Li, X.; Hratchian, H. P.; Iz-

maylov, A. F.; Bloino, J.; Zheng, G.; Sonnenberg, J. L.; Hada, M.; Ehara, M.; Toyota, K.; Fukuda, R.; Hasegawa, J.; Ishida, M. Nakajima, T.; Honda, Y.; Kitao, O.; Nakai, H.; Vreven, T.; Montgometry Jr, J. A.; Peralta, J. E.; Ogliaro, F.; Bearpark, M.; Heyd, J. J.; Brothers, E.; Kudin, K. N.; Staroverov, V. N.; Kobayashi, R.; Normand, J.; Raghavachari, K.; Rendell, A.; Burant, J. C.; Iyengar, S. S.; Tomasi, J.; Coss, M.; Rega, N.; Millam, J. M.; Klene, M.; Knox, J. E.; Cross, J. B.; Bakken, V.; Adamo, C.; Jaramillo, J.; Gomperts, R.; Stratmann, R. E.; Yazyev, O.; Austin, A. J.; Cammi, R.; Pomelli, C.; Ochterski, J. W.; Martin, R. L.; Morokuma, K.; Zakrzewski, V. G.; Voth, G. A.; Salvador, P.; Dannenberg, J. J.; Dapprich, S.; Daniels, A. D.; Farkas, O.; Foresman, J. B.; Ortiz, J. V.; Cioslowski, J.; Fox, D. J. Gaussian 09 Rev. B01, Gaussian, Inc., Wallingford, CT, 2010.

34. [Gha18] Ghafur, O.; Crane, S. W.; Ryszka, M.; Bockova, J.; Rebelo, A.; Saalbach, L.; De Camillis, S.; Greenwood, J. B.; Eden, S.; Townsend, D. J. Chem. Phys. 2018, 149, 034301.

35. [Ghi04] Ghigo, G.; Roos, B. O.; Malmqvist, P. A. Chem. Phys. Lett. 2004, 396, 142-149.

36. [Gon10] González-Luque, R.; Climent, T.; González-Ramírez, I.; Merchán, M.; Serrano-Andrés, L. J. Chem. Theory Comp., 2010, 6(7), 2103-2114.

37. [Goz14] Gozem, S.; Melaccio, F.; Valentini, A.; Filatov, M.; Huix-Rotllant, M.; Ferré, N.; Frutos, L. M.; Angeli, C.; Krylov, A. I.; Granovsky, A. A.; Lindh, R.; Olivucci, M. J. Chem. Theory Comput. 2014, 10 (8), 3074-3084.

38. [Gra11] Granovsky, A. A. J. Chem. Phys., 2011, 134, 214113.

39. [Gra07] Granucci, G.; Persico, M. J. Chem. Phys., 2007, 126 (13), 134114.

40. [Gus02] Gustavsson, T.; Sharonov, A.; Markovitsi Chem. Phys. Lett. 2002 351, 195–200.

41. [Gus06] Gustavsson, T. J. Am. Chem. Soc. 2006, 128, 607–619.

42. [Gus11] Gustavsson, T.; Banyasz, A.; Improta, R.; Markovitsi, D. J. Phys. Conf. Ser. 2011, 261.

43. [Ham11] Hamm, P.; Zanni, M. Concepts and Methods of 2d Infrared Spectroscopy; Cambridge University Press: Cambridge, U.K., 2011.

44. [Ham94] Hammes-Shiffer, S.; Tully, J. C. J. Chem. Phys., 1994, 101, 4657.

45. [Har07] Hare, P. M.; Crespo-Hernández, C. E.; Kohler, B. Proc. Natl. Acad. Sci. 2007, 104, 435–440.

46. [Har08] Harald Nieber, N. L. D. Chem. Phys. 347, 405–412 (2008).

47. [He03] He, Y.; Wu, C.; Kong, W. J. Phys. Chem. A 2003, 107, 5145–5148.

48. [He04] He, Y.; Wu, C.; Kong, W. J. Phys. Chem. A 2004, 108, 943–949.

49. [Hoch01] Hochstrasser, R. M. Chem. Phys., 2001, 266, 273-284.
50. [Imp09] Improta, R.; Barone, V.; Lami, A.; Santoro, F. J. Phys. Chem. B 2009, 113, 14491–14503.
51. [Imp16] Improta, R.; Santoro, F.; Blancafort, L. Chem. Rev. 2016, 116, 3540–3593.
52. [Jor83] Jorgensen, W. L.; Chadrasekhar, J.; Madura, J. D.; Impey, R. W.; Klein, M. L. J. Chem. Phys., 1983, 79, 926.
53. [Kob12] Kobayashi, T. ; Kida, Y. Phys. Chem. Chem. Phys. 2012, 14, 6200.
54. [Koh10] Kohler, B. J. Phys. Chem. Lett. 2010, 1, 2047–2053.
55. [Kur01] Kurtz, L.; Hofmann, A.; de Vivie-Riedle, R. J. Chem. Phys. 2001, 114, 6151-6159.
56. [Kur01b] Kurtz, L. Disseration at the LMU Müenchen, 2001.
57. [Kwo08] Kwok, W. M.; Ma, C.; Phillips, D. L. J. Am. Chem. Soc. 2008, 130, 5131–5139.
58. [Lee16] Lee, M. K.; Huo, P.; Coker; D. F. Annu. Rev. Phys. Chem. 2016. 67:639-68.
59. [Li94] Li, B.; Johnson, A. E.; Mukamel, S.; Myers, A. B. J. Am. Chem. Soc. 1994,116, 11039-11047.
60. [Liu18] Liu, Y.; Cerezo, J.; Lin, N.; Zhao, X.; Improta, R.; Santoro, F. Theo. Chem. Acc. 2018, 137, Article number: 40.
61. [Lis18] Lischka H.; Barbatti, M.; Siddique, F.; Dasa Adelia, A.; J.A.Aquino Chem. Phys. 2018, 515, 472–479.
62. [Lor02] Lorenc, M.; Ziolek, M.; Naskrecki, R.; Karolczak, J.; Kubicki, J.; Maciejewski, A. Appl. Phys. B Lasers Opt. 2002, 74, 19–27.
63. [Mai16] Mai, S.; Marquetand, P.; Gonzalez, L. J. Phys. Chem. Lett. 2016, 7 (11), 1978-1983.
64. [Mai17] Mai, S.; Richter, M.; Marquetand, P.; González, L. Chem. Phys. 2017, 482, 9–15.
65. [Mal86] Malmqvist, P.Å. Int. J. Quantum Chem., 1986, 30, 479-494.
66. [Mid09] Middleton, C. T.; de La Harpe, K.; Su, C.; Law, Y. K.; Crespo-Hernández, C. E.; Kohler, B. Annu. Rev. Phys. Chem., 2009, 60, 217.
67. [McN97] IUPAC. Compendium of Chemical Terminology, 2nd ed. (the "Gold Book"). Compiled by A. D. McNaught and A. Wilkinson. Blackwell Scientific Publications, Oxford (1997). Online version (2019-) created by S. J. Chalk. ISBN 0-9678550-9-8. https://doi.org/10.1351/goldbook].
68. [Miy92] Miyamoto, S.; Kollman, P. A. J. Comput. Chem., 1992, 13, 952-962.
69. [Muk95] Mukamel, S. Principles of Nonlinear Optical Spectroscopy; Oxford University Press: New York, 1995.
70. [Muk04] Mukamel, S.; Abramavicius, D. Chem. Rev. 2004, 104, 2073-

2098.
71. [Nac11] Nachtigallová, D.; Aquino, A. J. A.; Szymczak, J. J.; Barbatti, M.; Hobza, P.; Lischka, H. J. Phys. Chem. A 2011, 115, 5247–5255.
72. [Nak13] Nakayama, T., Arai, G., Yamazaki, S.; Taketsugu, T. J. Chem. Phys. 2013, 139, 214304.
73. [Nem10] Nemeth, A.; Milota, F.; Mančal, T.; Pullerits, T.; Sperling, J.; Hauer, J.; Kauffmann, H. F.; Christensson N. J. Chem. Phys. 2010, 133, 094505.
74. [Oni02] Onidas, D.; Markovitsi, D.; Marguet, S.; Sharonov, A.; Gustavsson, T. J. Phys. Chem. B 2002, 106, 11367–11374.
75. [Par19] Parker, S. M.; Roy, S.; Furche, F. Phys. Chem. Chem. Phys., 2019, 21(35), 18999-19010.
76. [Pec00] Pecourt, J. M. L.: Peon, J.; Kohler, B.; J. Am. Chem. Soc. 2000, 122, 9348–9349.
77. [Pec01] Pecourt, J. M. L.; Peon, J.; Kohler, B.; J. Am. Chem. Soc. 2001, 123, 10370–10378.
78. [Peo01] Peon, J.; Zewail, A. H. Chem. Phys. Lett. 2001, 348, 255–262.
79. [Pep17] Pepino, A. J.; Segarra-Martí, J.; Nenov, A.; Improta, R.; Garavelli, M. j. phys. chem. lett., 2017, 8(8), 1777-1783.
80. [Pep18] Pepino, A. J.; Segarra-Martí, J.; Nenov, A.; Rivalta, I.; Improta, R.; Garavelli, M. Phys. Chem. Chem. Phys., 2018, 20(10), 6877-6890.
81. [Per06] Perun, S.; Sobolewski, A. L.; Domcke, W. J. Phys. Chem. A 2006, 110, 13238–13244
82. [Pil18] Pilles, B. M.; Maerz, B.; Chen, J.; Bucher, D. B.; Gilch, P.; Kohler, B.; Zinth, W.; Fingerhut, B. P.; Schreier, W. J. J. Phys. Chem. A 2018, 122, 4819–4828.
83. [Pit09] Pittner, J.; Lischka, H.; Barbatti, M. Chem. Phys., 2009, 356, 147.
84. [Pol14] Pollum, M., Martínez-Fernández, L. ; Crespo-Hernández, C. E. Photochemistry of Nucleic Acid Bases and Their Thio- and Aza-Analogues in Solution. in Top. Curr. Chem. 245–327 (Springer, 2014).
85. [Pro16] Prokhorenko, V. I.; Picchiotti, A.; Pola, M.; Dijkstra, A. G.; Miller, R. J. D. D. J. Phys. Chem. Lett. 2016, 7, 4445–4450.
86. [Rei18] Reiter, S.; Keefer, D.; de Vivie-Riedle, R. J. Am. Chem. Soc. 2018, 140, 8714–8720.
87. [Ric14] Richter, M.; Mai, S.; Marquetand, P.; González, L. Phys. Chem. Chem. Phys. 2014, 16, 24423–24436.
88. [Ryc77] Ryckaert, J.-P.; Ciccotti, G.; Berendsen, H. J. C. J. Comput. Phys., 1977, 23, 327-341.
89. [Sai07] Saigusa, H. J. Photochem. 2007, 7, 197–210.
90. [San09] Santoro, F.; Improta, R.; Barone, V. Theor. Chem. Acc. 2009,

123, 273–286.
91. [Ser05] Serrano-Andrés, L.; Merchán, M.; Lindh, R. J. Chem. Phys. 2005, 122, 104107.
92. [Ser07] Serrano-Pérez, J. J.; González-Luque, R.; Merchán, M.; Serrano-Andrés, L. J. Phys. Chem. B 2007, 111, 11880–11883.
93. [Shi11] Shiozaki, T.; Györffy, W.; Celani, P.; Werner, H.-J. J. Chem. Phys., 2011, 135, 081106.
94. [Sne12] Snellenburg, J. J.; Laptenok, S. P.; Seger, R.; Mullen, K. M.; van Stokkum, I. H. M. J. Stat. Softw. 2012, 49 (3).
95. [Sto16] Stojanović, L.; Bai, S.; Nagesh, J.; Izmaylov, A. F.; Crespo-Otero, R.; Lischka, H.; Barbatti, M. Molecules 2016, 21, 1–14.
96. [Tul90] Tully, J. C. Faraday Discus., 1998, 110, 407-419.
97. [Val16] Valkunas, L.; Abramavicius, D.; Mančal, T. Molecular Excitation Dynamics and Relaxation, Wiley-VCH Verlag GmbH ; Co. KGaA, 2016.
98. [vanSto04] van Stokkum, I. H. M., Larsen, D. S., van Grondelle, R. Biochim Biophys Acta - Bioener 2004, 1657 (2-3), 82-104.
99. [Wid90] Widmark, P.-O.; Malmqvist, P-Å.; Roos., B. O. Theor. Chim. Acta 1990, 77, 291.
100. [Wol19] Wolf, T. J.; Parrish, R. M.; Myhre, R. H.; Martínez, T. J.; Koch, H.; Gühr, M. J. Phys. Chem. A, 2019, 123(32), 6897-6903.
101. [Xue16] Xue, B., Yabushita, A. ; Kobayashi, T. Phys. Chem. Chem. Phys. 2016, 18, 17044–17053.
102. [Yu16] Yu, H.; Sanchez-Rodriguez, J. A.; Pollum, M.; Crespo-Hernández, C. E.; Mai, S.; Marquetand, P.; González, L.; Ullrich, S. Phys. Chem. Chem. Phys. 2016, 18, 20168–20176.
103. [Zhu05] Zhu, C.; Jasper, A. W.; Truhlar, D. G. J. Chem. Theory Comput., 2005, 1 (4), 527-540.
